# Commercial Plant Production and Consumption Still Follow the Latitudinal Gradient in Species Diversity despite Economic Globalization

**DOI:** 10.1371/journal.pone.0163002

**Published:** 2016-10-05

**Authors:** Erik J. Nelson, Matthew R. Helmus, Jeannine Cavender-Bares, Stephen Polasky, Jesse R. Lasky, Amy E. Zanne, William D. Pearse, Nathan J. B. Kraft, Daniela A. Miteva, William F. Fagan

**Affiliations:** 1 Department of Economics, Bowdoin College, Brunswick, Maine, United States of America; 2 Center for Biodiversity, Department of Biology, Temple University, Philadelphia, Pennsylvania, United States of America; 3 Department of Ecological Sciences—Animal Ecology, Vrije Universiteit, Amsterdam, Netherlands; 4 Department of Ecology, Evolution, and Behavior, University of Minnesota, Saint Paul, Minnesota, United States of America; 5 Institute on Environment, University of Minnesota, Saint Paul, Minnesota, United States of America; 6 Department of Applied Economics, University of Minnesota, Saint Paul, Minnesota, United States of America; 7 Earth Institute, Columbia University, New York, New York, United States of America; 8 Department of Ecology, Evolution, and Environmental Biology, Columbia University, New York, New York, United States of America; 9 Department of Biological Sciences, George Washington University, Washington, D.C., United States of America; 10 Department of Biology, University of Maryland, College Park, Maryland, United States of America; 11 Department of Agricultural, Environmental, and Development Economics, The Ohio State University, Columbus, Ohio, United States of America; 12 National Socio-environmental Synthesis Center (SESYNC), University of Maryland, Annapolis, Maryland, United States of America; Missouri Botanical Garden, UNITED STATES

## Abstract

Increasing trade between countries and gains in income have given consumers around the world access to a richer and more diverse set of commercial plant products (i.e., foods and fibers produced by farmers). According to the economic theory of comparative advantage, countries open to trade will be able to consume more–in terms of volume *and* diversity–if they concentrate production on commodities that they can most cost-effectively produce, while importing goods that are expensive to produce, relative to other countries. Here, we perform a global analysis of traded commercial plant products and find little evidence that increasing globalization has incentivized agricultural specialization. Instead, a country’s plant production and consumption patterns are still largely determined by local evolutionary legacies of plant diversification. Because tropical countries harbor a greater diversity of lineages across the tree of life than temperate countries, tropical countries produce and consume a greater diversity of plant products than do temperate countries. In contrast, the richer and more economically advanced temperate countries have the capacity to produce and consume more plant species than the generally poorer tropical countries, yet this collection of plant species is drawn from fewer branches on the tree of life. Why have countries not increasingly specialized in plant production despite the theoretical financial incentive to do so? Potential explanations include the persistence of domestic agricultural subsidies that distort production decisions, cultural preferences for diverse local food production, and that diverse food production protects rural households in developing countries from food price shocks. Less specialized production patterns will make crop systems more resilient to zonal climatic and social perturbations, but this may come at the expense of global crop production efficiency, an important step in making the transition to a hotter and more crowded world.

## Introduction

One of the most striking biological patterns on Earth is the latitudinal gradient in biodiversity, where the tropics produce more species and evolutionary lineages than the temperate zone [1]. Plants, in particular, show an increasing biodiversity trend towards the equator [2]. Until recently, this pattern also largely limited the plant diversity any one society could regularly produce and consume. Only since the mid-1600s, when ships started to regularly transport food, seeds, and botanical knowledge between continents, were societies able to loosen the very tight relationship between their environment and the plants they produced (e.g., the transfer of potato production from Peru to Ireland) and consumed (e.g., the import of pineapples to Europe from Suriname) [3]. For example, between 1760 and the 1850s the value of tropical food imported to Great Britain increased by 384% [4]. More recently, the green revolution in agricultural production [5], lower tariffs on imports [6], and more cost-effective trading technology [5–8] have made it much easier for modern societies to consume a diversity of plants far beyond their pre-modern plant diversity.

Economists have long examined how international trade affects a country’s commodity production and consumption patterns. According to comparative advantage theory, countries open to trade will become wealthier if they concentrate production on commodities that they can most cost-effectively produce relative to other countries, while importing goods that they find relatively expensive to produce [9]. In this way, countries can consume more–in terms of volume *and* diversity–than they would be able to do otherwise [10]. As a country connects to even more countries, the set of commodities in which it has a comparative advantage will shrink even further. Yet, the country can continue to grow and consume an even greater volume and diversity of goods if it produces and trades according to its remaining comparative advantages. Therefore, increasing openness to trade should create incentives and the capability to specialize in production but diversify in consumption.

However, comparative advantage considerations are not the only factors that determine what a country produces domestically and what it trades. Government policy can prevent behavior that would be consistent with comparative advantage theory. For example, a country’s trade policies could impede its consumers from accessing foreign markets, meaning its firms must produce a wider range of goods to satisfy domestic demand, including those goods they produce relatively inefficiently. Or a country’s industrial policy could include heavy subsidization of goods in which the country does not have a comparative advantage. Moreover, developing country farmers may maintain diverse production as insurance against volatile local food prices or because they lack access to the credit markets or technology that would enable them switch to cash-crop production [11]. Finally, farmers around the world may be hesitant to change cropping practices due to culture or tradition.

In addition, a trend in increased production specialization in reaction to greater trade openness could be obscured by the impact of increasing incomes on a country’s consumption and production decisions and capabilities. Consumers with low incomes concentrate on necessities, but as their incomes increase, non-necessities become a growing share of their consumption [12,13]. For example, the demand for a diversity of food varieties and brands, including more expensive exotic foodstuffs, increases with income [14–16]. Although these diversified foods are produced more cheaply elsewhere, local entrepreneurs may take advantage of market imperfections, barriers to trade, and increasing consumer preferences for local food [17] to find a niche in the markets that cater to preferences for food variety. Finally, as countries get richer, their technical efficiency and, thus, their capability to generate a wider variety of goods increases [18,19]. In short, government policy, market imperfections, and growing incomes and capabilities can blunt a country’s ability or imperative to produce and trade according to comparative advantage.

Our first goal is to test for the expected effects from changes in country-level trade openness, income, and government policy on the phylogenetic diversity of commercial plant production and consumption as postulated by comparative advantage theory. Our second goal is to investigate how latitude–our proxy for climate and plant biogeographic history–affects and mediates levels and trends in the diversity and richness of commercial plant production and consumption.

Regarding the first test, previous cross-sectional statistical analysis has shown that countries have tended to produce crops with traits best suited for their unique agronomic conditions [20]. We test the hypothesis that this cost-effectiveness strategy is accentuated over time by increasing connectedness to trading partners despite the many aforementioned potential barriers to increasing specialization. Further, we test the hypothesis that wealthier consumers with better connections to the rest of the world increasingly sample a diversity of the world’s foods and fibers.

Regarding our second goal, we ask the following questions: How different are the tropical and temperate zones in the levels of commercial plant production and consumption diversity and richness? For example, as we see with production in general [18,19], do the more complex economies of the temperate zone produce more commercial plant species than the less complex economies of the tropical zone? Further, do we see evidence of convergence in the diversity and richness of commercial plants produced and consumed across the tropical and temperate zones? Finally, do producers at lower latitudes react differently to gains in trade openness than producers at higher latitudes? Do consumers at lower latitudes react differently to gains in incomes than consumers at higher latitudes [21]?

The novelty of our analysis is predicated on finding representative metrics of plant production and consumption diversity. In addition to using the count of plant species produced or consumed within a country (species richness (SR)), which is often a flawed metric of commodity variety [22], we use phylogenetic diversity [23–25], as measured by the phylogenetic species variability (PSV) metric ([26], eq. A in S1 File), to measure functional variety. PSV gives the average evolutionary relatedness of a set of species; in other words, PSV allows us to capture similarities in the attributes of goods produced or consumed. Therefore, a change in a country’s produced PSV over time should indicate whether the country is increasingly narrowing or broadening its commercial plant production, presumably according to its unique agro-climate conditions. For example, suppose a country that was producing 7 cereal species and 5 stone fruit species transitions to producing 12 cereal species and no stone fruits as it becomes more open to trade. PSV, but not SR, would pick up this specialization in production, as cereals are all from the same grass family *Poaceae*. Further, it has been found that differences in plant characteristics, including many that consumers care about, such as taste, texture, and nutritional mix, are broadly captured by differences in the evolutionary history of plant lineages [27,28]. Because of this relationship, the PSV of a basket of consumed plants provides an important proxy for the average consumed trait diversity. Therefore, a change in a country’s consumed PSV over time will indicate whether the country is increasingly narrowing or broadening the variety of plant traits it consumes.

We compliment the SR and PSV metrics with Simpson’s evenness, symbolized by E, a non-phylogenetic metric that weighs richness by the amount of each species produced or consumed [29]. This metric allows us to account for the more nuanced cases of specialization–countries do not completely eliminate species production but instead heavily concentrate on a more limited set of species–or diversification–consumers do not necessarily increase the number of species consume but their basket becomes more even in the distribution of goods it does consume.

For our three metrics of diversity and richness we make the following predictions (Table 1):

1. All else equal, production PSV and E are negatively correlated with a country’s openness to trade. We expect the rewards to specializing on a narrower range of items with similar traits to have increased as a country becomes more economically connected with the rest of the world.
2. On average, a country’s consumption PSV and SR increase as the country’s per capita income and openness to trade increase, because richer consumers demand more diversity [10, 11–12, 24, 30–32] and the costs of consuming exotic diversity fall in trade openness. Further, on average, the evenness of consumption, E, increases with openness to trade, because connectivity to the rest of the world gives the country the ability to find alternative sources of plants when the supply from a usual source is temporarily disrupted.
3. Based on (1) and (2), we hypothesize that the gaps between consumed and produced PSV, SR, and E *within* a country increase with better connectivity to the rest of the world and income.

**Table 1:** Summary of Hypotheses.

	Drivers of change	
	**Change in trade openness**	**Change in per capita GDP**
	NEGATIVE	POSITIVE
**Change in plant production**	• As a country becomes more open to trade its production of phylogenetic diversity (PSV), production of species richness (SR), and evenness of produced species richness (E) falls.	• As a country becomes richer, its capacity to produce a more diverse and richer set of plants increases, all else equal. In other words, production PSV and SR increase in per capita income.
**Change in plant consumption**	POSITIVE	POSITIVE
	• As a country becomes more open to trade it consumes more plant phylogenetic diversity and richness, all else equal. In other words, consumption PSV and SR increase with trade openness. • The positive impact of trade openness on consumption PSV is greater in high latitude countries as these countries have to trade for greater access to phylogenetic diversity. • The impact of trade openness on consumption E is positive because increased connectivity to the rest of the world gives a country the ability to find alternative sources of plants when supply from a usual source is temporarily disrupted.	• As a country becomes richer, it consumes more phylogenetic diversity and richness. In other words, consumption PSV and SR increase with per capita income. • The positive effect of increasing income on consumption PSV is stronger in high latitude countries as greater income gives these countries greater capacity to consume beyond their climatic limitations. • The impact of per capita income on consumption E is positive as greater income gives a country greater capacity to find alternative sources of plants when supply from a usual source is temporarily disrupted.

Table 1 also contains hypotheses on how latitude mediates the impact of changes in trade openness and incomes on diversity and richness trends.

We start by examining the temporal trends in production and consumption PSV, SR, and E in the tropical and temperate zones since 1992. Then we test whether changes in country-level production and consumption PSV, SR, and E vary with country latitude and changes in country-level per capita income, trade openness, and government intervention in the agricultural sector [33]. Our spatio-temporal analysis of plant production and consumption diversity and richness allows us to determine the extent to which trade openness and increasing income has allowed countries to overcome the environmentally determined patterns of plant diversity from the tropics to the temperate zone.

## Results

### Country-level temporal trends in produced and consumed PSV, SR and E

**Fig 1:**
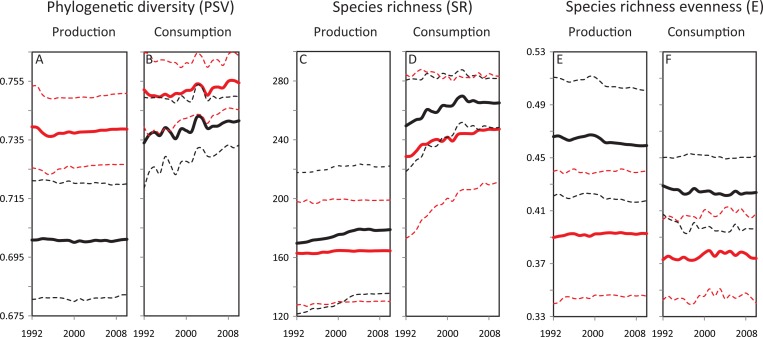
Trends in country-level diversity metrics from 1992 to 2010 for temperate (black) and tropical (red) countries. Solid lines are zonal weighted averages and dashed lines are plus and minus one standard deviation from the zone’s average. The weighted average of metric *y* in year *t* in zone *r* is $\mu_{tr}^{*}=\sum_{k\in \in r}^{K}w_{kt}y_{kt}/\sum_{k\in \in r}^{K}w_{kt}$ where *w*_*kt*_ is country *k*’s arable land in in year *t* for production metrics and the country’s population in year *t* for consumption metrics. The weighted standard deviation of metric *y* in year *t* for zone *r* is given by $\sigma_{tr}^{*}={\left(\sum_{k\in r}^{K}w_{kt}{\left(y_{kt}-\mu_{tr}^{*}\right)}^{2}/\sum_{k\in r}^{K}w_{kt}\right)}^{0.5}$. Differences in weighted mean trends across zones. ${\text{H}}_{0}:\mu_{t,tropics}^{*}-\mu_{t,temp}^{*}=0$ and degrees of freedom (df) are 18. **(A)** t-stat = 172.2, P(T ≤ t) = 2.1x10^-30^; **(B)** t-stat = 33.6, P(T ≤ t) = 1.1x10^-17^; **(C)** t-stat = –17.8, P(T ≤ t) = 7.2x10^-13^; **(D)** t-stat = –36.2, P(T ≤ t) = 2.9x10^-18^; **(E)** t-stat = –83.6, P(T ≤ t) = 9.1x10^-25^; **(F)** t-stat = –55.3, P(T ≤ t) = 1.5x10^-21^. Differences in weighted mean production and consumption trends. ${\text{H}}_{0}:\mu_{t,r,consumption}^{*}-\mu_{t,r,prodcution}^{*}=0$ and df are 18. Temp. PSV: t-stat = 66.5, P(T ≤ t) = 5.6x10^-23^; Temp. SR: t-stat = 131.8, P(T ≤ t) = 2.5x10^-28^; Temp. E: t-stat = –72.3, P(T ≤ t) = 1.2x10^-23^; Trop. PSV: t-stat = 40.3, P(T ≤ t) = 4.3x10^-19^; Trop. SR: t-stat = 63.6, P(T ≤ t) = 1.2x10^-22^; and Trop. E: t-stat = –34.9, P(T ≤ t) = 5.4x10^-18^. Differences in weighted mean trends regressed on time. $\mu_{t,temp}^{*}-\mu_{t,tropics}^{*}=\alpha +\beta \times year$ is estimated with ordinary least squares (OLS). We give est. *β* (stnd. err.) for each regression. **(A)** –6.6x10^-5^ * (3.7x10^-5^); **(B)** –8.1x10^-5^ (7.1x10^-5^); **(C)** 0.47 *** (0.04); **(D)** –0.14 (0.10); **(E)** –0.0006*** (8.1x10^-5^); **(F)** –0.0004 *** (0.0001). Weighted mean trends regressed on time. $\mu_{t,r}^{*}=\alpha +\beta \times year$ is estimated with OLS. We give est. *β* (stnd. err.) for each regression. **(A)** tropics: 5.0x10^-5^ (3.5 x10^-5^); temperate: –1.6x10^-5^ (1.3x10^-5^); **(B)** tropics: 0.0003 *** (4.8x10^-5^); temperate: 0.0003 *** (6.4x10^-5^); **(C)** tropics: 0.12 *** (0.02); temperate: 0.59 *** (0.05); **(D)** tropics: 0.99 *** (0.08); temperate: 0.85 *** (0.14); **(E)** tropics: 0.0001 *** (3.1x10^-5^); temperate: –0.0004 *** (6.6x10^-5^); **(F)** tropics: 0.0002 ** (9.2x10^-5^); temperate: –0.0002 *** (6.2x10^-5^). All t-tests are two tail tests. ‘***’ indicates estimated coefficient significance at the p = 0.01 level, ‘**’ indicates estimated coefficient significance at the p = 0.05 level, and ‘*’ indicates estimated coefficient significance at p = 0.10 level.

Between 1992 and 2010, tropical countries produced and consumed a greater phylogenetic diversity of commercial plants (# of plant species in database = 322) than temperate countries (Tropics: # of countries = 70 and Temperate: # of countries = 71 (Fig 1A and 1B; *t*-tests of the difference weighted mean trends across zones, p < 0.01). Because of trade, both zones consumed more phylogenetic diversity than they produced and the tropic-temperate gap in phylogenetic diversity is much smaller in consumption than production (compare Fig 1A to Fig 1B; *t*-tests of differences in weighted mean production and consumption trends for each zone, p < 0.01). However, there is little evidence to suggest that either the phylogenetic diversity production or consumption gap between the two zones is converging over time (see the *t*-test results for differences in weighted mean trends regressed on time in the Fig 1 legend).

Even though the tropical zone has greater phylogenetic diversity of commercial plant production and consumption, temperate countries produced and consumed a greater number (SR) and evenness (E) of plant species than tropical countries (Fig 1C–1F; *t*-tests of the differences in weighted mean trends across zones, p < 0.01). As expected, the more complex economies of the temperate zone have the capacity to produce more items and maintain a more even level of consumption, suggesting that temperate zone countries have the ability to find alternative sources of plants when supply from a usual source is temporarily disrupted due to weather or economic perturbations. Again, trade has allowed these regions to consume richness beyond their richness of production (*t*-tests of differences in weighted mean production and consumption trends for each zone, p < 0.01). The temperate-tropics gap in SR production grew from 1992 to 2010 (*t*-tests of differences in weighted mean trends regressed on time, p < 0.01), whereas the temperate-tropics gap in SR consumption did not.

Production evenness declined slightly over time in the temperate zone and slightly increased over time in the tropical zone (estimated coefficients on year variables in weighted mean trends regressed on time, p < 0.01). This downward trend in production E in the temperate zone suggests greater production specialization between 1992 and 2010, at least in that part of the world. Finally, produced E was greater than consumed E in both regions. In other words, between 1992 and 2010, consumers in a country concentrated on a fewer species than its producers.

### Are countries increasingly specializing in plant production and diversifying in plant consumption as they become more open to trade?

To test our plant diversity-comparative advantage hypotheses, we built a statistical model that relates annual country-level changes in diversity or richness metrics to annual country-level changes in openness to trade and per capita income (see Materials and Methods for details in the statistical model). In the model changes in trade openness and income are interacted with latitude. From these interactions we can determine how the functional relationship between changes in diversity and richness metrics and annual changes in trade openness and income varied across the latitude gradient.

#### Explaining production diversity and richness metrics

Changes in the phylogenetic diversity and richness production metrics were rarely explained by contemporaneous changes in the country-level trade openness and per capita income (Table 2 and S1 Fig). Of the three production diversity and richness metrics (PSV, SR, and E), all of which we expected to be negatively associated with gains in trade openness, only change in production E had a statistically significant negative relationship with change in trade openness (p < 0.1). Further, our expectation that production SR and PSV expanded in countries as they became richer, all else equal, is not supported by the data; in fact, production PSV and SR fell as per capita income increased (but not in a statistically significant manner). We do not get different results if we use production diversity and richness metrics that only include food species (Table 2). Finally, our production results are robust to re-estimating the statistical model with lagged changes in trade openness and per capita income. For instance, if a change in production SR is measured from 2000 to 2001 and the model includes both 1999 to 2000 and 2000 to 2001 changes in trade openness and per capita income then our statistical model includes a one-year time lag. We used trade openness and income lags in our model to consider the possibility that agricultural production decisions could partly be explained by macroeconomic changes that took place a year or two prior (see S1 and S2 Tables for model estimates with one and two years of lagged independent variables, respectively).

**Table 2:** Estimates of the relationships between annual country-level changes in diversity and richness metrics and contemporaneous country-level changes in trade openness and per capita income.

	**All Plants**				**Food Plants**			
	***g***	***g* x |*L*|**	***o***	***o* x |*L*|**	***g***	***g* x |*L*|**	***o***	***o* x |*L*|**
	**Production**							
**PSV**	-0.41	0.04	-0.38	0.01	-1.22	0.06	-0.52	0.01
	(2.74)	(0.09)	(1.12)	(0.04)	(2.85)	(0.10)	(1.16)	(0.04)
**SR**	-9.58	0.05	3.66	-0.02	-19.48	0.66	0.15	0.10
	(14.54)	(0.50)	(5.93)	(0.20)	(14.58)	(0.50)	(5.94)	(0.20)
**E**	-10.51	0.81*	-8.83*	0.18	-2.78	-0.26	-0.38	0.05
	(12.66)	(0.44)	(5.16)	(0.17)	(4.72)	(0.16)	(1.92)	(0.06)
	**Consumption**							
**PSV**	14.17*	-0.88***	0.21	0.09	17.07**	-1.10***	0.14	0.07
	(7.68)	(0.26)	(3.13)	(0.10)	(7.57)	(0.26)	(3.09)	(0.10)
**SR**	155.85**	4.77**	51.36**	-1.69**	110.04*	5.47***	55.9**	-1.78**
	(63.38)	(2.18)	(25.79)	(0.85)	(57.54)	(1.98)	(23.42)	(0.77)
**E**	-30.60	0.86	5.26	-0.45	-59.50***	1.40***	-0.16	0.03
	(29.79)	(1.02)	(12.17)	(0.40)	(14.86)	(0.51)	(6.06)	(0.20)

We present twelve model estimates with dependent variable (SR, PSV, or E) in the first column, relevant dataset (all plants vs. only food plants) in the first row, and independent variables in the remaining columns. *g* indicates contemporaneous annual change in logged country-level gross domestic product per capita, *o* indicates contemporaneous annual change in logged country-level trade openness, and |*L*| is the absolute value of country capital latitudes (N = 141, 18 time periods used in the analysis). The models involving the *Food Plants* use a subset of the main dataset, in which the plant diversity and richness metrics only include plant species with positive kilocalories. The number in each cell gives the model’s estimated coefficients; standard errors are given in parentheses. All coefficient and standard error estimates are multiplied by 1000 for readability. Significance levels:

‘***’ 1%

‘**’ 5%, and

‘*’ 10%.

See Materials and Methods for details on how to interpret estimated coefficients, S2 Fig for a graphical representation of model estimates, S1 and S2 Tables for estimates of the model that include lagged independent variables, and S1 and S2 Files for details on model estimates.

#### Explaining consumption diversity and richness metrics

Relative to the changes in the production diversity and richness metrics, relationships among changes in plant consumption metrics and contemporaneous changes in trade openness and income tended to be statistically significant. On average, consumption SR increased in a country as it became more open to trade and richer. Both of these trends support our *a priori* expectations (Table 1). Further, in accordance with our hypotheses, income gains at higher latitudes increased consumption SR even more than income gains in lower latitudes as greater income at higher latitudes gives these countries greater capacity to consume beyond their climatic limitations. As expected, consumption PSV, holding latitude constant, also increased with income. Contrary to expectations, however, the contemporaneous income effect on consumption PSV was higher in lower latitude countries. In fact, while very high latitude countries imported more plant species as their incomes increased, the phylogenetic diversity of plants they consumed *decreased* as they became richer. Again contrary to expectations, gains in openness at lower latitudes had a greater impact on consumption SR than gains in openness at higher latitudes. However, given our study of zonal trends (Fig 1), which indicated that temperate countries tended to produce more richness than tropical countries, this finding, in retrospect, is not surprising: increasing trade allows tropical countries to gain access to the richness produced in the temperate zone. In addition, our expectation that consumption E would increase in trade openness and income per capita is generally not supported by the data. While we expected more connected and richer countries around the world to reduce their variability in consumption, this result only held for countries at the highest latitudes with respect to their food plant consumption.

Finally, the consumption diversity and richness results are less robust to re-estimating the statistical model with lagged changes in trade openness and per capita income. Most notably, changes in trade openness have little to no relationship with changes in consumption SR when we include once or twice lagged annual changes in income and trade openness in our statistical model (see S1 and S2 Tables).

#### The impact of agriculture policy on diversity and richness metrics

A country’s agricultural policies can attenuate pressures to specialize in production and constrain the ability of a population to consume more diversity and richness. To control for the impact of agricultural policy on the relationship between a country’s decisions on production and consumption diversity and richness and its openness to trade, per capita income, and latitude, we re-estimated our statistical model with two policy variables. The first policy variable measures the per annum change in a country’s nominal rate of assistance (NRA) for its farmers. A country’s NRA indicates how much the country’s government policies increase gross returns to its farmers relative to unsubsidized returns. The second country-level agricultural policy variable measures the per annum change in the country’s trade bias in its agricultural sector. The lower a country’s trade bias index (TBI), the more the country subsidizes farmers that produce import-competing crops versus farmers that produce export-orientated crops [33]. We do not have any a priori expectations regarding the impact of these variables on the diversity and richness metrics. We emphasize that consistent NRA and TBI data are only available for 56 of the 171 countries in our 1992 to 2010 database, with data availability biased towards developed countries and several larger developing countries. (Because of this non-random exclusion of countries from the dataset, we did not make the statistical model with policy variables our default model.) Consequently, the conclusions reached with this analysis are not generally applicable.

When we included the policy variables in our statistical model, the relationship between change in production E and contemporaneous change in per capita income became statistically significant (p < 0.01; Table 3). All else equal, with increases in per capita income, a country produced an increasingly less even basket of plant species, especially near the Equator. Further, gains in trade openness at higher latitudes increased production E (p < 0.05; Table 3). This last result is not consistent with our *a priori* expectations. However, the most significant changes to our results caused by adding the policy variables and limiting our dataset to high performing countries took place on the consumption side. First, change in consumption SR was no longer explained by changes in trade openness and income (except when interacted with latitude) and change in consumption PSV was no longer explained by change in income. Further, heavier subsidization of domestic agriculture (an increase in contemporaneous NRA) and a decrease in contemporaneous TBI–agricultural trade policies became more pro-import substitution–were associated with a more even consumption of plants. Finally, when lagged changes in independent variables were included in the model with policy variables (S3 and S4 Tables), changes in consumption PSV and E had a statistically significant relationship with lagged changes in NRA. Further, in the two-period lagged version of the policy model the relationships between changes in consumption diversity and richness metrics and changes in trade openness were inconsistent. Specifically, changes in consumption PSV was negatively correlated with once lagged change in trade openness but positively correlated with twice lagged change in trade openness and the change in consumption E was negatively correlated with once-lagged change in income but positively correlated with the twice lagged change in income. Further, change in consumption SR was negatively correlated to twice-lagged change in income. In total, estimates of the statistical model that includes policy variables suggest that agricultural policy *does* have an impact on consumption diversity and richness trends in more developed countries (recall the dataset that agricultural policy variables is mostly limited to developed countries). Whether these relationships held in the tropics remains unknown because of data gaps.

**Table 3:** Estimates of the relationships between annual country-level changes in diversity and richness metrics and contemporaneous country-level changes in trade openness, per capita income, and agriculture policy.

	**All Plants**								
	***g***	***g* x |*L*|**	***o***	***o* x |*L*|**	***nra***	***nra* x |*L*|**	***tbi***		***tbi* x |*L*|**
	**Production**								
**PSV**	6.91	-0.15	-1.74	0.09*	0.11	0.01		-0.17	0.003
	(5.97)	(0.16)	(2.15)	(0.06)	(2.13)	(0.04)		(1.08)	(0.018)
**SR**	-69.68*	1.22	4.20	0.10	0.08	-0.01		2.23	-0.037
	(37.02)	(0.97)	(13.30)	(0.35)	(13.19)	(0.27)		(6.66)	(0.111)
**E**	-75.39***	1.66***	-9.23	0.47**	4.43	-0.06		0.71	-0.012
	(22.58)	(0.59)	(8.08)	(0.21)	(8.02)	(0.17)		(4.05)	(0.067)
	**Consumption**								
**PSV**	-4.35	-0.37	-0.70	-0.004	-4.22	0.15		2.54	-0.04
	(15.66)	(0.41)	(5.64)	(0.15)	(5.59)	(0.12)		(2.82)	(0.05)
**SR**	116.00	-4.77**	17.19	-0.80	-31.82	0.76		23.04	-0.38
	(80.37)	(2.11)	(28.88)	(0.76)	(28.65)	(0.59)		(14.48)	(0.24)
**E**	-79.62	2.18	3.79	0.08	41.31**	-0.82**		-21.10**	0.35**
	(54.98)	(1.44)	(19.73)	(0.52)	(19.58)	(0.41)		(9.89)	(0.16)

We present six model estimates over all plant data, with diversity and richness metrics in the first column and independent variables in the remaining columns. *g* indicates contemporaneous annual change in logged country-level gross domestic product per capita, *o* indicates contemporaneous annual change in logged country-level trade openness, *nra* indicates contemporaneous annual change in country-level nominal rate of assistance, *tbi* indicates contemporaneous annual change in trade bias index, and |*L*| is the absolute value of country capital latitudes (N = 56 and 18 time periods used in the estimation). The first number in each cell is the estimated coefficient, with standard error in parentheses. All coefficient and standard estimates are multiplied by 1000 for readability. Significance levels:

‘***’ 1%

‘**’ 5%, and

‘*’ 10%.

See Materials and Methods for instructions on how to interpret estimated coefficients, S3 and S4 Tables for model estimates that include lagged independent variables, and S1 and S2 Files for details on model estimates.

#### Robustness check with random forests

**Fig 2:**
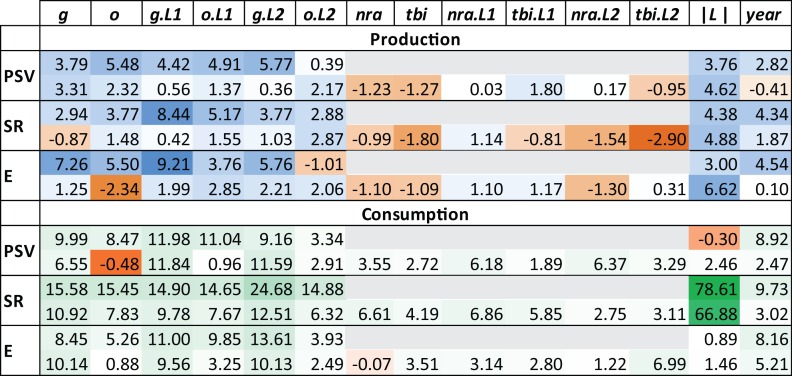
Measures of independent variable importance in explaining changes in production and consumption diversity and richness metrics according to a Random Forest (RF) analysis. Twelve RF estimates of country-level diversity and richness metrics are presented with dependent variables in the first column and independent variables in the remaining columns where *g*, *o*, *nra*, *tbi*, and |*L*| are as before (see the legend to Table 3). These models are estimated over all plant data. *L1* and *L2* indicate that an annual change is one or twice lagged. Each cell indicates the percentage change in mean square error of the modeled fit if the given independent variable is dropped from the diversity or richness model. The darker the color of a cell, the greater the percentage change. Shades of blue or green (orange) indicate a positive (negative) percentage change. The darker the shade of blue (production) or green (consumption), the more important the variable is to model fit. The color scale in the production (consumption) half of the table is normalized against the range of values found in that production (consumption) half of the table. The second row for each metric gives results when *tbi* and *nra* and their lags are included in the RF analysis of a diversity or richness metric model. See models (4) and (5) in Materials and Methods.

Finally, we compared the model results discussed above (based on econometric analysis) to model results generated with random forests (RF) [34]. The econometric model 1) estimates monotonic relationships between changes in a diversity or richness metric and changes in economic variables, 2) assumes changes in trade openness and income do not have an interactive impact on the dependent variable, and 3) assumes the data follows a specific statistical distribution. An RF analysis can approximate functional relationships that are non-monotonic and exhibit complex interactions. Further, RF does not assume the data follows a specific statistical distribution. Therefore, an RF analysis could find support for our hypotheses that the econometric analyses could not–if the relationships in question do not conform to the econometric model’s assumptions. However, consistent with our earlier findings, the RF analysis suggests that, except for consumption SR, changes in production and consumption diversity and richness metrics were not well defined by contemporaneous and lagged changes in trade openness, income, and agriculture policy (Fig 2). While consumption SR is affected by changes in trade openness, income, and policy variables more than the other metrics, its relationship to latitude is particularly striking. Further, the RF analysis confirms our earlier finding that agricultural policy had little effect on production trends but helped shape consumption trends. Finally, the RF analysis suggests that changes in production and consumption metrics are no better explained by lagged change in economic variables than contemporaneous change in economic variables.

## Discussion

Our analysis indicates striking differences in commercial plant production and consumption patterns between the tropical and temperate zones. Tropical countries have a deeper evolutionary heritage and, despite an increasingly globalized world, continue to dominate temperate countries in the phylogenetic diversity of plants produced and consumed. On the other hand, temperate countries have more complex and wealthier economies and have translated this greater technical capacity and stronger preference for variety into the production and consumption of more (albeit, closely related) species than the tropical zone. Gaps in the latitudinal production and consumption of plant diversity and richness have remained relatively static over time despite economic globalization.

### Production diversity and richness

We are not able to explain trends in country-level plant production diversity and richness. Only the evenness of species richness production was related to trade openness in a manner consistent with comparative advantage theory: the bulk of plant production became more concentrated as openness to trade increased (Table 1). However, when we included lagged economic variables in our model or included agricultural policy variables in our model, increases in openness to trade were generally not related to production diversity and richness metrics in expected ways. The only consistent production diversity trend we found was contrary to comparative advantage theory: no matter the dataset and variables used, the phylogenetic diversity of produced plants did not fall, on average, in countries whose farmers faced increasingly more international competition.

Our study period (1992–2010) begins with the fall of the Iron Curtain and the concomitant widespread gains in openness to trade across the globe (S2 Fig). We expected that this would incentivize countries to increasingly specialize in the production of plants best suited to their agro-ecological conditions. Our results indicate that countries largely did not respond as expected. Instead the latitudinal pattern of produced phylogenetic diversity remained relatively static between 1992 and 2010. These results suggest that the rewards to crop specialization in an increasingly connected world may need more than 20 years to take hold or are not as strong as theorized.

In addition to persistent domestic subsidization of agriculture production [35], there are several other phenomena that could make further country-level crop specialization less desirable than predicted. Cultural traditions are still a primary factor in determining the demand for food within a country, whereas culture may be less important for other tradable goods like electronics and clothes [36,37]. A cultural tradition met by locally produced food would seem to go against specialization in export-orientated crops. Although cultural traditions evolve over time, the time scales are longer than the score of years considered in our analysis. Therefore, farmers around the world may still devote substantial effort to satisfying local cultural demands and not the few crops they have a comparative advantage in.

In addition, farmers, especially smaller farmers in the developing world, may be reluctant to switch to export-orientated crop production due to thin and volatile food markets in their home regions; often it is less risky to grow your own food then to rely on spotty markets to consistently provide affordable food [38–40]. Or, farmers willing to become more export-orientated in their crop choices many not be able to make the conversion because of credit or technology transfer constraints. Or in some cases, risk adverse farmers hedge their bets and jointly produce crops that satisfy both local and foreign demand. For example, small-scale farmers in the central Guatemalan highlands have begun to produce crops to fulfill export demand while still growing some crops that are consistent with their and local market diets [41]. Finally, the emerging demand for local food in richer temperate countries has led to the creation of many farms that cater solely to local preferences (e.g., [42,43]). Therefore, globalization, and a growing resistance to some of its consequences, may have helped create two agricultural production sectors in many economies: a specialized export-oriented crop sector that behaves according to comparative advantage theory and the persistence of a more general subsistence or local food sector, all leading to a continued diversity and richness of production [44]. Such a bifurcation is consistent with little change in production PSV but a decrease in production E over time where export-oriented crops increasingly form the bulk of production (Fig 1).

### Consumption diversity and richness

When we analyze all countries in the world, therefore ignoring agricultural policy data, we find that contemporaneous increases in per capita income were associated with the consumption of a richer and more diverse set of plants, as expected [12, 45]. This overall trend did vary across the latitudinal gradient. Compared to trends in lower latitude countries, increases in per capita income were associated with even greater gains in consumed SR and consumed food E in higher latitude countries. Interestingly, greater spending on richness at higher latitudes was also associated with reductions in consumed PSV. In other words, while higher latitude consumers used more of their additional income on species richness than their lower latitude counterparts, the additional richness they bought tended to be evolutionarily similar to the plant products they were already consuming. Finally, consistent with comparative advantage theory, consumed species richness increased (at most latitudes) with openness to trade. However, recall that these relationships between changes in consumed richness and changes in per capita income and trade openness become more tenuous after controlling for contemporaneous changes in agricultural policies or lagged economic variables.

### Implications

All of this means that the absolute gaps in the production and consumption metrics between the typical tropical and temperate country (Fig 1) did not appreciably close between 1992 and 2010 despite increasing opportunities to do so. At least since 1992, tropical countries have produced and consumed more evolutionary diversity (PSV) than temperate countries, and there is no evidence that the gaps in these trends are converging. Conversely, production and consumption of species richness and its evenness (SR and E) were significantly greater in the temperate zone. Of all the tropical–temperate gaps, only the gaps in production and consumption E were converging from 1992 to 2010, albeit slowly (Fig 1). Therefore, the tropics have been the world’s primary source and sink of domesticated plant phylogenetic diversity, and the more complex economies at higher latitudes have been the world’s primary source and sink of domesticated plant richness. The legacy of latitudinal gradients in the diversification of domesticated plants on Earth still dominates production and consumption patterns, despite large increases in globalization and wealth.

Whether the inertia in the latitudinal production of plant phylogenetic diversity is a hindrance or advantage as both climate change accelerates and the pressure to produce even more food for a growing world intensifies, is context dependent. On one hand, less specialized production patterns within countries and regions will make global crop production more redundant and resilient to climatic and social perturbations. On the other hand, global crop production will have to become much more efficient (higher yields per unit area of cropland) to make the transition to a hotter and more crowded world as smooth as possible. Each country specializing in the crops that are best suited for their agro-climate conditions would increase global efficiency in plant production.

## Materials and Methods

### Data

We use commercial plant production, import, and export data from 1992 to 2010 across 142 nations in our analyses of country-level production and consumption diversity and richness trends [46]. While data on commercial plant production, import, and export exist back to 1961 for many countries, we limit our analysis to the 1992 to 2010 timeframe for several reasons. First, data for most Eastern European countries, many developing countries, and countries formed by the dissolution of the Soviet Union are only available from 1992 onwards. Second, production and trade decisions across the world after 1991 were more likely driven by economic incentives and less likely driven by ideology due to the end of the Cold War [47]. For example, many western European countries began to import many more goods from central and eastern European after 1991 as the latter could 1) now trade with western Europe and 2) produce many consumable goods much more cheaply than western European factories due to the lower wages in the East [48].

We estimated the consumption of plant item *j* (N = 169) in country *k* in year *t* in metric tons (Mg), *c*_*jkt*_, with,

$$c_{jkt}=p_{jkt}+i_{jkt}–e_{jkt}$$ (1)

where *p*_*jkt*_ indicates *j*’s production in country *k* in year *t*, *i*_*jkt*_ indicates *j*’s import into country *k* in year *t*, and *e*_*jkt*_ indicates *j*’s export from country *k* in year *t* where all values are measured in Mg. The set of items in FAOStat’s production (*p*) dataset are plants while the set of items in the import (*i*) and export (*e*) dataset include plants and processed foods (e.g., N = 545). Therefore, before calculating *c*_*jkt*_ for each *j*, *k*, and *t* combination we translated all processed food import and export Mg values into their constituent plant Mg using FAOStat conversion rates (see S1 File for more additional materials and methods) [37]. For the results presented in the main text we assume a representative set of conversion rates. We redid our analysis with different sets of conversion rates and conclude that our results are insensitive to the set of conversion weights used (see S3 Fig and S1 File for more details).

Many of the plant items in the FAOStat database are aggregates of multiple species (median: 1 species per plant item, mean: 2.49, max: 21; N = 322). We converted multispecies production (*p*) and consumption (*c*) values into species-specific values in order to create metrics of country-level produced and consumed plant diversity and richness. Due to a lack of information on the consumed and produced distribution of species within each FAO item, we chose to evenly divide each observation of *c*_*jkt*_ and *p*_*jkt*_ across all the species that comprise item *j*. Let *c*_*j*(s)*kt*_ and *p*_*j*(*s*)*kt*_ indicate the Mg consumption and production, respectively, of species *s*, which is a member of plant item *j*, in country *k* in year *t*. For example, the sour cherries plant item is comprised of two species, *Prunus cerasus* and *Cerasus acida*. In 2000, the United States produced 127,640 Mg of sour cherries. We assigned half of this mass to *Prunus cerasus* and the other half to *Cerasus acida* or *p*_*j*(1)*kt*_ = 63,820 and *p*_*j*(2)*kt*_ = 63,820 where *s* = 1 and 2 index the species *Prunus cerasus* and *Cerasus acida*, respectively, *j* = sour cherries, *k* = United States, and *t* = 2000.

The variables *c*_*j*(s)*kt*_ and *p*_*j*(*s*)*kt*_ can alternatively be measured in kilocalories (kcals). To convert Mg to kcals for each *c*_*j*(s)*kt*_ and *p*_*j*(*s*)*kt*_ we used the USDA’s National Nutrient Database for Standard Reference [49] or in a few cases, alternative data sources found through internet searches. If plant item *j* is not a food crop or if species *s* does not contain measurable kcals then *c*_*j*(s)*kt*_ = 0 and *p*_*j*(*s*)*kt*_ = 0 in the food crop data set. Therefore, the food or kcal dataset contains fewer species than the Mg data set.

To measure country-level income, we use gross domestic product per capita (2005 USD) [50]. While GDP does not capture many elements of a country’s wealth, consumption capacity, and distribution of purchasing power in a country, it is the only country-level metric of income that is widely available, both across space and time. To measure the extent a country participates in international trade–its connectedness to the global economy–we use overall trade openness. Trade openness in country *k* in year *t* is equal to the sum of *k*’s exported and imported value in year *t* divided by its total gross domestic product in year *t* [41]. This statistic is commonly used by economists to measure a country’s economic connectedness to the rest of the world [38]. The trade openness statistic considers the openness of the whole economy, not the agricultural sector in particular.

### Metrics of commodity diversity and richness

We calculated three metrics of plant production and consumption specialization for each country *k* in each year *t*. Phylogenetic diversity in production (consumption) in country *k* in year *t* is given by production (consumption) phylogenetic species variability (PSV) in country *k* in year *t*. PSV_*kt*_ measures the average relatedness across the set of plant species produced (consumed) in *k* in year *t*. Higher values of PSV indicate that the set of produced (consumed) crop species are more distantly related to each other (the set is more diverse) [23]. Species richness in country *k* in year *t*, SR_*kt*_, measures the total number of plant species produced (consumed) in *k* in year *t*. The statistical expectation of PSV is independent of SR, meaning that SR and PSV can be independently interpreted. For evenness in the richness of production (consumption) in country *k* in year *t* we use Simpson’s evenness, E_*kt*_ [26]. We used Mg weights (all plant species), or alternatively, kcal weights (food plant species only) when calculating E_*kt*_. Plant species phylogenetic relationships come from Zanne et al. [51]. We added additional species to the phylogeny using *congeneric*.*merge* [22].

To test whether the uniform distribution of each plant item *j*’s production (consumption) Mg across its constituent species biases our diversity metrics of country-level production (consumption), we alternatively calculated *p*_*j*(*s*)*kt*_ (*c*_*j*(*s*)*kt*_) by randomly selecting a subset of each *j*’s species in each *k* over which to evenly divide *k*’s tonnage values for *j*. For example, if plant item *j* has four species *s* then a random species subset assignment could mean that *k*’s production Mg for plant item *j* could be split 50–50 between the 2^nd^ and 3^rd^ species of *j*. In another country the production of *j* could be assigned entirely to the 4^th^ species under this item. We found that the country-level diversity and richness metrics estimated with uniform distribution of Mg across all species in each plant item (the measures we use in this analysis) to be very similar, in a relative sense, to country-level diversity and richness metrics estimated with random assignment of the subset of species for each *j* in each *k*.

See S1 and S2 Files for more on the metrics of commodity diversity and richness, including metric formulas, and S3–S29 Files for data used in our analysis.

### Statistical Analyses

To test whether trends in country-level production and consumption diversity and richness have been associated with changes in a country’s openness to trade and per capita income, and whether these effects vary with latitude, we estimated the following static spatial panel model,

$$y_{kt}=\left(\gamma_{1}+\gamma_{2}\left|L_{k}\right|\right)g_{kt}+\left(\gamma_{3}+\gamma_{4}\left|L_{k}\right|\right)o_{kt}+\omega_{k}+\rho \sum_{j=1}^{K}W_{kj}y_{jt}+\lambda \sum_{j=1}^{K}W_{kj}\varepsilon_{jt}+\mu_{kt}$$ (2)

where *y*_*kt*_ = log(*q*_*kt*_)–log(*q*_*kt-1*_) and *q*_*kt*_ is a measure of one of the three diversity or richness metrics in country *k* at year *t* (*j* also indexes countries). The variable |*L*_*k*_| is the absolute value of *k*’s capital city’s latitude, *g*_*kt*_ = log(*GDP*_*kt*_)–log(*GDP*_*kt-1*_) and *o*_*kt*_ = log(*Open*_*kt*_)–log(*Open*_*kt-1*_) where *GDP*_*kt*_ is gross domestic product per capita in country *k* in year *t*, and *Open*_*kt*_ is trade openness in country *k* in year *t*. Further, the variable *ω*_*k*_ is a fixed effect that factors out the mean overall differences among countries unrelated to the other covariates. For example, some countries may have a culture or a relatively wide range of climates that encourages growth in the diversity of food consumption independent of changes in wealth and exposure to the global economy. Finally, **W** is an inverse distance matrix where country pair-wise distances (*i*,*j*) are measured from capital to capital, *ε*_*jt*_ and μ_*kt*_ are error terms, and **γ**, *ρ*, and *λ* are coefficients to be estimated.

A static model includes contemporaneous causal factors but omits prior changes in the causal factors (e.g., when change in the diversity metric is measured from 2000 to 2001 a prior causal factor is given by 1999 to 2000 change in trade openness). If these previous iterations of *g*_*k*_ and *o*_*k*_ contribute to the realization of *y*_*k*_ then model (2)’s estimates will be biased. Therefore, we also estimated distributed lag forms of model (2), either with one or two previous iterations of *g*_*k*_ and *o*_*k*_−interacted with latitude–as explanatory variables as well. We experiment with lagged forms of economic variables because farmers and consumers may continue to react to changes in country-level income and trade openness several years after the change.

When non-stationary time trends are used in econometric analysis estimated coefficients are undefined. Here we assume that the time series we use–the diversity metrics, GDP per capita, etc.–display a random walk, a non-stationary process. Under such an assumption, taking year to year differences in the dependent and independent variables makes the data stationary, and therefore, means model coefficients can be statistically defined. This explains why our statistical models use annual *change* variables instead of annual levels.

For each diversity and richness metric, we estimated model (2) six times: three times with a spatial auto-regressive (SAR) form and three times with a spatial error model (SEM) form. Each three-estimate block includes a static model estimate (model (2) as is) and estimates of model (2) with once or twice lagged economic variables. We used spatial regression techniques to control for the impact of omitted explanatory variables that are correlated across space. For example, the Euclidean distance between countries controls for 1) countries’ tendency to trade most heavily with their immediate neighbors and 2) for any unobservable effects that might jointly affect the diversity production and consumption choices in a block of countries (e.g., a hurricane affects fruit production throughout Central America in year *t*). The SAR accounts for spatial externalities by regressing country *j*’s dependent variable value on all other countries’ dependent variable values. The SEM accounts for omitted spatial correlations by adjusting the error term. In the SAR estimate of model (2), *λ* is set to 0, and ρ quantifies the impact of (*y*_*1t*_, …, *y*_*k-1t*_, *y*_*k+1t*_,…, *y*_*Kt*_), as mediated by distance, on *y*_*kt*_. In the SEM estimate of model (2), *ρ* is set to 0, *λ* controls for the impact of other country errors in year *t* on *y*_*kt*_, and μ_*kt*_ is country *k*’s error term in year *t*.

SAR estimates can be decomposed into direct and indirect effects [52]. When model (2) only includes contemporaneous *g*_*kt*_ and *o*_*kt*_ the direct coefficients indicate the impact of small changes in *k*’s per capita income and openness on contemporaneous changes in *k*’s dependent variable, accounting for these impacts passing through *k*’s neighboring countries and back to the *k* itself. In contrast, the indirect coefficients measure the impact of neighboring country changes in *g*_*kt*_ and *o*_*kt*_ on contemporaneous changes in *k*’s dependent variable. When we use the distributed lag versions of model (2) then the direct coefficients measure the impact of contemporaneous and previous small annual changes in *k*’s per capita income and openness on contemporaneous changes in *k*’s dependent variable, accounting for these impacts passing through *k*’s neighboring countries and back to the *k* itself. Finally, the indirect coefficients in the distributed lag versions of model (2) measure the impact of neighboring country contemporaneous and previous changes in *g* and *o* on contemporaneous changes in *k*’s dependent variable. (SEM regression coefficients indicate the full impact of small changes in the independent variables on the dependent variable; in other words, they can be interpreted the same way least squares regression coefficients are.)

In Tables 2 and 3 and S1–S4 Tables we only report the *direct* SAR estimates. While the indirect impacts on *k*’s diversity and richness metrics are interesting, they are not the focus of this study. SEM estimates are very similar to SAR estimates and therefore are not reported.

By log-transforming all variables, estimated model (2) coefficients represent elasticity measures. For example, consider the estimate of the static all plant consumption SR model (Table 2). Suppose |*L*| = 10. In this case a 1% annual increase in a country’s GDP per capita is associated with a 0.2% contemporaneous change in the country’s all plant consumption SR, all else equal (i.e., 1.01^((155.85/1000) + (47.7/1000))^ = 1.002). Elasticity estimation is a bit more complicated when we include lagged independent variables in model (2). The steady-state or long-run diversity or richness effect of a permanent 1% annual change in an economic variable is calculated by combining the effects of the contemporaneous and lagged changes. For example, at |*L*| = 0, a permanent 1% annual increase in a country’s GDP per capita is associated with a $1.01^{\left((\gamma_{1}/1000)+(\gamma_{2}/1000)+(\gamma_{3}/1000)\right)}$ long-run annual change in the relevant diversity or richness metric when using the twice lagged version of model (2) (see S2 Table for estimated coefficient values).

Further, magnitudes of elasticity measures are directly comparable. For example, at |*L*| = 0, the estimated relationship between a 1% annual increase in GDP per capita and a contemporaneous percentage change in all plant production SR is 23.4 times stronger than the relationship between a 1% annual increase in GDP and a contemporaneous change in all plant production PSV. We can conclude this because estimated *γ*_1_ in the all plant production SR model is 23.4 greater than the estimated *γ*_1_ in the all plant production PSV model (i.e., -9.58 / -0.41 = 23.4; see Table 2).

We conducted several other analyses to support the conclusions we derive from our analysis of estimated model (2). First, we tested for whether country-level agricultural policy could have also affected diversity and richness trends in plant production and consumption. To do this we add annual changes in country-level nominal rate of assistance (NRA) and trade bias index (TBI)–metrics of the impacts of government interventions and policy developments on farmers’ incentives [30]–to model (2). NRA indexes the rate of agricultural subsidization in a country and TBI indexes the extent to which a country subsidizes import-competing crops versus export-orientated crops.

$$y_{kt}=\left(\gamma_{1}+\gamma_{2}\left|L_{k}\right|\right)g_{kt}+\left(\gamma_{3}+\gamma_{4}\left|L_{k}\right|\right)o_{kt}+\left(\gamma_{5}+\gamma_{6}\left|L_{k}\right|\right)tbi_{kt}+\left(\gamma_{7}+\gamma_{8}\left|L_{k}\right|\right)nra_{kt}+\omega_{k}+\rho \sum_{j=1}^{K}W_{kj}y_{jt}+\lambda \sum_{j=1}^{K}W_{kj}\varepsilon_{jt}+\mu_{kt}$$ (3)

where *tbi*_*kt*_ = *TBI*_*kt*_*−TBI*_*kt-1*_ and *nra*_*kt*_ = *NRA*_*kt*_−*NRA*_*kt-1*_ and all other variables are as before. We do not immediately include NRA and TBI in model (2) because consistent data on these two series for 1992 to 2010 are only available for 56 of the 171 countries in our 1992 to 2010 database. The 56 countries are mostly Organisation for Economic Co-operation and Development countries and a few larger developing countries. We do not log these NRA and TBI before taking the first difference because NRA and TBI takes on values less than 0 (NRA ranges from -0.46 to 3.10 and TBI ranges from -0.8 to 1.12). In Table 3 we present the *direct* SAR estimates of the static version of model (3). In S3 and S4 Tables we give the *direct* SAR estimates of two distributed lag forms of model (3)

To quantify the impact of small changes in NRA and TBI on the annual change in a diversity or richness metric we use the exponential. For example, consider the estimate of the *static* all plant consumption E model (Table 3). Suppose |*L*| = 10. In this case a 1 unit increase in a country’s *nra* value is associated with a 3.4% contemporaneous annual change in the country’s all plant consumption E, all else equal (i.e., exp((41.31/1000)–(8.2/1000)) = 1.034). The coefficients on *g* and *o* in model (3) are interpreted as they were in model (2). Further, the impacts of a permanent annual change in an economic variable when policy variables are included in our model, which is given by the lagged form of model (3), are calculated in the same fashion as permanent impacts are derived from the estimates of model (2). See S27–S29 Files for the datasets used to estimate models (2) and (3).

Further, we analyze versions of models (2) and (3) with Random Forests (RF) to learn more about the relationship between our dependent and explanatory variables.

$$y_{kt}=\sum_{l=0}^{2}\beta_{l}g_{kt-l}+\sum_{l=0}^{2}\beta_{l+3}o_{kt-l}+\beta_{6}\left|L_{k}\right|+\beta_{7}year$$ (4)

$$y_{kt}=\sum_{l=0}^{2}\beta_{l}g_{kt-l}+\sum_{l=0}^{2}\beta_{l+3}o_{kt-l}+\sum_{l=0}^{2}\beta_{l+6}nra_{kt-l}+\sum_{l=0}^{2}\beta_{l+9}tbi_{kt-l}+\beta_{12}\left|L_{k}\right|+\beta_{13}year$$ (5)

RF is a robust machine learning algorithm capable of modeling non-linear and non-monotonic dependencies while avoiding overfitting [34]. An RF analysis ranks the importance of each explanatory variable in overall model fit by calculating the percentage change in mean square error of the modeled fit when an independent variable is dropped from the model. For example, a 3.79% for variable *g*_*kt*_ in model (4) when *y*_*kt*_ is production PSV means the mean square error of the model that estimates the relationship between production PSV and the economic and policy variables increases by 3.79% when GDP per capita is dropped from the model. Variables that generate larger percentage gains in mean square error of the modeled fit help explain a larger portion of model fit. In RF models (4) and (5) we include time as an explanatory variable; it is not included in model (2). We assume that time in and of itself is not important in explaining trends in *y*_*kt*_. We can test this assumption with the RF analysis of models (4) and (5).

## Supporting Information

S1 Fig Model (2)’s contemporaneous marginal effects at various latitudes for all plants. The graphed contemporaneous income per capita marginal effects are equal to estimated γ_1+_γ_2_|L| and contemporaneous trade openness marginal effects are equal to estimated γ_3+_γ_4_|L| for |*L*| = 5, 25, 45, and 65 degrees of latitude. All marginal effects are multiplied by 1,000 for readability. See Table 2 for all estimated coefficient values. We use thumbnail graphs at the top of the figure to indicate the expected marginal effect sign and magnitude change across the latitude gradient for each dependent–independent variable combination. The dashed lines indicate the 5^th^ and 95^th^ confidence interval of the estimated marginal effect.
(TIF)

S2 Fig Percentage changes in trade openness and real GDP per capita by country between 1992–1994 and 2008–2010. The initial data point for each country (N = 141) is given by their 1992–1994 trade openness and real GDP per capita annual averages. The terminal data point for each country is given by their 2008–2010 trade openness and real GDP per capita annual averages. Temperate countries are black and tropical countries are red.
(TIF)

S3 Fig Sensitivity analysis of weighted mean trends in zonal consumption diversity and richness. When we calculated *c*_*jkt*_ for each *j*, *k*, and *t* combination, as measured by Mg, we had to translate all processed food import and export Mg values into their constituent crop Mg using FAOStat conversion rates. For the results presented in the main text we assume a representative set of conversion rates. Here we show alternative weighted zonal means of the diversity and richness consumption metrics generated with the 10 alternative sets of *c*_*jkt*_ for each *j*, *k*, and *t* combination. In each alternative set conversion rates were randomly selected from a set of potential conversion rates. **(A)**–**(F)** includes the relevant consumption trend line from Fig 1 of the text (blue and green) and its 10 alternative consumption trends lines formed with the alternative sets of *c*_*jkt*_. **(A)** is consumed PSV in the temperate zone. **(B)** is consumed PSV in the tropics. **(C)** is consumed SR in the temperate zone. **(D)** is consumed SR in the tropics. **(E)** is consumed E in the temperate zone. **(F)** is consumed E in the tropics. These graphs indicate that our results are insensitive to the set of conversion weights used.
(TIF)

S1 File Additional Materials and Methods. (DOCX)

S2 File Estimates of models (2) and (3). (DOCX)

S3 File The p and c vectors used in the text. (XLSX)

S4 File One of the 10 alternative c vectors (consumption PSV, SR, and E derived from these data are graphed in S3 Fig). (XLSX)

S5 File One of the 10 alternative c vectors (consumption PSV, SR, and E derived from these data are graphed in S3 Fig). (XLSX)

S6 File One of the 10 alternative c vectors (consumption PSV, SR, and E derived from these data are graphed in S3 Fig). (XLSX)

S7 File One of the 10 alternative c vectors (consumption PSV, SR, and E derived from these data are graphed in S3 Fig). (XLSX)

S8 File One of the 10 alternative c vectors (consumption PSV, SR, and E derived from these data are graphed in S3 Fig). (XLSX)

S9 File One of the 10 alternative c vectors (consumption PSV, SR, and E derived from these data are graphed in S3 Fig). (XLSX)

S10 File One of the 10 alternative c vectors (consumption PSV, SR, and E derived from these data are graphed in S3 Fig). (XLSX)

S11 File One of the 10 alternative c vectors (consumption PSV, SR, and E derived from these data are graphed in S3 Fig). (XLSX)

S12 File One of the 10 alternative c vectors (consumption PSV, SR, and E derived from these data are graphed in S3 Fig). (XLSX)

S13 File One of the 10 alternative c vectors (consumption PSV, SR, and E derived from these data are graphed in S3 Fig). (XLSX)

S14 File Country-level PSV, SR, and E data used in the text (all commodity plants). (XLSX)

S15 File Country-level PSV, SR, and E data used in the text (food plants only). (XLSX)

S16 File Country-level consumption PSV, SR, and E data generated with one of the 10 alternative c vectors. (XLSX)

S17 File Country-level consumption PSV, SR, and E data generated with one of the 10 alternative c vectors. (XLSX)

S18 File Country-level consumption PSV, SR, and E data generated with one of the 10 alternative c vectors. (XLSX)

S19 File Country-level consumption PSV, SR, and E data generated with one of the 10 alternative c vectors. (XLSX)

S20 File Country-level consumption PSV, SR, and E data generated with one of the 10 alternative c vectors. (XLSX)

S21 File Country-level consumption PSV, SR, and E data generated with one of the 10 alternative c vectors. (XLSX)

S22 File Country-level consumption PSV, SR, and E data generated with one of the 10 alternative c vectors. (XLSX)

S23 File Country-level consumption PSV, SR, and E data generated with one of the 10 alternative c vectors. (XLSX)

S24 File Country-level consumption PSV, SR, and E data generated with one of the 10 alternative c vectors. (XLSX)

S25 File Country-level consumption PSV, SR, and E data generated with one of the 10 alternative c vectors. (XLSX)

S26 File Country-level arable land and population data. (XLSX)

S27 File Dataset used to estimate model (2) (all plants). (XLSX)

S28 File Dataset used to estimate model (2) (food plants only). (XLSX)

S29 File Dataset used to estimate model (3) (all plants). (XLSX)

S1 Table Estimates of model (2) with contemporaneous and once lagged changes in independent variables. We present six estimates of model (2) with dependent variables (defined across all plants) in the first column and contemporaneous change and once lagged (*L*.1) independent variables in the remaining columns where *g* indicates annual change in logged country-level gross domestic product per capita, *o* indicates annual change in logged country-level trade openness, and |*L*| is the absolute value of country capital latitudes (N = 141 and 17 time steps). The first (second) number in each cell gives the estimated coefficient (standard error) times 1000. All coefficient and standard estimates are multiplied by 1000 for readability. Sign and significance data are from the *direct* SAR estimates of model (2). See Materials and Methods for instructions on interpreting estimated coefficients. Significance levels: ‘***’ 1%, ‘**’ 5%, and ‘*’ 10%. See S1 and S2 Files for model (2) estimate details and S27 File for data used to estimate model (2).
(DOCX)

S2 Table Estimates of model (2) with contemporaneous and once and twice lagged changes in independent variables. We present six estimates of model (2) with dependent variables (defined across all plants) in the first column and contemporaneous change and once and twice lagged (*L*.1 and *L*.2, respectively) independent variables in the remaining columns where *g* indicates annual change in logged country-level gross domestic product per capita, *o* indicates annual change in logged country-level trade openness, and |*L*| is the absolute value of country capital latitudes (N = 141 and 16 time steps). The first (second) number in each cell gives the estimated coefficient (standard error) times 1000. All coefficient and standard estimates are multiplied by 1000 for readability. Sign and significance data are from the *direct* SAR estimates of model (2). See Materials and Methods for instructions on interpreting estimated coefficients. Significance levels: ‘***’ 1%, ‘**’ 5%, and ‘*’ 10%. See S1 and S2 Files for model (2) estimate details and S27 File for data used to estimate model (2).
(DOCX)

S3 Table Estimates of model (3) with contemporaneous and once lagged changes in independent variables. We present six estimates of model (3) with dependent variables (defined across all plants) in the first column and contemporaneous change and once lagged (*L*.1) independent variables in the remaining columns where *g* indicates annual change in logged country-level gross domestic product per capita, *o* indicates annual change in logged country-level trade openness, *nra* indicates annual change in country-level nominal rate of assistance, *tbi* indicates annual change in trade bias index, and |*L*| is the absolute value of country capital latitudes (N = 56 and 17 time steps). The first (second) number in each cell gives the estimated coefficient (standard error) times 1000. All coefficient and standard estimates are multiplied by 1000 for readability. Sign and significance data are from the *direct* SAR estimates of model (3). See Materials and Methods for instructions on interpreting estimated coefficients. Significance levels: ‘***’ 1%, ‘**’ 5%, and ‘*’ 10%. See S1 and S2 Files for model (3) estimate details and S29 File for data used to estimate model (3).
(DOCX)

S4 Table Estimates of model (3) with contemporaneous and once and twice lagged changes in independent variables. We present six estimates of model (3) with dependent variables (defined across all plants) in the first column and contemporaneous change and once and twice lagged (*L*.1 and *L*.2) independent variables in the remaining columns where *g* indicates annual change in logged country-level gross domestic product per capita, *o* indicates annual change in logged country-level trade openness, *nra* indicates annual change in country-level nominal rate of assistance, *tbi* indicates annual change in trade bias index, and |*L*| is the absolute value of country capital latitudes (N = 56 and 16 time steps). The first (second) number in each cell gives the estimated coefficient (standard error) times 1000. All coefficient and standard estimates are multiplied by 1000 for readability. Sign and significance data are from the *direct* SAR estimates of model (3). See Materials and Methods for instructions on interpreting estimated coefficients. Significance levels: ‘***’ 1%, ‘**’ 5%, and ‘*’ 10%. See S1 and S2 Files for model (3) estimate details and S29 File for data used to estimate model (3).
(DOCX)
