# Supplementary figures and images for: Commercial Plant Production and Consumption Still Follow the Latitudinal Gradient in Species Diversity despite Economic Globalization
Source: PLoS One. 2016 Oct 5;11(10):e0163002. doi: 10.1371/journal.pone.0163002 (PMC5051709; doi:10.1371/journal.pone.0163002)

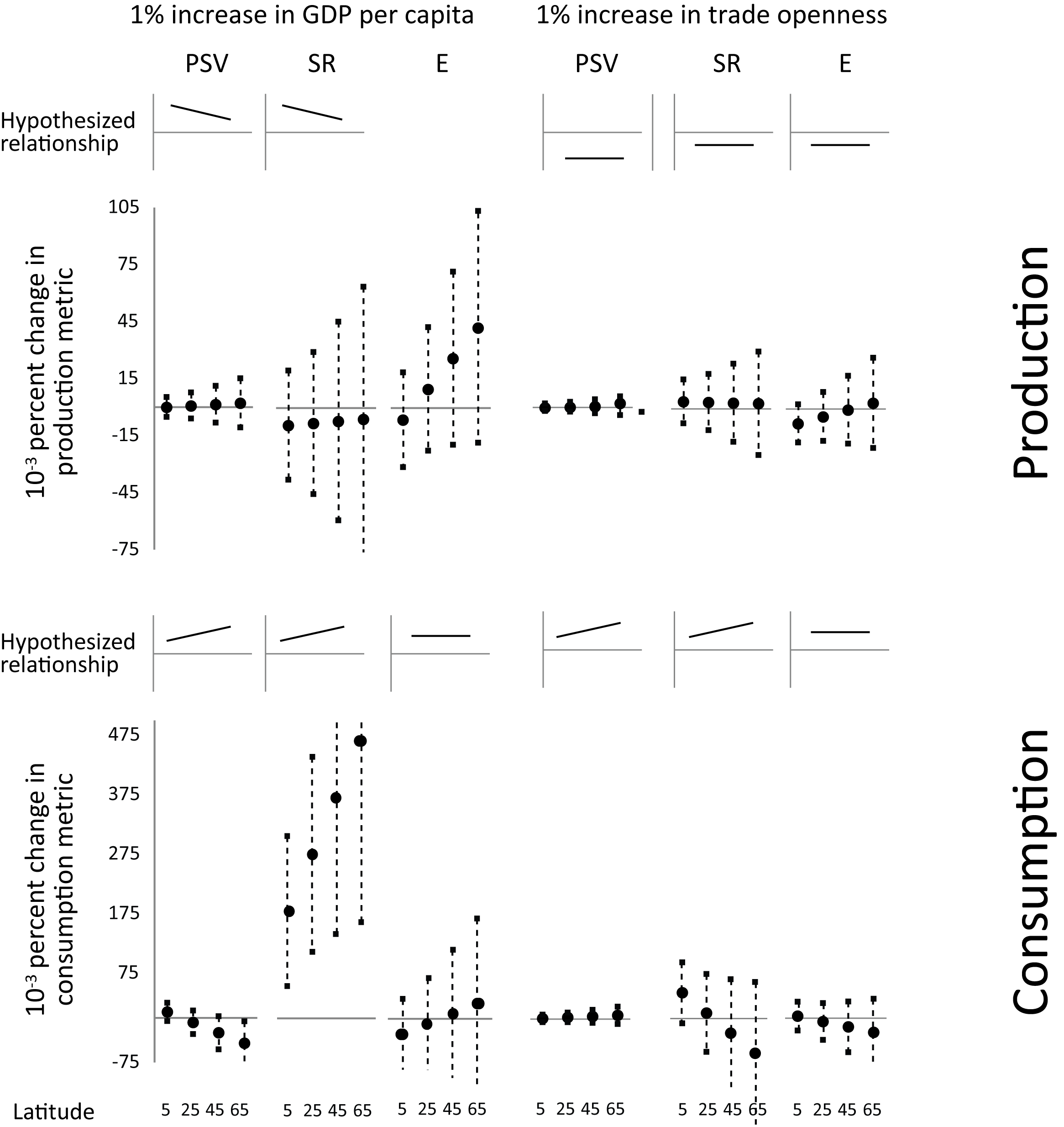

Supplement: S1 Fig — The graphed contemporaneous income per capita marginal effects are equal to estimated γ1+γ2|L| and contemporaneous trade openness marginal effects are equal to estimated γ3+γ4|L| for |L| = 5, 25, 45, and 65 degrees of latitude. All marginal effects are multiplied by 1,000 for readability. See Table 2 for all estimated coefficient values. We use thumbnail graphs at the top of the figure to indicate the expected marginal effect sign and magnitude change across the latitude gradient for each dependent–independent variable combination. The dashed lines indicate the 5th and 95th confidence interval of the estimated marginal effect. (TIF) [file pone.0163002.s001.tif]

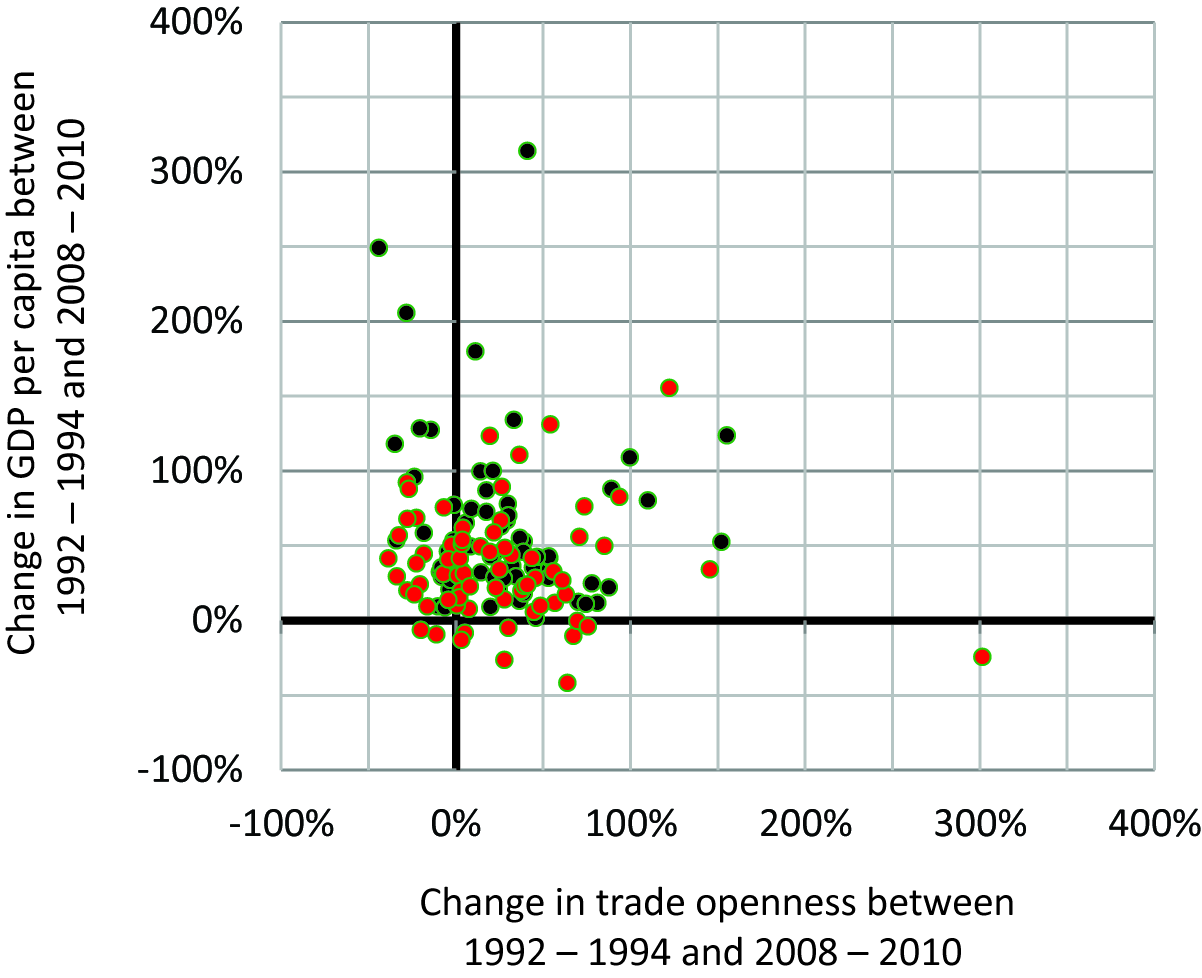

Supplement: S2 Fig — The initial data point for each country (N = 141) is given by their 1992–1994 trade openness and real GDP per capita annual averages. The terminal data point for each country is given by their 2008–2010 trade openness and real GDP per capita annual averages. Temperate countries are black and tropical countries are red. (TIF) [file pone.0163002.s002.tif]

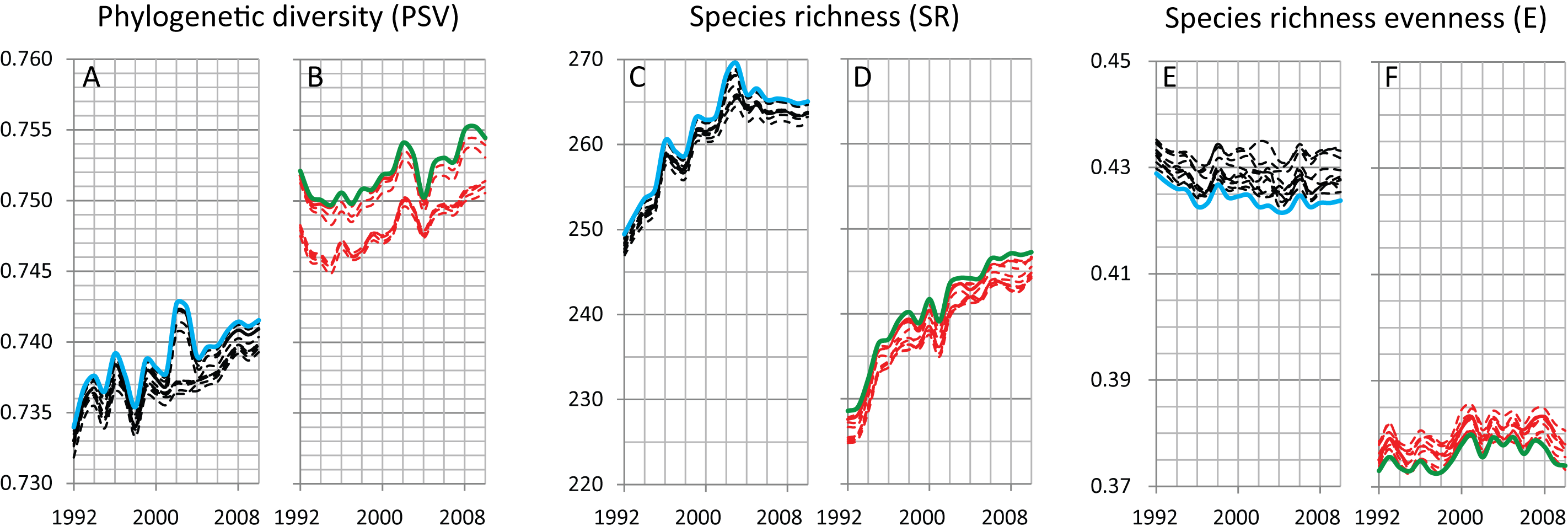

Supplement: S3 Fig — When we calculated cjkt for each j, k, and t combination, as measured by Mg, we had to translate all processed food import and export Mg values into their constituent crop Mg using FAOStat conversion rates. For the results presented in the main text we assume a representative set of conversion rates. Here we show alternative weighted zonal means of the diversity and richness consumption metrics generated with the 10 alternative sets of cjkt for each j, k, and t combination. In each alternative set conversion rates were randomly selected from a set of potential conversion rates. (A)–(F) includes the relevant consumption trend line from Fig 1 of the text (blue and green) and its 10 alternative consumption trends lines formed with the alternative sets of cjkt. (A) is consumed PSV in the temperate zone. (B) is consumed PSV in the tropics. (C) is consumed SR in the temperate zone. (D) is consumed SR in the tropics. (E) is consumed E in the temperate zone. (F) is consumed E in the tropics. These graphs indicate that our results are insensitive to the set of conversion weights used. (TIF) [file pone.0163002.s003.tif]
