# Supplementary material for: Commercial Plant Production and Consumption Still Follow the Latitudinal Gradient in Species Diversity despite Economic Globalization
Source: PLoS One. 2016 Oct 5;11(10):e0163002. doi: 10.1371/journal.pone.0163002 (PMC5051709; doi:10.1371/journal.pone.0163002)
Supplement: S1 File — (DOCX) [file pone.0163002.s004.docx]

Supplementary Materials and Methods

**Creation of country-level crop production and consumption data**

The tonnage of crop *j* = 1,…,169 produced in country *k* in year *t*, given by *p_jkt_*, comes from [46]. The tonnage of food/crop/animal product *f* = 1,….,545 imported to country *k* in year *t*, given by *i_fkt_*, comes from [46]. The tonnage of food/crop/animal product *f* = 1,….,545 exported by country *k* in year *t*, given by *e_fkt_*, comes from [46] as well.

To convert *i_fkt_* and *e_fkt_* to import and export values expressed in imports and exports of crop *j*, in other words, *i_jkt_* and *e_jkt_*, we use published conversion rates between foods/crops and their feedstock crops. (In the cases where *f* is identical to a *j*, e.g., the food /crop imported is unprocessed maize, then there is only one conversion rate, one to one. In the cases where *f* is a meat, dairy, or animal product we do not match it with any *j*; those *f* are dropped from the analysis.) First, for each food/crop *f* we identify all of the possible *j* feedstocks. For example, the food/crop category breakfast cereals can be made, according to FAO, from the crops rice or wheat. Then for each feedstock *j* that can be used to produce food/crop *f* we will have one or more conversion rates. Let *θ_f_* contain every found conversion rate from *f* to *j* = 1,…,*J*. For example, if breakfast cereals can be made from rice or wheat and there are two conversion rates for each feedstock then *θ_f_* has four conversion rates. Each rate measures how many kilograms of crop *j* are needed to create 1 kilogram of *f***.** For example, in some cases 1.250 kg of wheat is needed to make 1 kg of wheat-based breakfast cereal. In other cases, 1.111 kg of wheat is needed to make 1 kg of breakfast cereal. Similarly, 1.493 or 1.658 kg of rice is needed to make 1 kg of rice-based cereal. Therefore, *θ_f_* = [1.250 1.111 1.493 1.658].

For each vector *θ_f_* there is an accompanying import weight vector **w***_fi_* of the same dimension of *θ_f_* where the elements of **w***_fi_* sum to 1. We can either use equally balanced **w***_fi_* vectors (i.e., all elements of **w***_f_* have the same value) or randomly assign values to each element of **w***_fi_* with the only constraint on weight vector construction being that the sum of weight values must be one. Let *w_fl_* x *θ_f_* indicate an element by element multiplication of **w***_fi_* and $\theta_{f}$. Therefore, multiplying each element of *w_fl_* x *θ_f_* by *i_fkt_* converts the mass of *f* imported into *k* in year *t* into masses of feedstock crops *j* imported into *k* in year *t*. For example, returning to our breakfast cereal case, if **w***_fi_* = [0.25 0.25 0.25 0.25] then *w_fl_* x *θ_f_* is equal to [0.3125 0.2778 0.3733 0.4145]. Suppose country *k* imports 100 tons of breakfast cereals in year *t* (e.g., *i_fkt_* = 100). Therefore, when we multiply *i_fkt_* by each of the elements in *w_fl_* x *θ_f_* we get [31.25 27.28 37.33 41.45]. In other words, by exporting 100 tons of breakfast cereals country *k* has really exported 31.25 + 27.28 = 59.53 tons of wheat and 37.33 + 41.45 = 78.78 tons of rice. Note that all countries use the same set of **w***_fi_* and that we assume that conversion rates have not changed between 1961 and 2010. (Obviously the *costs* of conversion have changed due to technological development but the amount of food that can be derived from a kg of feedstock in many cases will not have changed. For example, the amount of Rice Krispy’s that can be derived from a kg of rice has not likely changed).

Similarly we generate an export weight vector **w***_fe_* for each vector *θ_f_*. We can initially set **w***_fe_* = **w***_fi_* or generate different weights for **w***_fi_*. However, no matter how we set **w***_fe_* it may have to be corrected to match production in a country. For example, in the breakfast cereals case above, suppose country *k* did not produce any rice. Therefore, the only feedstock that can contribute to cereal export from k is wheat and therefore, the last two elements of **w***_fe_* have to be equal to 0. We use **w***_fek_* to indicate that an export vector has been corrected to prevent any non-existent feedstocks from being exported from *k*. Finally, multiplying each element of *w_fl_* x *θ_f_* by *e_fkt_* converts the mass of *f* exported from *k* in year *t* into masses of feedstock crops *j* exported from *k* in year *t*.

Once we have done such conversions for all imported and exported *f* = 1,….,*F* and summed appropriately across the same *j* in *k* in year *t* we have preliminary estimates of *i_jkt_* and *e_jkt_* for each *j* = 1,…,*J* in each *k* = 1,…,*K* and *t* = 1,…,*T*. However, before making these values final, we have to perform two more consistency checks. First, we have to verify that *e_jkt_* ≤ *p_jkt_* and if not, than set derived *e_jkt_* equal to *p_jkt_* (a country cannot export more than it produces). Finally, after correcting the cases where *e_jkt_* > *p_jkt_* we have to verify that global exports of product *j* at time *t* are equal to global imports of product *j* at time *t*, and if not, correct the imbalance. If $\sum_{k=1}^{K} e_{jkt}>\sum_{k=1}^{K} i_{jkt}$ for *j* at time *t* we then multiply all instances of *e_jkt_* by $\sum_{k=1}^{K} i_{jkt}/\sum_{k=1}^{K} e_{jkt}$ . Otherwise, if $\sum_{k=1}^{K} e_{jkt}<\sum_{k=1}^{K} i_{jkt}$ for *j* at time *t* we then multiply all instances of *i_jkt_* by $\sum_{k=1}^{K} e_{jkt}/\sum_{k=1}^{K} i_{jkt}$.

Let **p**, **i**, **e**, and **c** be the vectors that contain every observation of *p_jkt_*, *i_jkt_*, *e_jkt_*, and *p_jkt_* + *i_jkt_* – *e_jkt_* across all *j*, *k*, and *t* given a set of **w***_fi_* and **w***_fek_* vectors. As mentioned in the text, we convert the vectors **p** and **c** into vectors of species-level Mg (given by **p_s_** and **c_s_**, respectively, and containing elements *p_j_*(*_s_*)*_kt_* and *c_j_*_(s)_*_kt_*, respectively) and then use them to find the vectors of production and consumption diversity metrics PSV*_kt_*, SR*_kt_*, and E*_kt_* (see below).

The vectors **i**, **e**, and **c** are sensitive to the weights used in the **w***_fi_* and **w***_fek_* vectors. To test how sensitive our estimates of consumption PSV*_kt_*, SR*_kt_*, and E*_kt_* are to the weights used in **w***_fi_* and **w***_fek_* we create 11 alternative sets of **i**, **e**, and **c** (**p** is not sensitive to **w***_fi_* or **w***_fek_* and therefore is calculated just once and the same p vector is used with all 11 sets of **i**, **e**, and **c**)**.** When creating the first set of **i**, **e**, and **c** we use balanced **w***_fi_* and **w***_fek_* for all *f* (unless **w***_fek_* has been corrected to prevent the exportation of non-existent feedstock; in those cases all possible conversions have an equal positive weight and all impossible conversions have a weight of 0). For the 10 other sets of **i**, **e**, and **c** weights in **w***_fi_* and **w***_fek_* are randomly chosen with weights of 0 being possible. Therefore, in the end we have one **p_s_** vector and 11 **c_s_** vectors. In the text we present the consumption PSV*_kt_*, SR*_kt_*, and E*_kt_* vectors that are derived from the **c_s_** using balanced **w***_fi_* and **w***_fek_* for all *f*. In Supplementary Information Figure 3 we summarize the consumption PSV*_kt_*, SR*_kt_*, and E*_kt_* vectors produced by all 11 **c_s_** vectors.

**Conversion of crop data into diversity metrics**

We calculated three metrics of plant commodity diversity: taxonomic species richness (SR), phylogenetic diversity (PSV), and species evenness (E). Each metric was calculated for each country in each year across all produced or consumed plants. SR*_kt_* is the total number of produced or consumed species in country *k* in year *t*. If only looking at food species then SR*_kt_* is the number of produced or consumed species with a positive kilocalorie count in country *k* in year *t*. We use phylogenetic species variability, PSV*_kt_*, as the metric of produced or consumed phylogenetic diversity in country *k* in year *t*,

$PSV_{kt}=\frac{SR_{kt}\times trC_{kt}-\sum C_{kt}}{SR_{kt}\left(1-SR_{kt}\right)}$ (A)

were $\sum C_{kt}$ is the phylogenetic variance-covariance matrix of the evolutionary relatedness of all produced or consumed species in country *k* in year *t* and *tr C_kt_* indicates a sum of the diagonal elements of *C_kt_*. If only looking at food crops then PSV*_kt_* only includes produced or consumed species with a positive kilocalorie count in country *k* in year *t*. The PSV metric is a measure of the average relatedness of the species with a statistical expectation that is independent of species richness. Higher values indicate a set of more distantly related plant species while lower values indicate a set of more closely related species^23^. E*_kt_* is Simpson’s evenness over produced or consumed species,

$E_{kt}=\frac{1}{\sum_{i=1}^{SR_{kt}} p_{skt}^{2}}\times\frac{1}{SR_{kt}}$ (B)

where *p_skt_* = *m_skt_* / *m_kt_*, *m_skt_* is the metric tons of species *s* produced or consumed in country *k* in year *t*, and *m_kt_* that is the metric tons of all species produced or consumed in country *k* in year *t*. Plant phylogenetic relationships are from [51]

**MATLAB Code and Data**

To create a set of **p**, **i**, **e**, and **c** vectors we used the following algorithm.

1. Use importsscript.m to generate importfinal. Save importfinal as importfinal.mat with the following data structure:

[country year cropcode calculatedimports]

1. Use exportsscript.m to generate exportsfinal. Save exportsfinal as exportsfinal.mat with the following data structure:

[country year cropcode calculatedexports]

1. Use correctexports.m to generate concantanateproduction. Save concantanateproduction as productionandexports.mat with the following data structure:

[uniqueID production correctedexports calculatedexports]

1. Use balanceimportsexports.m to generate all final production, imports, exports, and consumption values and save as balanced.mat.

An iteration of steps 1 – 4 will produce a different set of **i**, **e**, and **c** vectors if a different set of **w***_fi_* and **w***_fek_* are used.

S3 File contains the **p** and **c** vectors used in the text. The structure of the dataset in the S3 File is:

Column 1 – “UN FAO country code”

Column 2 – “year”

Column 3 – “crop code”

Column 4 – Concatenated “country code”, “year”, and “crop code”

Column 5 – “metric tons of domestic production”

Column 6 – “metric tons of imports”

Column 7 – “metric tons of exports”

Column 8 – “metric tons of consumption (domestic production less exports plus imports)”

S4 – S13 Files contain the 10 alternative **c** vectors (these PSV, SR, and E data are calculated over all plants). The alternative country-level consumption PSV, SR, and E vectors derived from these 10 alternative **c** vectors are graphed in S3 Fig. The structure of the datasets in the S4 – S13 Files are:

Column 1 – “UN FAO country code”

Column 2 – “year”

Column 3 – “crop code”

Column 4 – Concatenated “country code”, “year”, and “crop code”

Column 5 – “metric tons of domestic production”

Column 6 – “metric tons of imports”

Column 7 – “metric tons of exports”

Column 8 – “metric tons of consumption (domestic production less exports plus imports)”

S14 File contains the country-level PSV, SR, and E data used in the text (these PSV, SR, and E data are calculated over all plants). The structure of the dataset in the S14 File is:

Column 1 – “UN FAO country code”

Column 2 – “year”

Column 3 – “PSVP” (production PSV)

Column 4 – “SRP” (production SR)

Column 5 – “EP” (production E)

Column 6 – “PSVC” (consumption PSV)

Column 7 – “SRC” (consumption SR)

Column 8 – “EC” (consumption E)

Column 9 – “tropical” ( = 1 if the country is in the tropical region and equals 0 otherwise)

S15 File contains the country-level PSV, SR, and E data used in the text for food plants only. The structure of the dataset in the S15 File is:

Column 1 – “UN FAO country code”

Column 2 – “year”

Column 3 – “PSVP” (production PSV)

Column 4 – “SRP” (production SR)

Column 5 – “EP” (weighted by kcals) (production E)

Column 6 – “EP” (weighted by Mg) (production E)

Column 7 – “PSVC” (consumption PSV)

Column 8 – “SRC” (consumption SR)

Column 9 – “EC” (weighted by kcals) (consumption E)

Column 10 – “EC” (weighted by Mg) (consumption E)

S16 – S25 Files contain the alternative country-level consumption PSV, SR, and E vectors generated with the 10 alternative **c** vectors. The structure of the datasets in the S16 – S25 files are:

Column 1 – “UN FAO country code”

Column 2 – “year”

Column 3 – “PSVC” (consumption PSV)

Column 4 – “SRC” (consumption SR)

Column 5 – “EC” (consumption E)

S26 contains country-level arable area (ha) and population data. These data are used to construct Fig 1. The structure of the dataset in the S26 file is:

Column 1 – “UN FAO country code”

Column 2 – “year”

Column 3 – “Arable hectares”

Column 4 – “Population”

S27 File contains the country-level data used to estimate model (2) (all plants). The structure of the dataset in the S27 File is:

Column 1 – “UN FAO country code”

Column 2 – “year”

Column 3 – “gdp per capita”

Column 4 – “population”

Column 5 – “trade openness”

Column 6 – “import value”

Column 7 – “export value”

Column 8 – “SRP” (production SR)

Column 9 – “PSVP” (production PSV)

Column 10 – “EP” (production E)

Column 11 – “SRC” (consumption SR)

Column 12 – “PSVC” (consumption PSV)

Column 13 – “EC” (consumption E)

Column 14 – “longi” (capital longitude)

Column 15 – “lati” (capital latitude)

S28 File contains the country-level data used to estimate model (2) (food plants only). The structure of the dataset in the S28 File is:

Column 1 – “UN FAO country code”

Column 2 – “year”

Column 3 – “gdp per capita”

Column 4 – “population”

Column 5 – “trade openness”

Column 6 – “import value”

Column 7 – “export value”

Column 8 – “SRP” (production SR)

Column 9 – “PSVP” (production PSV)

Column 10 – “EP” (weighted by kcals) (production E)

Column 11 – “SRC” (consumption SR)

Column 12 – “PSVC” (consumption PSV)

Column 13 – “EC” (weighted by kcals) (consumption E)

Column 14 – “longi” (capital longitude)

Column 15 – “lati” (capital latitude)

S29 File contains the country-level data used to estimate model (3) (all plants). The structure of the dataset in the S29 File is:

Column 1 – “UN FAO country code”

Column 2 – “year”

Column 3 – “gdp per capita”

Column 4 – “population”

Column 5 – “trade openness”

Column 6 – “import value”

Column 7 – “export value”

Column 8 – “SRP” (production SR)

Column 9 – “PSVP” (production PSV)

Column 10 – “EP” (production E)

Column 11 – “SRC” (consumption SR)

Column 12 – “PSVC” (consumption PSV)

Column 13 – “EC” (consumption E)

Column 14 – “longi” (capital longitude)

Column 15 – “lati” (capital latitude)

Column 16 – “nra”

Column 17 – “tbi”
