# Supplementary material for: Commercial Plant Production and Consumption Still Follow the Latitudinal Gradient in Species Diversity despite Economic Globalization
Source: PLoS One. 2016 Oct 5;11(10):e0163002. doi: 10.1371/journal.pone.0163002 (PMC5051709; doi:10.1371/journal.pone.0163002)
Supplement: S2 File — (DOCX) [file pone.0163002.s005.docx]

**S2 File. SAR estimates of models (2) and (3)**

**SAR estimates of static model (2) (summarized in Table 2)**

Production PSV, All Crops, 1992 – 2010

SAR with spatial fixed-effects Number of obs = 2538

Group variable: id Number of groups = 141

Time variable: year Panel length = 18

R-sq: within = 0.0001

between = 0.0640

overall = 0.0007

Mean of fixed-effects = 0.0002

Log-likelihood = 10392.5399

---------------------------------------------------------------------------------

ydiffpsvp | Coef. Std. Err. z P>|z| [95% Conf. Interval]

----------------+----------------------------------------------------------------

Main |

gdpcdiff | -.0003579 .0032309 -0.11 0.912 -.0066903 .0059745

gdpcdifflat | .0000308 .0000965 0.32 0.750 -.0001583 .0002198

opennessdiff | -.0004362 .0010353 -0.42 0.674 -.0024654 .001593

opennessdifflat | .0000148 .0000349 0.42 0.673 -.0000537 .0000832

----------------+----------------------------------------------------------------

Spatial |

rho | -.0953269 .0771238 -1.24 0.216 -.2464867 .055833

----------------+----------------------------------------------------------------

Variance |

sigma2_e | .0000162 4.56e-07 35.62 0.000 .0000154 .0000171

----------------+----------------------------------------------------------------

Direct |

gdpcdiff | -.0004089 .0027408 -0.15 0.881 -.0057807 .004963

gdpcdifflat | .0000361 .0000942 0.38 0.701 -.0001485 .0002208

opennessdiff | -.0003787 .0011179 -0.34 0.735 -.0025697 .0018122

opennessdifflat | .0000133 .0000369 0.36 0.719 -.000059 .0000855

----------------+----------------------------------------------------------------

Indirect |

gdpcdiff | .0000267 .0002633 0.10 0.919 -.0004893 .0005427

gdpcdifflat | -2.15e-06 9.23e-06 -0.23 0.816 -.0000202 .0000159

opennessdiff | .000018 .0000966 0.19 0.852 -.0001714 .0002074

opennessdifflat | -7.24e-07 3.58e-06 -0.20 0.840 -7.75e-06 6.30e-06

----------------+----------------------------------------------------------------

Total |

gdpcdiff | -.0003822 .0025366 -0.15 0.880 -.0053538 .0045894

gdpcdifflat | .000034 .0000877 0.39 0.698 -.000138 .000206

opennessdiff | -.0003607 .0010522 -0.34 0.732 -.002423 .0017015

opennessdifflat | .0000125 .0000343 0.37 0.715 -.0000546 .0000797

---------------------------------------------------------------------------------

Production SR, All Crops, 1992 – 2010

SAR with spatial fixed-effects Number of obs = 2538

Group variable: id Number of groups = 141

Time variable: year Panel length = 18

R-sq: within = 0.0007

between = 0.0081

overall = 0.0003

Mean of fixed-effects = 0.0028

Log-likelihood = 6160.5966

---------------------------------------------------------------------------------

ydiffsrp | Coef. Std. Err. z P>|z| [95% Conf. Interval]

----------------+----------------------------------------------------------------

Main |

gdpcdiff | -.0092876 .017119 -0.54 0.587 -.0428402 .024265

gdpcdifflat | .0000251 .0005113 0.05 0.961 -.000977 .0010272

opennessdiff | .003351 .0054795 0.61 0.541 -.0073886 .0140906

opennessdifflat | -7.78e-06 .0001849 -0.04 0.966 -.0003701 .0003545

----------------+----------------------------------------------------------------

Spatial |

rho | .207606 .0637945 3.25 0.001 .0825711 .3326409

----------------+----------------------------------------------------------------

Variance |

sigma2_e | .0004555 .0000128 35.60 0.000 .0004304 .0004806

----------------+----------------------------------------------------------------

Direct |

gdpcdiff | -.0095754 .0145429 -0.66 0.510 -.038079 .0189282

gdpcdifflat | .0000535 .0005002 0.11 0.915 -.0009269 .0010339

opennessdiff | .0036621 .0059264 0.62 0.537 -.0079533 .0152776

opennessdifflat | -.0000158 .0001954 -0.08 0.936 -.0003988 .0003673

----------------+----------------------------------------------------------------

Indirect |

gdpcdiff | -.0027452 .004354 -0.63 0.528 -.011279 .0057886

gdpcdifflat | .0000169 .0001488 0.11 0.910 -.0002748 .0003085

opennessdiff | .0009561 .0018167 0.53 0.599 -.0026046 .0045168

opennessdifflat | -2.27e-06 .0000578 -0.04 0.969 -.0001156 .0001111

----------------+----------------------------------------------------------------

Total |

gdpcdiff | -.0123206 .0186365 -0.66 0.509 -.0488474 .0242062

gdpcdifflat | .0000703 .0006429 0.11 0.913 -.0011897 .0013304

opennessdiff | .0046182 .0076612 0.60 0.547 -.0103975 .0196339

opennessdifflat | -.000018 .0002511 -0.07 0.943 -.0005102 .0004741

---------------------------------------------------------------------------------

Production E, All Crops, 1992 – 2010

SAR with spatial fixed-effects Number of obs = 2538

Group variable: id Number of groups = 141

Time variable: year Panel length = 18

R-sq: within = 0.0038

between = 0.0309

overall = 0.0017

Mean of fixed-effects = 0.0006

Log-likelihood = 6510.2045

---------------------------------------------------------------------------------

ydiffshp | Coef. Std. Err. z P>|z| [95% Conf. Interval]

----------------+----------------------------------------------------------------

Main |

gdpcdiff | -.0102697 .0149134 -0.69 0.491 -.0394994 .0189601

gdpcdifflat | .0007824 .0004455 1.76 0.079 -.0000907 .0016556

opennessdiff | -.0090831 .004777 -1.90 0.057 -.0184457 .0002796

opennessdifflat | .0001869 .0001612 1.16 0.246 -.0001291 .0005028

----------------+----------------------------------------------------------------

Spatial |

rho | .1353763 .0679455 1.99 0.046 .0022057 .268547

----------------+----------------------------------------------------------------

Variance |

sigma2_e | .0003461 9.72e-06 35.61 0.000 .0003271 .0003652

----------------+----------------------------------------------------------------

Direct |

gdpcdiff | -.0105145 .0126573 -0.83 0.406 -.0353223 .0142934

gdpcdifflat | .0008079 .0004355 1.85 0.064 -.0000457 .0016615

opennessdiff | -.0088265 .0051623 -1.71 0.087 -.0189445 .0012915

opennessdifflat | .0001801 .0001702 1.06 0.290 -.0001536 .0005138

----------------+----------------------------------------------------------------

Indirect |

gdpcdiff | -.0019105 .0025998 -0.73 0.462 -.0070059 .0031849

gdpcdifflat | .0001474 .0001145 1.29 0.198 -.000077 .0003718

opennessdiff | -.0016606 .0014172 -1.17 0.241 -.0044383 .001117

opennessdifflat | .0000339 .0000384 0.88 0.377 -.0000414 .0001092

----------------+----------------------------------------------------------------

Total |

gdpcdiff | -.012425 .0148948 -0.83 0.404 -.0416183 .0167684

gdpcdifflat | .0009553 .0005208 1.83 0.067 -.0000655 .001976

opennessdiff | -.0104871 .0063182 -1.66 0.097 -.0228706 .0018964

opennessdifflat | .000214 .0002026 1.06 0.291 -.0001832 .0006112

---------------------------------------------------------------------------------

Consumption PSV, All Crops, 1992 – 2010

SAR with spatial fixed-effects Number of obs = 2538

Group variable: id Number of groups = 141

Time variable: year Panel length = 18

R-sq: within = 0.0074

between = 0.2055

overall = 0.0031

Mean of fixed-effects = 0.0008

Log-likelihood = 7777.3927

---------------------------------------------------------------------------------

ydiffpsvc | Coef. Std. Err. z P>|z| [95% Conf. Interval]

----------------+----------------------------------------------------------------

Main |

gdpcdiff | .0143088 .0090534 1.58 0.114 -.0034356 .0320531

gdpcdifflat | -.0008927 .0002708 -3.30 0.001 -.0014234 -.000362

opennessdiff | .0000442 .0029001 0.02 0.988 -.0056399 .0057283

opennessdifflat | .0000892 .0000978 0.91 0.362 -.0001025 .000281

----------------+----------------------------------------------------------------

Spatial |

rho | .0822907 .0718925 1.14 0.252 -.058616 .2231973

----------------+----------------------------------------------------------------

Variance |

sigma2_e | .0001276 3.58e-06 35.62 0.000 .0001205 .0001346

----------------+----------------------------------------------------------------

Direct |

gdpcdiff | .0141733 .0076799 1.85 0.065 -.0008791 .0292257

gdpcdifflat | -.0008781 .0002646 -3.32 0.001 -.0013968 -.0003595

opennessdiff | .0002059 .0031324 0.07 0.948 -.0059336 .0063454

opennessdifflat | .000085 .0001033 0.82 0.410 -.0001174 .0002875

----------------+----------------------------------------------------------------

Indirect |

gdpcdiff | .0015882 .0014881 1.07 0.286 -.0013284 .0045048

gdpcdifflat | -.0000965 .000076 -1.27 0.204 -.0002454 .0000524

opennessdiff | -.0000106 .0004519 -0.02 0.981 -.0008963 .0008751

opennessdifflat | .0000107 .0000167 0.64 0.521 -.000022 .0000435

----------------+----------------------------------------------------------------

Total |

gdpcdiff | .0157614 .0085307 1.85 0.065 -.0009584 .0324812

gdpcdifflat | -.0009746 .0002912 -3.35 0.001 -.0015454 -.0004038

opennessdiff | .0001953 .0035234 0.06 0.956 -.0067105 .0071011

opennessdifflat | .0000958 .0001159 0.83 0.409 -.0001314 .0003229

---------------------------------------------------------------------------------

Consumption SR, All Crops, 1992 – 2010

SAR with spatial fixed-effects Number of obs = 2538

Group variable: id Number of groups = 141

Time variable: year Panel length = 18

R-sq: within = 0.0565

between = 0.0042

overall = 0.0058

Mean of fixed-effects = 0.2184

Log-likelihood = 2454.2297

---------------------------------------------------------------------------------

ydiffsrc | Coef. Std. Err. z P>|z| [95% Conf. Interval]

----------------+----------------------------------------------------------------

Main |

gdpcdiff | .1538721 .0733 2.10 0.036 .0102068 .2975375

gdpcdifflat | .0045519 .0021852 2.08 0.037 .000269 .0088348

opennessdiff | .049035 .0234283 2.09 0.036 .0031163 .0949536

opennessdifflat | -.0016223 .0007905 -2.05 0.040 -.0031715 -.000073

----------------+----------------------------------------------------------------

Spatial |

rho | .6198054 .0381837 16.23 0.000 .5449667 .694644

----------------+----------------------------------------------------------------

Variance |

sigma2_e | .008328 .0002347 35.49 0.000 .0078681 .0087879

----------------+----------------------------------------------------------------

Direct |

gdpcdiff | .1558522 .0633782 2.46 0.014 .0316332 .2800711

gdpcdifflat | .0047702 .0021829 2.19 0.029 .0004918 .0090486

opennessdiff | .0513608 .0257918 1.99 0.046 .0008098 .1019118

opennessdifflat | -.0016902 .0008504 -1.99 0.047 -.003357 -.0000234

----------------+----------------------------------------------------------------

Indirect |

gdpcdiff | .2564228 .1079325 2.38 0.018 .044879 .4679666

gdpcdifflat | .0078762 .0037966 2.07 0.038 .000435 .0153173

opennessdiff | .0837954 .0434321 1.93 0.054 -.0013299 .1689207

opennessdifflat | -.002767 .0014143 -1.96 0.050 -.005539 4.87e-06

----------------+----------------------------------------------------------------

Total |

gdpcdiff | .4122749 .1684629 2.45 0.014 .0820937 .7424562

gdpcdifflat | .0126464 .0058914 2.15 0.032 .0010994 .0241933

opennessdiff | .1351562 .0684097 1.98 0.048 .0010756 .2692367

opennessdifflat | -.0044573 .0022377 -1.99 0.046 -.0088431 -.0000714

---------------------------------------------------------------------------------

Consumption E, All Crops, 1992 – 2010

SAR with spatial fixed-effects Number of obs = 2538

Group variable: id Number of groups = 141

Time variable: year Panel length = 18

R-sq: within = 0.0011

between = 0.0158

overall = 0.0008

Mean of fixed-effects = -0.0002

Log-likelihood = 4337.0594

---------------------------------------------------------------------------------

ydiffshc | Coef. Std. Err. z P>|z| [95% Conf. Interval]

----------------+----------------------------------------------------------------

Main |

gdpcdiff | -.0300287 .0351127 -0.86 0.392 -.0988483 .0387909

gdpcdifflat | .0008005 .0010484 0.76 0.445 -.0012542 .0028552

opennessdiff | .0046339 .0112695 0.41 0.681 -.0174539 .0267218

opennessdifflat | -.0004323 .0003799 -1.14 0.255 -.0011769 .0003124

----------------+----------------------------------------------------------------

Spatial |

rho | .0982626 .0695866 1.41 0.158 -.0381247 .2346498

----------------+----------------------------------------------------------------

Variance |

sigma2_e | .0019191 .0000539 35.62 0.000 .0018135 .0020247

----------------+----------------------------------------------------------------

Direct |

gdpcdiff | -.0306002 .0297914 -1.03 0.304 -.0889903 .02779

gdpcdifflat | .0008591 .0010245 0.84 0.402 -.0011489 .0028671

opennessdiff | .0052618 .012171 0.43 0.666 -.018593 .0291166

opennessdifflat | -.0004489 .0004011 -1.12 0.263 -.0012351 .0003373

----------------+----------------------------------------------------------------

Indirect |

gdpcdiff | -.0040958 .005136 -0.80 0.425 -.0141621 .0059705

gdpcdifflat | .0001186 .0001766 0.67 0.502 -.0002275 .0004646

opennessdiff | .0006111 .0019315 0.32 0.752 -.0031746 .0043968

opennessdifflat | -.0000577 .0000659 -0.88 0.381 -.0001868 .0000715

----------------+----------------------------------------------------------------

Total |

gdpcdiff | -.0346959 .0337044 -1.03 0.303 -.1007554 .0313636

gdpcdifflat | .0009777 .0011657 0.84 0.402 -.0013071 .0032624

opennessdiff | .0058729 .0138702 0.42 0.672 -.0213122 .033058

opennessdifflat | -.0005066 .0004515 -1.12 0.262 -.0013916 .0003784

---------------------------------------------------------------------------------

Production PSV, Food Crops, 1992 – 2010

SAR with spatial fixed-effects Number of obs = 2538

Group variable: id Number of groups = 141

Time variable: year Panel length = 18

R-sq: within = 0.0002

between = 0.0440

overall = 0.0007

Mean of fixed-effects = 0.0002

Log-likelihood = 10289.6334

---------------------------------------------------------------------------------

ydiffpsvp | Coef. Std. Err. z P>|z| [95% Conf. Interval]

----------------+----------------------------------------------------------------

Main |

gdpcdiff | -.0011692 .0033649 -0.35 0.728 -.0077643 .0054259

gdpcdifflat | .0000546 .0001005 0.54 0.587 -.0001423 .0002515

opennessdiff | -.0005751 .0010778 -0.53 0.594 -.0026876 .0015374

opennessdifflat | .0000151 .0000364 0.42 0.677 -.0000561 .0000864

----------------+----------------------------------------------------------------

Spatial |

rho | -.1031423 .0777479 -1.33 0.185 -.2555254 .0492407

----------------+----------------------------------------------------------------

Variance |

sigma2_e | .0000176 4.95e-07 35.62 0.000 .0000166 .0000186

----------------+----------------------------------------------------------------

Direct |

gdpcdiff | -.0012227 .0028547 -0.43 0.668 -.0068177 .0043723

gdpcdifflat | .0000602 .0000981 0.61 0.540 -.0001322 .0002526

opennessdiff | -.0005153 .0011638 -0.44 0.658 -.0027963 .0017657

opennessdifflat | .0000136 .0000384 0.35 0.723 -.0000616 .0000888

----------------+----------------------------------------------------------------

Indirect |

gdpcdiff | .0000925 .0003011 0.31 0.759 -.0004976 .0006826

gdpcdifflat | -4.25e-06 .0000106 -0.40 0.687 -.0000249 .0000164

opennessdiff | .0000298 .0001073 0.28 0.781 -.0001804 .00024

opennessdifflat | -8.05e-07 3.90e-06 -0.21 0.837 -8.45e-06 6.85e-06

----------------+----------------------------------------------------------------

Total |

gdpcdiff | -.0011302 .0026243 -0.43 0.667 -.0062738 .0040134

gdpcdifflat | .0000559 .0000908 0.62 0.538 -.0001221 .000234

opennessdiff | -.0004855 .0010894 -0.45 0.656 -.0026207 .0016496

opennessdifflat | .0000128 .0000355 0.36 0.719 -.0000567 .0000823

---------------------------------------------------------------------------------

Production SR, Food Crops, 1992 – 2010

SAR with spatial fixed-effects Number of obs = 2538

Group variable: id Number of groups = 141

Time variable: year Panel length = 18

R-sq: within = 0.0017

between = 0.1291

overall = 0.0054

Mean of fixed-effects = 0.0027

Log-likelihood = 6155.1601

---------------------------------------------------------------------------------

ydiffsrp | Coef. Std. Err. z P>|z| [95% Conf. Interval]

----------------+----------------------------------------------------------------

Main |

gdpcdiff | -.0191633 .0171534 -1.12 0.264 -.0527832 .0144567

gdpcdifflat | .0006258 .0005131 1.22 0.223 -.0003798 .0016314

opennessdiff | -.0001501 .0054905 -0.03 0.978 -.0109112 .0106111

opennessdifflat | .0001058 .0001853 0.57 0.568 -.0002575 .000469

----------------+----------------------------------------------------------------

Spatial |

rho | .2341441 .0628751 3.72 0.000 .1109112 .357377

----------------+----------------------------------------------------------------

Variance |

sigma2_e | .0004573 .0000128 35.60 0.000 .0004321 .0004825

----------------+----------------------------------------------------------------

Direct |

gdpcdiff | -.01948 .0145787 -1.34 0.181 -.0480536 .0090937

gdpcdifflat | .0006558 .0005022 1.31 0.192 -.0003286 .0016401

opennessdiff | .0001544 .0059413 0.03 0.979 -.0114903 .011799

opennessdifflat | .0000981 .000196 0.50 0.617 -.0002861 .0004822

----------------+----------------------------------------------------------------

Indirect |

gdpcdiff | -.0064507 .0052876 -1.22 0.222 -.0168143 .0039129

gdpcdifflat | .0002177 .0001845 1.18 0.238 -.000144 .0005794

opennessdiff | -.0000367 .0021062 -0.02 0.986 -.0041648 .0040913

opennessdifflat | .0000342 .0000683 0.50 0.616 -.0000997 .0001681

----------------+----------------------------------------------------------------

Total |

gdpcdiff | -.0259307 .0193849 -1.34 0.181 -.0639243 .012063

gdpcdifflat | .0008734 .0006706 1.30 0.193 -.0004409 .0021878

opennessdiff | .0001176 .0079938 0.01 0.988 -.0155499 .0157851

opennessdifflat | .0001323 .0002615 0.51 0.613 -.0003803 .0006448

---------------------------------------------------------------------------------

Production E, Food Crops, 1992 – 2010

SAR with spatial fixed-effects Number of obs = 2538

Group variable: id Number of groups = 141

Time variable: year Panel length = 18

R-sq: within = 0.0057

between = 0.0047

overall = 0.0055

Mean of fixed-effects = -0.0001

Log-likelihood = 9013.9638

---------------------------------------------------------------------------------

ydiffshp | Coef. Std. Err. z P>|z| [95% Conf. Interval]

----------------+----------------------------------------------------------------

Main |

gdpcdiff | -.0026903 .0055607 -0.48 0.629 -.013589 .0082085

gdpcdifflat | -.000268 .000166 -1.61 0.106 -.0005933 .0000574

opennessdiff | -.0004797 .0017811 -0.27 0.788 -.0039706 .0030113

opennessdifflat | .00005 .0000601 0.83 0.405 -.0000678 .0001678

----------------+----------------------------------------------------------------

Spatial |

rho | .1226127 .0701692 1.75 0.081 -.0149165 .2601418

----------------+----------------------------------------------------------------

Variance |

sigma2_e | .0000481 1.35e-06 35.61 0.000 .0000455 .0000508

----------------+----------------------------------------------------------------

Direct |

gdpcdiff | -.0027802 .004719 -0.59 0.556 -.0120292 .0064688

gdpcdifflat | -.000259 .0001622 -1.60 0.110 -.000577 .000059

opennessdiff | -.0003811 .0019244 -0.20 0.843 -.0041528 .0033906

opennessdifflat | .0000475 .0000635 0.75 0.455 -.0000769 .0001718

----------------+----------------------------------------------------------------

Indirect |

gdpcdiff | -.0004727 .0008795 -0.54 0.591 -.0021965 .0012511

gdpcdifflat | -.0000413 .000035 -1.18 0.237 -.0001098 .0000272

opennessdiff | -.0000881 .0003771 -0.23 0.815 -.0008272 .0006511

opennessdifflat | 8.45e-06 .0000128 0.66 0.509 -.0000166 .0000335

----------------+----------------------------------------------------------------

Total |

gdpcdiff | -.0032529 .0054794 -0.59 0.553 -.0139923 .0074864

gdpcdifflat | -.0003003 .0001878 -1.60 0.110 -.0006683 .0000677

opennessdiff | -.0004691 .0022707 -0.21 0.836 -.0049196 .0039814

opennessdifflat | .0000559 .0000744 0.75 0.452 -.0000899 .0002017

---------------------------------------------------------------------------------

Consumption PSV, Food Crops, 1992 – 2010

SAR with spatial fixed-effects Number of obs = 2538

Group variable: id Number of groups = 141

Time variable: year Panel length = 18

R-sq: within = 0.0110

between = 0.2124

overall = 0.0050

Mean of fixed-effects = 0.0009

Log-likelihood = 7812.3368

---------------------------------------------------------------------------------

ydiffpsvc | Coef. Std. Err. z P>|z| [95% Conf. Interval]

----------------+----------------------------------------------------------------

Main |

gdpcdiff | .0172026 .00893 1.93 0.054 -.0002998 .034705

gdpcdifflat | -.0011191 .0002673 -4.19 0.000 -.001643 -.0005952

opennessdiff | -.0000198 .0028603 -0.01 0.994 -.0056259 .0055862

opennessdifflat | .0000766 .0000965 0.79 0.427 -.0001125 .0002658

----------------+----------------------------------------------------------------

Spatial |

rho | .0269814 .0725078 0.37 0.710 -.1151313 .1690941

----------------+----------------------------------------------------------------

Variance |

sigma2_e | .0001241 3.48e-06 35.62 0.000 .0001173 .000131

----------------+----------------------------------------------------------------

Direct |

gdpcdiff | .0170659 .0075734 2.25 0.024 .0022222 .0319096

gdpcdifflat | -.0011045 .0002613 -4.23 0.000 -.0016167 -.0005923

opennessdiff | .0001394 .0030883 0.05 0.964 -.0059136 .0061924

opennessdifflat | .0000725 .0001019 0.71 0.477 -.0001271 .0002722

----------------+----------------------------------------------------------------

Indirect |

gdpcdiff | .0008254 .0013862 0.60 0.552 -.0018915 .0035424

gdpcdifflat | -.0000513 .0000832 -0.62 0.538 -.0002144 .0001119

opennessdiff | -.0000262 .0002778 -0.09 0.925 -.0005707 .0005182

opennessdifflat | 4.62e-06 .0000111 0.42 0.677 -.0000171 .0000263

----------------+----------------------------------------------------------------

Total |

gdpcdiff | .0178913 .0079528 2.25 0.024 .002304 .0334786

gdpcdifflat | -.0011558 .000271 -4.27 0.000 -.0016869 -.0006247

opennessdiff | .0001132 .0032729 0.03 0.972 -.0063017 .006528

opennessdifflat | .0000771 .0001076 0.72 0.474 -.0001339 .0002881

--------------------------------------------------------------------------------

Consumption SR, Food Crops, 1992 – 2010

SAR with spatial fixed-effects Number of obs = 2538

Group variable: id Number of groups = 141

Time variable: year Panel length = 18

R-sq: within = 0.0547

between = 0.0046

overall = 0.0057

Mean of fixed-effects = 0.1971

Log-likelihood = 2695.3823

---------------------------------------------------------------------------------

ydiffsrc | Coef. Std. Err. z P>|z| [95% Conf. Interval]

----------------+----------------------------------------------------------------

Main |

gdpcdiff | .1090763 .0666541 1.64 0.102 -.0215633 .2397159

gdpcdifflat | .0052622 .0019894 2.65 0.008 .0013631 .0091613

opennessdiff | .0537001 .0213197 2.52 0.012 .0119143 .095486

opennessdifflat | -.0017176 .0007193 -2.39 0.017 -.0031273 -.0003078

----------------+----------------------------------------------------------------

Spatial |

rho | .5983781 .0387437 15.44 0.000 .5224419 .6743142

----------------+----------------------------------------------------------------

Variance |

sigma2_e | .0068958 .0001942 35.50 0.000 .0065151 .0072765

----------------+----------------------------------------------------------------

Direct |

gdpcdiff | .110036 .057537 1.91 0.056 -.0027345 .2228064

gdpcdifflat | .0054736 .0019834 2.76 0.006 .0015862 .0093609

opennessdiff | .055897 .0234231 2.39 0.017 .0099886 .1018055

opennessdifflat | -.001781 .0007724 -2.31 0.021 -.0032949 -.0002671

----------------+----------------------------------------------------------------

Indirect |

gdpcdiff | .1655939 .0880459 1.88 0.060 -.0069729 .3381608

gdpcdifflat | .0082653 .0032342 2.56 0.011 .0019264 .0146042

opennessdiff | .0836899 .0365483 2.29 0.022 .0120567 .1553232

opennessdifflat | -.0026735 .0011902 -2.25 0.025 -.0050062 -.0003408

----------------+----------------------------------------------------------------

Total |

gdpcdiff | .2756299 .1439835 1.91 0.056 -.0065725 .5578323

gdpcdifflat | .0137389 .0051071 2.69 0.007 .003729 .0237487

opennessdiff | .139587 .0590146 2.37 0.018 .0239205 .2552534

opennessdifflat | -.0044545 .0019327 -2.30 0.021 -.0082425 -.0006665

---------------------------------------------------------------------------------

Consumption E, Food Crops, 1992 – 2010

SAR with spatial fixed-effects Number of obs = 2538

Group variable: id Number of groups = 141

Time variable: year Panel length = 18

R-sq: within = 0.0044

between = 0.0160

overall = 0.0044

Mean of fixed-effects = -0.0009

Log-likelihood = 6101.7626

---------------------------------------------------------------------------------

ydiffshc | Coef. Std. Err. z P>|z| [95% Conf. Interval]

----------------+----------------------------------------------------------------

Main |

gdpcdiff | -.0591913 .0175182 -3.38 0.001 -.0935263 -.0248563

gdpcdifflat | .0013661 .000523 2.61 0.009 .000341 .0023912

opennessdiff | -.0004741 .0056141 -0.08 0.933 -.0114775 .0105293

opennessdifflat | .0000332 .0001893 0.18 0.861 -.0003379 .0004043

----------------+----------------------------------------------------------------

Spatial |

rho | .0981803 .0718638 1.37 0.172 -.04267 .2390307

----------------+----------------------------------------------------------------

Variance |

sigma2_e | .0004777 .0000134 35.62 0.000 .0004514 .000504

----------------+----------------------------------------------------------------

Direct |

gdpcdiff | -.059504 .0148649 -4.00 0.000 -.0886386 -.0303693

gdpcdifflat | .0013959 .0005112 2.73 0.006 .000394 .0023979

opennessdiff | -.0001628 .0060637 -0.03 0.979 -.0120474 .0117219

opennessdifflat | .000025 .0001999 0.13 0.900 -.0003667 .0004168

----------------+----------------------------------------------------------------

Indirect |

gdpcdiff | -.0079575 .0057396 -1.39 0.166 -.0192068 .0032918

gdpcdifflat | .0001885 .0001505 1.25 0.210 -.0001064 .0004834

opennessdiff | -.000085 .0009833 -0.09 0.931 -.0020123 .0018423

opennessdifflat | 4.94e-06 .0000303 0.16 0.870 -.0000544 .0000643

----------------+----------------------------------------------------------------

Total |

gdpcdiff | -.0674615 .017676 -3.82 0.000 -.1021059 -.0328171

gdpcdifflat | .0015845 .0005988 2.65 0.008 .0004109 .002758

opennessdiff | -.0002478 .0069424 -0.04 0.972 -.0138545 .013359

opennessdifflat | .00003 .000226 0.13 0.894 -.000413 .000473

---------------------------------------------------------------------------------

**SAR estimates of model (2) with contemporaneous and once lagged changes in independent variables (summarized in S1 Table)**

Production PSV, Total Crops, 1992 – 2010

SAR with spatial fixed-effects Number of obs = 2397

Group variable: id Number of groups = 141

Time variable: year Panel length = 17

R-sq: within = 0.0026

between = 0.0243

overall = 0.0031

Mean of fixed-effects = 0.0003

Log-likelihood = 9847.9287

---------------------------------------------------------------------------------------

ydiffpsvp | Coef. Std. Err. z P>|z| [95% Conf. Interval]

----------------------+----------------------------------------------------------------

Main |

gdpcfirstdiff | -.0011849 .0033688 -0.35 0.725 -.0077875 .0054178

gdpcfirstdifflat | .0000769 .00011 0.70 0.485 -.0001387 .0002925

gdpcseconddiff | -.0005385 .0032673 -0.16 0.869 -.0069422 .0058653

gdpcseconddifflat | -.0000952 .0001033 -0.92 0.356 -.0002977 .0001072

opennessfirstdiff | -.0008999 .0011346 -0.79 0.428 -.0031237 .0013238

opennessfirstdifflat | .000057 .0000416 1.37 0.170 -.0000244 .0001385

opennessseconddiff | -.000345 .001096 -0.31 0.753 -.0024931 .0018032

opennessseconddifflat | .0000145 .0000358 0.41 0.685 -.0000557 .0000847

----------------------+----------------------------------------------------------------

Spatial |

rho | -.113952 .0787936 -1.45 0.148 -.2683848 .0404807

----------------------+----------------------------------------------------------------

Variance |

sigma2_e | .0000158 4.57e-07 34.61 0.000 .0000149 .0000167

----------------------+----------------------------------------------------------------

Direct |

gdpcfirstdiff | -.0012387 .0028584 -0.43 0.665 -.0068411 .0043637

gdpcfirstdifflat | .0000835 .0001096 0.76 0.447 -.0001314 .0002983

gdpcseconddiff | -.0003305 .0035391 -0.09 0.926 -.007267 .006606

gdpcseconddifflat | -.0001026 .0001105 -0.93 0.353 -.0003191 .000114

opennessfirstdiff | -.0006687 .0011024 -0.61 0.544 -.0028294 .001492

opennessfirstdifflat | .0000547 .0000422 1.30 0.194 -.0000279 .0001373

opennessseconddiff | -.0002589 .0011383 -0.23 0.820 -.0024898 .0019721

opennessseconddifflat | .0000118 .0000362 0.33 0.745 -.0000592 .0000827

----------------------+----------------------------------------------------------------

Indirect |

gdpcfirstdiff | .0001163 .0003559 0.33 0.744 -.0005812 .0008139

gdpcfirstdifflat | -8.91e-06 .0000151 -0.59 0.556 -.0000386 .0000208

gdpcseconddiff | .000066 .0004132 0.16 0.873 -.0007438 .0008758

gdpcseconddifflat | .0000101 .0000143 0.70 0.482 -.000018 .0000382

opennessfirstdiff | .0000694 .0001333 0.52 0.603 -.0001919 .0003306

opennessfirstdifflat | -5.75e-06 5.94e-06 -0.97 0.334 -.0000174 5.90e-06

opennessseconddiff | .0000369 .0001466 0.25 0.801 -.0002503 .0003242

opennessseconddifflat | -1.30e-06 4.33e-06 -0.30 0.764 -9.80e-06 7.20e-06

----------------------+----------------------------------------------------------------

Total |

gdpcfirstdiff | -.0011224 .0026088 -0.43 0.667 -.0062355 .0039907

gdpcfirstdifflat | .0000745 .0000978 0.76 0.446 -.0001171 .0002662

gdpcseconddiff | -.0002645 .0031806 -0.08 0.934 -.0064983 .0059693

gdpcseconddifflat | -.0000925 .0000992 -0.93 0.351 -.0002869 .0001019

opennessfirstdiff | -.0005993 .0009952 -0.60 0.547 -.0025498 .0013512

opennessfirstdifflat | .000049 .0000381 1.29 0.198 -.0000256 .0001236

opennessseconddiff | -.0002219 .0010082 -0.22 0.826 -.0021981 .0017542

opennessseconddifflat | .0000105 .0000325 0.32 0.747 -.0000532 .0000741

---------------------------------------------------------------------------------------

Production SR, Total Crops, 1992 – 2010

SAR with spatial fixed-effects Number of obs = 2397

Group variable: id Number of groups = 141

Time variable: year Panel length = 17

R-sq: within = 0.0034

between = 0.0514

overall = 0.0011

Mean of fixed-effects = 0.0030

Log-likelihood = 5831.0946

---------------------------------------------------------------------------------------

ydiffsrp | Coef. Std. Err. z P>|z| [95% Conf. Interval]

----------------------+----------------------------------------------------------------

Main |

gdpcfirstdiff | -.0054054 .017996 -0.30 0.764 -.0406768 .0298661

gdpcfirstdifflat | -.0004816 .0005877 -0.82 0.413 -.0016335 .0006703

gdpcseconddiff | -.013956 .0174413 -0.80 0.424 -.0481403 .0202283

gdpcseconddifflat | .0004752 .0005515 0.86 0.389 -.0006057 .001556

opennessfirstdiff | -.0008087 .0060586 -0.13 0.894 -.0126834 .0110659

opennessfirstdifflat | .0002687 .0002219 1.21 0.226 -.0001663 .0007036

opennessseconddiff | -.007464 .0058557 -1.27 0.202 -.0189409 .0040129

opennessseconddifflat | .000299 .0001913 1.56 0.118 -.000076 .000674

----------------------+----------------------------------------------------------------

Spatial |

rho | .2227846 .0649619 3.43 0.001 .0954616 .3501076

----------------------+----------------------------------------------------------------

Variance |

sigma2_e | .0004506 .000013 34.60 0.000 .0004251 .0004761

----------------------+----------------------------------------------------------------

Direct |

gdpcfirstdiff | -.0057002 .0152931 -0.37 0.709 -.0356741 .0242737

gdpcfirstdifflat | -.0004477 .0005864 -0.76 0.445 -.001597 .0007015

gdpcseconddiff | -.0128642 .0189145 -0.68 0.496 -.0499359 .0242074

gdpcseconddifflat | .000437 .0005905 0.74 0.459 -.0007203 .0015944

opennessfirstdiff | .0004288 .005897 0.07 0.942 -.011129 .0119867

opennessfirstdifflat | .0002568 .0002254 1.14 0.255 -.000185 .0006986

opennessseconddiff | -.007014 .0060887 -1.15 0.249 -.0189476 .0049197

opennessseconddifflat | .0002848 .0001937 1.47 0.142 -.0000949 .0006645

----------------------+----------------------------------------------------------------

Indirect |

gdpcfirstdiff | -.0016712 .0051099 -0.33 0.744 -.0116864 .008344

gdpcfirstdifflat | -.0001304 .0001754 -0.74 0.457 -.0004742 .0002134

gdpcseconddiff | -.0033277 .0056088 -0.59 0.553 -.0143208 .0076654

gdpcseconddifflat | .0001177 .0001702 0.69 0.489 -.000216 .0004513

opennessfirstdiff | .0000821 .0017835 0.05 0.963 -.0034135 .0035777

opennessfirstdifflat | .0000726 .0000734 0.99 0.323 -.0000713 .0002166

opennessseconddiff | -.0019231 .0017622 -1.09 0.275 -.0053768 .0015307

opennessseconddifflat | .0000809 .0000643 1.26 0.208 -.0000451 .0002069

----------------------+----------------------------------------------------------------

Total |

gdpcfirstdiff | -.0073714 .0200567 -0.37 0.713 -.0466817 .0319389

gdpcfirstdifflat | -.0005782 .0007525 -0.77 0.442 -.0020531 .0008968

gdpcseconddiff | -.0161919 .0241704 -0.67 0.503 -.063565 .0311812

gdpcseconddifflat | .0005547 .0007498 0.74 0.459 -.000915 .0020244

opennessfirstdiff | .0005109 .00762 0.07 0.947 -.0144239 .0154458

opennessfirstdifflat | .0003294 .0002927 1.13 0.260 -.0002442 .000903

opennessseconddiff | -.008937 .0076934 -1.16 0.245 -.0240158 .0061417

opennessseconddifflat | .0003656 .0002516 1.45 0.146 -.0001276 .0008588

---------------------------------------------------------------------------------------

Production E, Total Crops, 1992 – 2010

SAR with spatial fixed-effects Number of obs = 2397

Group variable: id Number of groups = 141

Time variable: year Panel length = 17

R-sq: within = 0.0083

between = 0.0270

overall = 0.0049

Mean of fixed-effects = 0.0007

Log-likelihood = 6179.4502

---------------------------------------------------------------------------------------

ydiffshp | Coef. Std. Err. z P>|z| [95% Conf. Interval]

----------------------+----------------------------------------------------------------

Main |

gdpcfirstdiff | -.0181521 .0155678 -1.17 0.244 -.0486644 .0123601

gdpcfirstdifflat | .0013612 .0005087 2.68 0.007 .0003641 .0023582

gdpcseconddiff | -.0101274 .0150914 -0.67 0.502 -.039706 .0194512

gdpcseconddifflat | -.0001217 .0004772 -0.25 0.799 -.0010571 .0008137

opennessfirstdiff | -.0055463 .0052416 -1.06 0.290 -.0158197 .004727

opennessfirstdifflat | -9.64e-07 .0001921 -0.01 0.996 -.0003774 .0003755

opennessseconddiff | .0076101 .0050628 1.50 0.133 -.0023129 .017533

opennessseconddifflat | -.0003588 .0001654 -2.17 0.030 -.0006829 -.0000346

----------------------+----------------------------------------------------------------

Spatial |

rho | .1116482 .0703094 1.59 0.112 -.0261557 .2494522

----------------------+----------------------------------------------------------------

Variance |

sigma2_e | .0003374 9.75e-06 34.61 0.000 .0003183 .0003565

----------------------+----------------------------------------------------------------

Direct |

gdpcfirstdiff | -.018408 .0132116 -1.39 0.164 -.0443023 .0074862

gdpcfirstdifflat | .001392 .0005069 2.75 0.006 .0003985 .0023855

gdpcseconddiff | -.009168 .0163459 -0.56 0.575 -.0412054 .0228694

gdpcseconddifflat | -.0001554 .0005104 -0.30 0.761 -.0011558 .0008449

opennessfirstdiff | -.0044791 .0050933 -0.88 0.379 -.0144619 .0055036

opennessfirstdifflat | -.0000117 .0001948 -0.06 0.952 -.0003934 .0003701

opennessseconddiff | .0080131 .0052581 1.52 0.128 -.0022925 .0183187

opennessseconddifflat | -.0003716 .0001672 -2.22 0.026 -.0006993 -.000044

----------------------+----------------------------------------------------------------

Indirect |

gdpcfirstdiff | -.0023295 .0030343 -0.77 0.443 -.0082766 .0036176

gdpcfirstdifflat | .000168 .0001401 1.20 0.230 -.0001066 .0004426

gdpcseconddiff | -.000984 .002487 -0.40 0.692 -.0058585 .0038905

gdpcseconddifflat | -.0000214 .0000705 -0.30 0.762 -.0001595 .0001168

opennessfirstdiff | -.0005545 .0008701 -0.64 0.524 -.0022599 .001151

opennessfirstdifflat | -1.98e-06 .0000286 -0.07 0.945 -.000058 .000054

opennessseconddiff | .0010495 .0010922 0.96 0.337 -.0010913 .0031903

opennessseconddifflat | -.0000464 .0000429 -1.08 0.279 -.0001304 .0000376

----------------------+----------------------------------------------------------------

Total |

gdpcfirstdiff | -.0207375 .0153083 -1.35 0.176 -.0507413 .0092663

gdpcfirstdifflat | .00156 .0005698 2.74 0.006 .0004432 .0026769

gdpcseconddiff | -.010152 .0183765 -0.55 0.581 -.0461693 .0258653

gdpcseconddifflat | -.0001768 .0005707 -0.31 0.757 -.0012953 .0009417

opennessfirstdiff | -.0050336 .0057797 -0.87 0.384 -.0163616 .0062945

opennessfirstdifflat | -.0000136 .0002203 -0.06 0.951 -.0004454 .0004181

opennessseconddiff | .0090626 .0059934 1.51 0.131 -.0026843 .0208095

opennessseconddifflat | -.000418 .0001929 -2.17 0.030 -.000796 -.00004

---------------------------------------------------------------------------------------

Consumption PSV, Total Crops, 1992 – 2010

SAR with spatial fixed-effects Number of obs = 2397

Group variable: id Number of groups = 141

Time variable: year Panel length = 17

R-sq: within = 0.0047

between = 0.2259

overall = 0.0010

Mean of fixed-effects = 0.0008

Log-likelihood = 7350.7091

---------------------------------------------------------------------------------------

ydiffpsvc | Coef. Std. Err. z P>|z| [95% Conf. Interval]

----------------------+----------------------------------------------------------------

Main |

gdpcfirstdiff | .0149422 .0095491 1.56 0.118 -.0037737 .0336581

gdpcfirstdifflat | -.0007804 .0003123 -2.50 0.012 -.0013925 -.0001683

gdpcseconddiff | -.0014093 .0092625 -0.15 0.879 -.0195634 .0167448

gdpcseconddifflat | -.0000311 .0002928 -0.11 0.915 -.0006049 .0005426

opennessfirstdiff | -.0005486 .003215 -0.17 0.865 -.00685 .0057527

opennessfirstdifflat | .0000741 .0001178 0.63 0.529 -.0001568 .0003051

opennessseconddiff | .0012312 .0031125 0.40 0.692 -.0048691 .0073315

opennessseconddifflat | -.0000269 .0001015 -0.27 0.791 -.0002258 .000172

----------------------+----------------------------------------------------------------

Spatial |

rho | .0963287 .0734147 1.31 0.189 -.0475614 .2402189

----------------------+----------------------------------------------------------------

Variance |

sigma2_e | .000127 3.67e-06 34.61 0.000 .0001198 .0001342

----------------------+----------------------------------------------------------------

Direct |

gdpcfirstdiff | .0147979 .0081011 1.83 0.068 -.0010799 .0306757

gdpcfirstdifflat | -.0007622 .0003112 -2.45 0.014 -.0013722 -.0001522

gdpcseconddiff | -.00082 .0100298 -0.08 0.935 -.0204781 .0188381

gdpcseconddifflat | -.0000518 .000313 -0.17 0.869 -.0006654 .0005618

opennessfirstdiff | .0001077 .0031235 0.03 0.972 -.0060142 .0062296

opennessfirstdifflat | .0000676 .0001195 0.57 0.571 -.0001665 .0003017

opennessseconddiff | .0014744 .00323 0.46 0.648 -.0048563 .0078051

opennessseconddifflat | -.0000347 .0001026 -0.34 0.735 -.0002357 .0001663

----------------------+----------------------------------------------------------------

Indirect |

gdpcfirstdiff | .0014976 .00154 0.97 0.331 -.0015207 .0045158

gdpcfirstdifflat | -.0000795 .0000684 -1.16 0.245 -.0002136 .0000545

gdpcseconddiff | 3.67e-06 .0013008 0.00 0.998 -.0025458 .0025531

gdpcseconddifflat | -7.52e-06 .0000378 -0.20 0.842 -.0000816 .0000665

opennessfirstdiff | 9.26e-06 .0004177 0.02 0.982 -.0008094 .0008279

opennessfirstdifflat | 7.16e-06 .0000168 0.43 0.670 -.0000258 .0000401

opennessseconddiff | .0001754 .0004236 0.41 0.679 -.0006548 .0010056

opennessseconddifflat | -3.63e-06 .0000139 -0.26 0.794 -.0000309 .0000237

----------------------+----------------------------------------------------------------

Total |

gdpcfirstdiff | .0162955 .0090607 1.80 0.072 -.0014631 .0340542

gdpcfirstdifflat | -.0008417 .0003458 -2.43 0.015 -.0015195 -.000164

gdpcseconddiff | -.0008163 .0111304 -0.07 0.942 -.0226316 .0209989

gdpcseconddifflat | -.0000593 .0003448 -0.17 0.863 -.0007351 .0006165

opennessfirstdiff | .000117 .0034851 0.03 0.973 -.0067136 .0069476

opennessfirstdifflat | .0000748 .0001334 0.56 0.575 -.0001867 .0003363

opennessseconddiff | .0016498 .0035464 0.47 0.642 -.005301 .0086007

opennessseconddifflat | -.0000383 .0001134 -0.34 0.735 -.0002606 .000184

---------------------------------------------------------------------------------------

Consumption SR, Total Crops, 1992 – 2010

SAR with spatial fixed-effects Number of obs = 2397

Group variable: id Number of groups = 141

Time variable: year Panel length = 17

R-sq: within = 0.0526

between = 0.0110

overall = 0.0075

Mean of fixed-effects = 0.2478

Log-likelihood = 2463.9280

---------------------------------------------------------------------------------------

ydiffsrc | Coef. Std. Err. z P>|z| [95% Conf. Interval]

----------------------+----------------------------------------------------------------

Main |

gdpcfirstdiff | .1472452 .073083 2.01 0.044 .0040051 .2904853

gdpcfirstdifflat | -.0002625 .0023801 -0.11 0.912 -.0049275 .0044024

gdpcseconddiff | .1308821 .0707887 1.85 0.064 -.0078612 .2696255

gdpcseconddifflat | .0034147 .0022345 1.53 0.126 -.0009648 .0077942

opennessfirstdiff | .0089166 .0245324 0.36 0.716 -.039166 .0569993

opennessfirstdifflat | .0006831 .0008988 0.76 0.447 -.0010786 .0024448

opennessseconddiff | -.009141 .0237106 -0.39 0.700 -.055613 .0373309

opennessseconddifflat | .0007666 .0007743 0.99 0.322 -.000751 .0022841

----------------------+----------------------------------------------------------------

Spatial |

rho | .5704101 .0437853 13.03 0.000 .4845925 .6562278

----------------------+----------------------------------------------------------------

Variance |

sigma2_e | .0073941 .0002143 34.50 0.000 .0069741 .0078142

----------------------+----------------------------------------------------------------

Direct |

gdpcfirstdiff | .1483492 .0629068 2.36 0.018 .0250542 .2716442

gdpcfirstdifflat | -.0001238 .002407 -0.05 0.959 -.0048415 .0045939

gdpcseconddiff | .1375364 .0778787 1.77 0.077 -.0151031 .290176

gdpcseconddifflat | .0033083 .0024222 1.37 0.172 -.0014392 .0080557

opennessfirstdiff | .0141403 .0242055 0.58 0.559 -.0333017 .0615822

opennessfirstdifflat | .0006429 .0009256 0.69 0.487 -.0011712 .0024571

opennessseconddiff | -.0073808 .0249825 -0.30 0.768 -.0563457 .0415841

opennessseconddifflat | .0007181 .0007945 0.90 0.366 -.0008391 .0022754

----------------------+----------------------------------------------------------------

Indirect |

gdpcfirstdiff | .1897358 .0851019 2.23 0.026 .0229392 .3565324

gdpcfirstdifflat | -.0001777 .0030799 -0.06 0.954 -.0062141 .0058587

gdpcseconddiff | .1783874 .1071374 1.67 0.096 -.0315981 .3883729

gdpcseconddifflat | .0042442 .0031582 1.34 0.179 -.0019457 .0104342

opennessfirstdiff | .0182236 .0319859 0.57 0.569 -.0444677 .0809149

opennessfirstdifflat | .0008264 .001225 0.67 0.500 -.0015746 .0032273

opennessseconddiff | -.0085758 .0315888 -0.27 0.786 -.0704887 .0533371

opennessseconddifflat | .0009158 .0010437 0.88 0.380 -.0011299 .0029615

----------------------+----------------------------------------------------------------

Total |

gdpcfirstdiff | .338085 .1453633 2.33 0.020 .0531782 .6229918

gdpcfirstdifflat | -.0003015 .005468 -0.06 0.956 -.0110185 .0104156

gdpcseconddiff | .3159239 .1829199 1.73 0.084 -.0425926 .6744403

gdpcseconddifflat | .0075525 .0055261 1.37 0.172 -.0032785 .0183834

opennessfirstdiff | .0323639 .0559996 0.58 0.563 -.0773934 .1421211

opennessfirstdifflat | .0014693 .0021419 0.69 0.493 -.0027288 .0056674

opennessseconddiff | -.0159566 .0564 -0.28 0.777 -.1264986 .0945854

opennessseconddifflat | .0016339 .0018292 0.89 0.372 -.0019512 .0052191

---------------------------------------------------------------------------------------

Consumption E, Total Crops, 1992 – 2010

SAR with spatial fixed-effects Number of obs = 2397

Group variable: id Number of groups = 141

Time variable: year Panel length = 17

R-sq: within = 0.0028

between = 0.0207

overall = 0.0022

Mean of fixed-effects = -0.0005

Log-likelihood = 4088.9435

---------------------------------------------------------------------------------------

ydiffshc | Coef. Std. Err. z P>|z| [95% Conf. Interval]

----------------------+----------------------------------------------------------------

Main |

gdpcfirstdiff | -.0146256 .0372401 -0.39 0.695 -.0876148 .0583636

gdpcfirstdifflat | .0008192 .0012158 0.67 0.500 -.0015636 .0032021

gdpcseconddiff | .0222267 .0361453 0.61 0.539 -.0486168 .0930703

gdpcseconddifflat | -.0007959 .001142 -0.70 0.486 -.0030341 .0014423

opennessfirstdiff | .0108835 .0125534 0.87 0.386 -.0137206 .0354876

opennessfirstdifflat | -.0008284 .0004599 -1.80 0.072 -.0017297 .0000729

opennessseconddiff | .017696 .012112 1.46 0.144 -.0060431 .0414351

opennessseconddifflat | -.0006329 .0003957 -1.60 0.110 -.0014084 .0001426

----------------------+----------------------------------------------------------------

Spatial |

rho | .0798061 .0720759 1.11 0.268 -.0614601 .2210723

----------------------+----------------------------------------------------------------

Variance |

sigma2_e | .0019308 .0000558 34.62 0.000 .0018215 .0020401

----------------------+----------------------------------------------------------------

Direct |

gdpcfirstdiff | -.0152191 .031595 -0.48 0.630 -.0771442 .0467059

gdpcfirstdifflat | .0008915 .0012113 0.74 0.462 -.0014826 .0032656

gdpcseconddiff | .0245445 .0391455 0.63 0.531 -.0521792 .1012682

gdpcseconddifflat | -.0008767 .0012211 -0.72 0.473 -.00327 .0015166

opennessfirstdiff | .0134485 .0122021 1.10 0.270 -.0104672 .0373641

opennessfirstdifflat | -.0008543 .0004665 -1.83 0.067 -.0017686 .0000599

opennessseconddiff | .0186553 .0125755 1.48 0.138 -.0059922 .0433027

opennessseconddifflat | -.0006634 .0003997 -1.66 0.097 -.0014469 .00012

----------------------+----------------------------------------------------------------

Indirect |

gdpcfirstdiff | -.0015206 .0049023 -0.31 0.756 -.011129 .0080878

gdpcfirstdifflat | .0000724 .0001565 0.46 0.644 -.0002344 .0003792

gdpcseconddiff | .0023014 .0047756 0.48 0.630 -.0070587 .0116615

gdpcseconddifflat | -.0000784 .0001443 -0.54 0.587 -.0003613 .0002044

opennessfirstdiff | .0011794 .0017413 0.68 0.498 -.0022335 .0045923

opennessfirstdifflat | -.0000751 .0000869 -0.86 0.387 -.0002455 .0000952

opennessseconddiff | .0017233 .0022224 0.78 0.438 -.0026324 .0060791

opennessseconddifflat | -.0000574 .0000762 -0.75 0.451 -.0002067 .000092

----------------------+----------------------------------------------------------------

Total |

gdpcfirstdiff | -.0167397 .0350362 -0.48 0.633 -.0854094 .0519301

gdpcfirstdifflat | .0009639 .0013075 0.74 0.461 -.0015988 .0035266

gdpcseconddiff | .0268459 .0428494 0.63 0.531 -.0571375 .1108293

gdpcseconddifflat | -.0009551 .0013228 -0.72 0.470 -.0035479 .0016376

opennessfirstdiff | .0146278 .0134112 1.09 0.275 -.0116577 .0409133

opennessfirstdifflat | -.0009295 .0005187 -1.79 0.073 -.0019461 .0000871

opennessseconddiff | .0203786 .0138056 1.48 0.140 -.0066798 .047437

opennessseconddifflat | -.0007208 .0004404 -1.64 0.102 -.0015841 .0001424

---------------------------------------------------------------------------------------

**SAR estimates of model (2) with contemporaneous and once and twice lagged changes in independent variables (summarized in S2 Table)**

Production PSV, Total Crops, 1992 – 2010

SAR with spatial fixed-effects Number of obs = 2256

Group variable: id Number of groups = 141

Time variable: year Panel length = 16

R-sq: within = 0.0023

between = 0.0356

overall = 0.0031

Mean of fixed-effects = 0.0003

Log-likelihood = 9305.3537

---------------------------------------------------------------------------------------

ydiffpsvp | Coef. Std. Err. z P>|z| [95% Conf. Interval]

----------------------+----------------------------------------------------------------

Main |

gdpcfirstdiff | -.001268 .0042257 -0.30 0.764 -.0095503 .0070143

gdpcfirstdifflat | .0000856 .0001365 0.63 0.531 -.000182 .0003532

gdpcseconddiff | -.0009974 .0034197 -0.29 0.771 -.0076999 .0057052

gdpcseconddifflat | 4.87e-07 .0001169 0.00 0.997 -.0002287 .0002297

gdpcthirddiff | .0006207 .0033572 0.18 0.853 -.0059594 .0072008

gdpcthirddifflat | -.0000998 .0001119 -0.89 0.372 -.000319 .0001195

opennessfirstdiff | -.0009903 .0012785 -0.77 0.439 -.0034962 .0015156

opennessfirstdifflat | .0000426 .000048 0.89 0.375 -.0000515 .0001366

opennessseconddiff | -.0011555 .0012105 -0.95 0.340 -.0035281 .0012171

opennessseconddifflat | .0000452 .0000427 1.06 0.290 -.0000386 .000129

opennessthirddiff | -.001596 .0011675 -1.37 0.172 -.0038842 .0006921

opennessthirddifflat | .0000388 .0000378 1.02 0.305 -.0000354 .0001129

----------------------+----------------------------------------------------------------

Spatial |

rho | -.1507786 .0833878 -1.81 0.071 -.3142157 .0126584

----------------------+----------------------------------------------------------------

Variance |

sigma2_e | .0000153 4.56e-07 33.57 0.000 .0000144 .0000162

----------------------+----------------------------------------------------------------

Direct |

gdpcfirstdiff | -.0013361 .0035871 -0.37 0.710 -.0083668 .0056946

gdpcfirstdifflat | .0000934 .0001341 0.70 0.486 -.0001694 .0003562

gdpcseconddiff | -.0007863 .0037061 -0.21 0.832 -.00805 .0064775

gdpcseconddifflat | -6.92e-06 .0001238 -0.06 0.955 -.0002495 .0002356

gdpcthirddiff | .0012997 .0032389 0.40 0.688 -.0050484 .0076478

gdpcthirddifflat | -.0001054 .000112 -0.94 0.347 -.0003249 .0001141

opennessfirstdiff | -.0009853 .0013939 -0.71 0.480 -.0037173 .0017466

opennessfirstdifflat | .0000411 .0000488 0.84 0.400 -.0000546 .0001369

opennessseconddiff | -.0012671 .0012201 -1.04 0.299 -.0036585 .0011244

opennessseconddifflat | .0000481 .0000386 1.25 0.212 -.0000275 .0001237

opennessthirddiff | -.0015479 .0011517 -1.34 0.179 -.0038052 .0007095

opennessthirddifflat | .0000358 .0000382 0.94 0.349 -.0000391 .0001107

----------------------+----------------------------------------------------------------

Indirect |

gdpcfirstdiff | .0001792 .0005481 0.33 0.744 -.000895 .0012534

gdpcfirstdifflat | -.000011 .0000196 -0.56 0.576 -.0000494 .0000275

gdpcseconddiff | .0000848 .0005504 0.15 0.878 -.0009939 .0011635

gdpcseconddifflat | 5.88e-07 .0000185 0.03 0.975 -.0000358 .0000369

gdpcthirddiff | -.0001519 .0004489 -0.34 0.735 -.0010317 .000728

gdpcthirddifflat | .0000134 .0000172 0.78 0.437 -.0000204 .0000471

opennessfirstdiff | .0001235 .0002214 0.56 0.577 -.0003104 .0005575

opennessfirstdifflat | -4.97e-06 7.22e-06 -0.69 0.492 -.0000191 9.19e-06

opennessseconddiff | .0001611 .0001984 0.81 0.417 -.0002278 .00055

opennessseconddifflat | -5.94e-06 6.74e-06 -0.88 0.378 -.0000192 7.27e-06

opennessthirddiff | .0002031 .0001882 1.08 0.281 -.0001658 .0005719

opennessthirddifflat | -5.10e-06 5.98e-06 -0.85 0.394 -.0000168 6.62e-06

----------------------+----------------------------------------------------------------

Total |

gdpcfirstdiff | -.0011569 .0031394 -0.37 0.713 -.0073101 .0049963

gdpcfirstdifflat | .0000824 .0001199 0.69 0.492 -.0001526 .0003174

gdpcseconddiff | -.0007015 .0032517 -0.22 0.829 -.0070746 .0056716

gdpcseconddifflat | -6.34e-06 .0001086 -0.06 0.953 -.0002193 .0002066

gdpcthirddiff | .0011478 .002841 0.40 0.686 -.0044205 .0067161

gdpcthirddifflat | -.000092 .0000981 -0.94 0.348 -.0002843 .0001003

opennessfirstdiff | -.0008618 .0012105 -0.71 0.476 -.0032342 .0015107

opennessfirstdifflat | .0000362 .0000432 0.84 0.402 -.0000485 .0001208

opennessseconddiff | -.001106 .0010733 -1.03 0.303 -.0032096 .0009976

opennessseconddifflat | .0000422 .0000341 1.24 0.217 -.0000247 .000109

opennessthirddiff | -.0013448 .001013 -1.33 0.184 -.0033302 .0006406

opennessthirddifflat | .0000307 .0000331 0.93 0.354 -.0000343 .0000957

---------------------------------------------------------------------------------------

Production SR, Total Crops, 1992 – 2010

SAR with spatial fixed-effects Number of obs = 2256

Group variable: id Number of groups = 141

Time variable: year Panel length = 16

R-sq: within = 0.0033

between = 0.0221

overall = 0.0011

Mean of fixed-effects = 0.0032

Log-likelihood = 5469.3143

---------------------------------------------------------------------------------------

ydiffsrp | Coef. Std. Err. z P>|z| [95% Conf. Interval]

----------------------+----------------------------------------------------------------

Main |

gdpcfirstdiff | -.0222295 .0231343 -0.96 0.337 -.067572 .0231129

gdpcfirstdifflat | .0000658 .0007477 0.09 0.930 -.0013997 .0015313

gdpcseconddiff | -.0070725 .0187066 -0.38 0.705 -.0437368 .0295918

gdpcseconddifflat | .0002975 .0006393 0.47 0.642 -.0009554 .0015505

gdpcthirddiff | -.0164561 .0183945 -0.89 0.371 -.0525088 .0195965

gdpcthirddifflat | .0001846 .0006119 0.30 0.763 -.0010147 .0013839

opennessfirstdiff | .0035786 .0069934 0.51 0.609 -.0101282 .0172854

opennessfirstdifflat | -.0000417 .0002624 -0.16 0.874 -.000556 .0004726

opennessseconddiff | -.0037103 .0066221 -0.56 0.575 -.0166894 .0092688

opennessseconddifflat | .000289 .0002341 1.23 0.217 -.0001697 .0007477

opennessthirddiff | .0017285 .00636 0.27 0.786 -.0107369 .0141939

opennessthirddifflat | -.0001555 .0002064 -0.75 0.451 -.0005601 .000249

----------------------+----------------------------------------------------------------

Spatial |

rho | .2550985 .0670491 3.80 0.000 .1236847 .3865123

----------------------+----------------------------------------------------------------

Variance |

sigma2_e | .0004579 .0000136 33.56 0.000 .0004311 .0004846

----------------------+----------------------------------------------------------------

Direct |

gdpcfirstdiff | -.0226512 .0196678 -1.15 0.249 -.0611995 .015897

gdpcfirstdifflat | .0001082 .0007359 0.15 0.883 -.001334 .0015505

gdpcseconddiff | -.0059328 .0203076 -0.29 0.770 -.0457351 .0338694

gdpcseconddifflat | .0002577 .0006777 0.38 0.704 -.0010706 .001586

gdpcthirddiff | -.0127744 .0177675 -0.72 0.472 -.0475982 .0220493

gdpcthirddifflat | .0001546 .0006132 0.25 0.801 -.0010474 .0013565

opennessfirstdiff | .0036171 .0076383 0.47 0.636 -.0113537 .0185879

opennessfirstdifflat | -.0000497 .0002677 -0.19 0.853 -.0005744 .000475

opennessseconddiff | -.004331 .0066852 -0.65 0.517 -.0174337 .0087717

opennessseconddifflat | .0003056 .0002115 1.44 0.149 -.000109 .0007202

opennessthirddiff | .0019979 .0062836 0.32 0.751 -.0103176 .0143134

opennessthirddifflat | -.0001723 .0002088 -0.83 0.409 -.0005815 .0002369

----------------------+----------------------------------------------------------------

Indirect |

gdpcfirstdiff | -.0077162 .007899 -0.98 0.329 -.0231981 .0077656

gdpcfirstdifflat | .000043 .0002905 0.15 0.882 -.0005264 .0006123

gdpcseconddiff | -.0022955 .0078493 -0.29 0.770 -.0176799 .013089

gdpcseconddifflat | .0000907 .0002644 0.34 0.732 -.0004275 .0006089

gdpcthirddiff | -.0042477 .0065039 -0.65 0.514 -.0169952 .0084998

gdpcthirddifflat | .0000487 .0002222 0.22 0.827 -.0003869 .0004842

opennessfirstdiff | .0012158 .0027605 0.44 0.660 -.0041947 .0066263

opennessfirstdifflat | -.0000141 .0001026 -0.14 0.891 -.0002151 .000187

opennessseconddiff | -.0015729 .002611 -0.60 0.547 -.0066904 .0035447

opennessseconddifflat | .0001103 .0000953 1.16 0.247 -.0000764 .000297

opennessthirddiff | .0006797 .0024177 0.28 0.779 -.0040589 .0054182

opennessthirddifflat | -.0000632 .0000848 -0.75 0.456 -.0002294 .000103

----------------------+----------------------------------------------------------------

Total |

gdpcfirstdiff | -.0303674 .0266713 -1.14 0.255 -.0826423 .0219074

gdpcfirstdifflat | .0001512 .0010135 0.15 0.881 -.0018352 .0021377

gdpcseconddiff | -.0082283 .0276771 -0.30 0.766 -.0624745 .0460178

gdpcseconddifflat | .0003484 .0009236 0.38 0.706 -.0014619 .0021587

gdpcthirddiff | -.0170221 .0238997 -0.71 0.476 -.0638648 .0298205

gdpcthirddifflat | .0002032 .0008256 0.25 0.806 -.001415 .0018215

opennessfirstdiff | .0048329 .0102227 0.47 0.636 -.0152031 .0248689

opennessfirstdifflat | -.0000638 .0003654 -0.17 0.861 -.0007799 .0006523

opennessseconddiff | -.0059039 .0091266 -0.65 0.518 -.0237918 .011984

opennessseconddifflat | .0004159 .0002952 1.41 0.159 -.0001627 .0009945

opennessthirddiff | .0026776 .0086094 0.31 0.756 -.0141966 .0195518

opennessthirddifflat | -.0002355 .0002882 -0.82 0.414 -.0008004 .0003294

---------------------------------------------------------------------------------------

Production E, Total Crops, 1992 – 2010

SAR with spatial fixed-effects Number of obs = 2256

Group variable: id Number of groups = 141

Time variable: year Panel length = 16

R-sq: within = 0.0081

between = 0.0196

overall = 0.0048

Mean of fixed-effects = 0.0010

Log-likelihood = 5813.0856

---------------------------------------------------------------------------------------

ydiffshp | Coef. Std. Err. z P>|z| [95% Conf. Interval]

----------------------+----------------------------------------------------------------

Main |

gdpcfirstdiff | -.0341001 .0198931 -1.71 0.086 -.0730899 .0048897

gdpcfirstdifflat | .001753 .0006431 2.73 0.006 .0004925 .0030135

gdpcseconddiff | -.0173878 .0160758 -1.08 0.279 -.0488957 .0141201

gdpcseconddifflat | .0001203 .0005495 0.22 0.827 -.0009566 .0011972

gdpcthirddiff | .0128669 .015789 0.81 0.415 -.0180789 .0438127

gdpcthirddifflat | -.0005935 .0005256 -1.13 0.259 -.0016237 .0004367

opennessfirstdiff | -.0008737 .0060134 -0.15 0.884 -.0126598 .0109123

opennessfirstdifflat | -.0000795 .0002256 -0.35 0.725 -.0005217 .0003627

opennessseconddiff | .0092985 .0056893 1.63 0.102 -.0018524 .0204494

opennessseconddifflat | -.0004308 .0002009 -2.14 0.032 -.0008246 -.000037

opennessthirddiff | .0040621 .0054724 0.74 0.458 -.0066636 .0147878

opennessthirddifflat | -.0000105 .0001777 -0.06 0.953 -.0003587 .0003378

----------------------+----------------------------------------------------------------

Spatial |

rho | .1043885 .0737073 1.42 0.157 -.0400752 .2488523

----------------------+----------------------------------------------------------------

Variance |

sigma2_e | .0003383 .0000101 33.58 0.000 .0003185 .000358

----------------------+----------------------------------------------------------------

Direct |

gdpcfirstdiff | -.0344328 .0168787 -2.04 0.041 -.0675144 -.0013513

gdpcfirstdifflat | .0017902 .0006316 2.83 0.005 .0005524 .0030281

gdpcseconddiff | -.0164029 .0174169 -0.94 0.346 -.0505394 .0177335

gdpcseconddifflat | .0000855 .0005813 0.15 0.883 -.0010538 .0012248

gdpcthirddiff | .0160647 .0152271 1.06 0.291 -.0137799 .0459092

gdpcthirddifflat | -.0006199 .0005259 -1.18 0.239 -.0016507 .0004109

opennessfirstdiff | -.0008496 .0065558 -0.13 0.897 -.0136987 .0119995

opennessfirstdifflat | -.0000863 .0002297 -0.38 0.707 -.0005365 .000364

opennessseconddiff | .0087814 .0057317 1.53 0.126 -.0024525 .0200153

opennessseconddifflat | -.0004176 .0001812 -2.31 0.021 -.0007727 -.0000625

opennessthirddiff | .0042908 .0053949 0.80 0.426 -.006283 .0148646

opennessthirddifflat | -.0000245 .0001793 -0.14 0.892 -.0003759 .000327

----------------------+----------------------------------------------------------------

Indirect |

gdpcfirstdiff | -.004062 .0043678 -0.93 0.352 -.0126228 .0044987

gdpcfirstdifflat | .0002216 .0002187 1.01 0.311 -.0002071 .0006504

gdpcseconddiff | -.0021588 .0035532 -0.61 0.543 -.009123 .0048053

gdpcseconddifflat | .0000117 .0001008 0.12 0.908 -.000186 .0002093

gdpcthirddiff | .0020595 .002741 0.75 0.452 -.0033127 .0074318

gdpcthirddifflat | -.0000776 .0000979 -0.79 0.428 -.0002693 .0001142

opennessfirstdiff | -.0001456 .0009861 -0.15 0.883 -.0020784 .0017872

opennessfirstdifflat | -7.80e-06 .0000386 -0.20 0.840 -.0000834 .0000678

opennessseconddiff | .0010344 .001235 0.84 0.402 -.0013861 .0034549

opennessseconddifflat | -.0000492 .0000456 -1.08 0.280 -.0001386 .0000401

opennessthirddiff | .0004932 .0009571 0.52 0.606 -.0013827 .0023691

opennessthirddifflat | -4.30e-06 .0000279 -0.15 0.877 -.0000589 .0000503

----------------------+----------------------------------------------------------------

Total |

gdpcfirstdiff | -.0384949 .019066 -2.02 0.043 -.0758636 -.0011261

gdpcfirstdifflat | .0020119 .0007517 2.68 0.007 .0005386 .0034852

gdpcseconddiff | -.0185618 .0198924 -0.93 0.351 -.0575503 .0204267

gdpcseconddifflat | .0000972 .0006577 0.15 0.883 -.0011918 .0013862

gdpcthirddiff | .0181242 .0172749 1.05 0.294 -.015734 .0519824

gdpcthirddifflat | -.0006974 .0005943 -1.17 0.241 -.0018622 .0004674

opennessfirstdiff | -.0009952 .007324 -0.14 0.892 -.0153499 .0133595

opennessfirstdifflat | -.0000941 .0002601 -0.36 0.718 -.0006039 .0004158

opennessseconddiff | .0098158 .0064583 1.52 0.129 -.0028421 .0224738

opennessseconddifflat | -.0004668 .0002029 -2.30 0.021 -.0008645 -.0000691

opennessthirddiff | .004784 .0061274 0.78 0.435 -.0072255 .0167936

opennessthirddifflat | -.0000288 .0002029 -0.14 0.887 -.0004264 .0003689

---------------------------------------------------------------------------------------

Consumption PSV, Total Crops, 1992 – 2010

SAR with spatial fixed-effects Number of obs = 2256

Group variable: id Number of groups = 141

Time variable: year Panel length = 16

R-sq: within = 0.0032

between = 0.1403

overall = 0.0011

Mean of fixed-effects = 0.0006

Log-likelihood = 6942.2291

---------------------------------------------------------------------------------------

ydiffpsvc | Coef. Std. Err. z P>|z| [95% Conf. Interval]

----------------------+----------------------------------------------------------------

Main |

gdpcfirstdiff | .0096736 .0120508 0.80 0.422 -.0139455 .0332927

gdpcfirstdifflat | -.0003573 .0003898 -0.92 0.359 -.0011212 .0004066

gdpcseconddiff | -.0013868 .0097509 -0.14 0.887 -.0204982 .0177246

gdpcseconddifflat | .0001522 .0003332 0.46 0.648 -.0005009 .0008052

gdpcthirddiff | .0023463 .0095725 0.25 0.806 -.0164154 .021108

gdpcthirddifflat | -.0003279 .0003188 -1.03 0.304 -.0009527 .0002969

opennessfirstdiff | -.0021573 .0036445 -0.59 0.554 -.0093003 .0049858

opennessfirstdifflat | .000147 .0001367 1.07 0.282 -.000121 .000415

opennessseconddiff | .0040567 .0034516 1.18 0.240 -.0027083 .0108217

opennessseconddifflat | -.0001565 .0001218 -1.28 0.199 -.0003952 .0000823

opennessthirddiff | .0022209 .0033171 0.67 0.503 -.0042805 .0087223

opennessthirddifflat | -.0000587 .0001077 -0.54 0.586 -.0002697 .0001523

----------------------+----------------------------------------------------------------

Spatial |

rho | .0805045 .0758947 1.06 0.289 -.0682463 .2292554

----------------------+----------------------------------------------------------------

Variance |

sigma2_e | .0001243 3.70e-06 33.58 0.000 .0001171 .0001316

----------------------+----------------------------------------------------------------

Direct |

gdpcfirstdiff | .0094883 .010224 0.93 0.353 -.0105504 .0295269

gdpcfirstdifflat | -.0003354 .0003827 -0.88 0.381 -.0010855 .0004147

gdpcseconddiff | -.0007853 .0105613 -0.07 0.941 -.0214852 .0199145

gdpcseconddifflat | .0001311 .0003524 0.37 0.710 -.0005597 .0008218

gdpcthirddiff | .0042811 .0092297 0.46 0.643 -.0138088 .022371

gdpcthirddifflat | -.0003438 .0003188 -1.08 0.281 -.0009686 .000281

opennessfirstdiff | -.0021425 .0039725 -0.54 0.590 -.0099285 .0056434

opennessfirstdifflat | .0001429 .0001392 1.03 0.304 -.0001299 .0004158

opennessseconddiff | .0037411 .0034772 1.08 0.282 -.0030742 .0105564

opennessseconddifflat | -.0001484 .0001098 -1.35 0.177 -.0003636 .0000668

opennessthirddiff | .0023622 .0032709 0.72 0.470 -.0040487 .0087731

opennessthirddifflat | -.0000673 .0001087 -0.62 0.536 -.0002803 .0001457

----------------------+----------------------------------------------------------------

Indirect |

gdpcfirstdiff | .0009336 .0016999 0.55 0.583 -.0023981 .0042654

gdpcfirstdifflat | -.0000257 .0000608 -0.42 0.672 -.0001448 .0000934

gdpcseconddiff | -.0001967 .0015655 -0.13 0.900 -.0032649 .0028715

gdpcseconddifflat | .000012 .0000568 0.21 0.833 -.0000994 .0001233

gdpcthirddiff | .0004747 .0012441 0.38 0.703 -.0019638 .0029131

gdpcthirddifflat | -.0000339 .0000535 -0.63 0.526 -.0001388 .0000709

opennessfirstdiff | -.000212 .0005598 -0.38 0.705 -.0013092 .0008852

opennessfirstdifflat | .0000148 .0000248 0.60 0.550 -.0000339 .0000635

opennessseconddiff | .0003256 .0006194 0.53 0.599 -.0008884 .0015396

opennessseconddifflat | -.0000132 .0000198 -0.67 0.505 -.0000519 .0000256

opennessthirddiff | .0002368 .0005489 0.43 0.666 -.000839 .0013126

opennessthirddifflat | -8.33e-06 .0000177 -0.47 0.638 -.000043 .0000263

----------------------+----------------------------------------------------------------

Total |

gdpcfirstdiff | .0104219 .0112999 0.92 0.356 -.0117254 .0325692

gdpcfirstdifflat | -.0003612 .0004236 -0.85 0.394 -.0011914 .0004691

gdpcseconddiff | -.000982 .0116589 -0.08 0.933 -.0238331 .0218691

gdpcseconddifflat | .0001431 .0003907 0.37 0.714 -.0006228 .0009089

gdpcthirddiff | .0047558 .0101887 0.47 0.641 -.0152137 .0247253

gdpcthirddifflat | -.0003778 .0003531 -1.07 0.285 -.0010698 .0003143

opennessfirstdiff | -.0023545 .0043307 -0.54 0.587 -.0108425 .0061334

opennessfirstdifflat | .0001578 .0001554 1.02 0.310 -.0001467 .0004623

opennessseconddiff | .0040667 .0038158 1.07 0.287 -.0034121 .0115456

opennessseconddifflat | -.0001616 .0001202 -1.34 0.179 -.0003971 .000074

opennessthirddiff | .002599 .0036627 0.71 0.478 -.0045799 .0097778

opennessthirddifflat | -.0000756 .0001218 -0.62 0.535 -.0003142 .000163

---------------------------------------------------------------------------------------

Consumption SR, Total Crops, 1992 – 2010

SAR with spatial fixed-effects Number of obs = 2256

Group variable: id Number of groups = 141

Time variable: year Panel length = 16

R-sq: within = 0.0533

between = 0.0119

overall = 0.0084

Mean of fixed-effects = 0.2612

Log-likelihood = 2452.4569

---------------------------------------------------------------------------------------

ydiffsrc | Coef. Std. Err. z P>|z| [95% Conf. Interval]

----------------------+----------------------------------------------------------------

Main |

gdpcfirstdiff | .1210466 .0877549 1.38 0.168 -.0509498 .2930431

gdpcfirstdifflat | -.0013148 .0028318 -0.46 0.642 -.0068651 .0042354

gdpcseconddiff | .1344859 .0710687 1.89 0.058 -.0048062 .2737779

gdpcseconddifflat | .0016757 .0024239 0.69 0.489 -.003075 .0064265

gdpcthirddiff | .0745246 .0699574 1.07 0.287 -.0625893 .2116385

gdpcthirddifflat | .0026362 .0023173 1.14 0.255 -.0019057 .0071781

opennessfirstdiff | -.0062875 .026506 -0.24 0.812 -.0582382 .0456633

opennessfirstdifflat | .0011576 .0009946 1.16 0.244 -.0007917 .0031069

opennessseconddiff | -.0155905 .0250885 -0.62 0.534 -.064763 .0335821

opennessseconddifflat | .0012618 .000886 1.42 0.154 -.0004748 .0029984

opennessthirddiff | .0265769 .0241022 1.10 0.270 -.0206626 .0738164

opennessthirddifflat | -.0007307 .0007822 -0.93 0.350 -.0022639 .0008024

----------------------+----------------------------------------------------------------

Spatial |

rho | .5481703 .0480907 11.40 0.000 .4539142 .6424263

----------------------+----------------------------------------------------------------

Variance |

sigma2_e | .0065768 .0001965 33.47 0.000 .0061917 .0069619

----------------------+----------------------------------------------------------------

Direct |

gdpcfirstdiff | .1213653 .0754831 1.61 0.108 -.026579 .2693095

gdpcfirstdifflat | -.0011708 .0028197 -0.42 0.678 -.0066974 .0043558

gdpcseconddiff | .1408268 .0779833 1.81 0.071 -.0120177 .2936713

gdpcseconddifflat | .0015439 .0025998 0.59 0.553 -.0035516 .0066394

gdpcthirddiff | .0899448 .0683067 1.32 0.188 -.0439338 .2238234

gdpcthirddifflat | .0025622 .0023506 1.09 0.276 -.0020449 .0071693

opennessfirstdiff | -.0062089 .0292602 -0.21 0.832 -.0635578 .0511401

opennessfirstdifflat | .0011424 .0010268 1.11 0.266 -.0008701 .0031549

opennessseconddiff | -.01817 .0256218 -0.71 0.478 -.0683878 .0320478

opennessseconddifflat | .0013407 .0008102 1.65 0.098 -.0002473 .0029286

opennessthirddiff | .0279604 .024087 1.16 0.246 -.0192494 .0751701

opennessthirddifflat | -.0008033 .0008004 -1.00 0.316 -.0023721 .0007655

----------------------+----------------------------------------------------------------

Indirect |

gdpcfirstdiff | .1448333 .0972814 1.49 0.137 -.0458347 .3355013

gdpcfirstdifflat | -.0013256 .0035291 -0.38 0.707 -.0082425 .0055913

gdpcseconddiff | .16504 .0963782 1.71 0.087 -.0238577 .3539377

gdpcseconddifflat | .0018644 .0032921 0.57 0.571 -.004588 .0083169

gdpcthirddiff | .1068905 .0855336 1.25 0.211 -.0607523 .2745333

gdpcthirddifflat | .0030159 .0028764 1.05 0.294 -.0026218 .0086536

opennessfirstdiff | -.0075717 .034663 -0.22 0.827 -.0755101 .0603666

opennessfirstdifflat | .0013838 .0013203 1.05 0.295 -.001204 .0039716

opennessseconddiff | -.0217011 .0317227 -0.68 0.494 -.0838765 .0404743

opennessseconddifflat | .0016093 .001073 1.50 0.134 -.0004937 .0037122

opennessthirddiff | .0332323 .0305344 1.09 0.276 -.0266141 .0930788

opennessthirddifflat | -.0009835 .0010292 -0.96 0.339 -.0030007 .0010338

----------------------+----------------------------------------------------------------

Total |

gdpcfirstdiff | .2661986 .170012 1.57 0.117 -.0670188 .5994159

gdpcfirstdifflat | -.0024964 .0063135 -0.40 0.693 -.0148706 .0098777

gdpcseconddiff | .3058668 .1707723 1.79 0.073 -.0288407 .6405743

gdpcseconddifflat | .0034084 .0058371 0.58 0.559 -.0080322 .0148489

gdpcthirddiff | .1968353 .1521299 1.29 0.196 -.1013339 .4950045

gdpcthirddifflat | .0055781 .0051702 1.08 0.281 -.0045553 .0157114

opennessfirstdiff | -.0137806 .0636083 -0.22 0.828 -.1384505 .1108893

opennessfirstdifflat | .0025262 .0023236 1.09 0.277 -.0020279 .0070803

opennessseconddiff | -.0398711 .0569207 -0.70 0.484 -.1514337 .0716915

opennessseconddifflat | .0029499 .0018455 1.60 0.110 -.0006671 .0065669

opennessthirddiff | .0611927 .0539995 1.13 0.257 -.0446445 .1670299

opennessthirddifflat | -.0017868 .0018112 -0.99 0.324 -.0053366 .001763

---------------------------------------------------------------------------------------

Consumption E, Total Crops, 1992 – 2010

SAR with spatial fixed-effects Number of obs = 2256

Group variable: id Number of groups = 141

Time variable: year Panel length = 16

R-sq: within = 0.0077

between = 0.0000

overall = 0.0073

Mean of fixed-effects = -0.0008

Log-likelihood = 3908.6236

---------------------------------------------------------------------------------------

ydiffshc | Coef. Std. Err. z P>|z| [95% Conf. Interval]

----------------------+----------------------------------------------------------------

Main |

gdpcfirstdiff | .0067232 .0462367 0.15 0.884 -.0838991 .0973456

gdpcfirstdifflat | -.0000112 .0014936 -0.01 0.994 -.0029386 .0029163

gdpcseconddiff | -.0135438 .0374052 -0.36 0.717 -.0868567 .059769

gdpcseconddifflat | -.0011276 .0012785 -0.88 0.378 -.0036335 .0013783

gdpcthirddiff | .0188322 .0367562 0.51 0.608 -.0532086 .090873

gdpcthirddifflat | .0015724 .0012241 1.28 0.199 -.0008267 .0039716

opennessfirstdiff | .0207474 .0139827 1.48 0.138 -.0066581 .048153

opennessfirstdifflat | -.0008528 .0005247 -1.63 0.104 -.0018812 .0001756

opennessseconddiff | .0134718 .013238 1.02 0.309 -.0124741 .0394177

opennessseconddifflat | -.0003888 .0004678 -0.83 0.406 -.0013057 .0005281

opennessthirddiff | .0135652 .012721 1.07 0.286 -.0113676 .0384979

opennessthirddifflat | .0001598 .0004127 0.39 0.699 -.0006491 .0009687

----------------------+----------------------------------------------------------------

Spatial |

rho | .00907 .0768371 0.12 0.906 -.141528 .159668

----------------------+----------------------------------------------------------------

Variance |

sigma2_e | .0018309 .0000545 33.59 0.000 .001724 .0019377

----------------------+----------------------------------------------------------------

Direct |

gdpcfirstdiff | .0059963 .0392187 0.15 0.878 -.0708709 .0828635

gdpcfirstdifflat | .000073 .0014662 0.05 0.960 -.0028006 .0029467

gdpcseconddiff | -.0112301 .0405086 -0.28 0.782 -.0906255 .0681653

gdpcseconddifflat | -.001209 .0013521 -0.89 0.371 -.0038591 .0014411

gdpcthirddiff | .026258 .0354385 0.74 0.459 -.0432001 .0957162

gdpcthirddifflat | .0015121 .0012244 1.23 0.217 -.0008877 .0039118

opennessfirstdiff | .0208118 .015238 1.37 0.172 -.009054 .0506777

opennessfirstdifflat | -.0008688 .0005341 -1.63 0.104 -.0019155 .000178

opennessseconddiff | .0122609 .0133325 0.92 0.358 -.0138703 .0383922

opennessseconddifflat | -.0003576 .0004216 -0.85 0.396 -.0011839 .0004688

opennessthirddiff | .0140983 .0125348 1.12 0.261 -.0104695 .038666

opennessthirddifflat | .0001272 .0004164 0.31 0.760 -.0006889 .0009433

----------------------+----------------------------------------------------------------

Indirect |

gdpcfirstdiff | .0002846 .0036852 0.08 0.938 -.0069383 .0075076

gdpcfirstdifflat | .0000121 .000137 0.09 0.930 -.0002564 .0002807

gdpcseconddiff | -.0005399 .0042045 -0.13 0.898 -.0087805 .0077008

gdpcseconddifflat | -.0000129 .0001759 -0.07 0.942 -.0003576 .0003318

gdpcthirddiff | .0006352 .0032405 0.20 0.845 -.005716 .0069864

gdpcthirddifflat | .0000123 .0001655 0.07 0.941 -.0003121 .0003367

opennessfirstdiff | .0002632 .0022749 0.12 0.908 -.0041955 .0047219

opennessfirstdifflat | -8.76e-06 .0000914 -0.10 0.924 -.000188 .0001704

opennessseconddiff | .0001058 .0016183 0.07 0.948 -.0030661 .0032776

opennessseconddifflat | -1.12e-06 .0000449 -0.02 0.980 -.0000891 .0000868

opennessthirddiff | .0001602 .001622 0.10 0.921 -.0030189 .0033392

opennessthirddifflat | -2.02e-06 .0000314 -0.06 0.949 -.0000635 .0000595

----------------------+----------------------------------------------------------------

Total |

gdpcfirstdiff | .0062809 .0399709 0.16 0.875 -.0720605 .0846224

gdpcfirstdifflat | .0000851 .0015126 0.06 0.955 -.0028796 .0030499

gdpcseconddiff | -.01177 .0414597 -0.28 0.776 -.0930296 .0694896

gdpcseconddifflat | -.0012219 .0013828 -0.88 0.377 -.0039321 .0014883

gdpcthirddiff | .0268932 .0362489 0.74 0.458 -.0441534 .0979399

gdpcthirddifflat | .0015243 .0012366 1.23 0.218 -.0008994 .0039481

opennessfirstdiff | .021075 .0154022 1.37 0.171 -.0091127 .0512628

opennessfirstdifflat | -.0008775 .0005464 -1.61 0.108 -.0019484 .0001933

opennessseconddiff | .0123667 .0135519 0.91 0.361 -.0141945 .0389279

opennessseconddifflat | -.0003587 .000426 -0.84 0.400 -.0011936 .0004762

opennessthirddiff | .0142585 .0129173 1.10 0.270 -.011059 .0395759

opennessthirddifflat | .0001252 .0004247 0.29 0.768 -.0007071 .0009575

---------------------------------------------------------------------------------------

**SAR estimates of static model (3) (summarized in Table 3)**

Production PSV, Total Crops, 1992 – 2010

SAR with spatial fixed-effects Number of obs = 1008

Group variable: id Number of groups = 56

Time variable: year Panel length = 18

R-sq: within = 0.0244

between = 0.0013

overall = 0.0229

Mean of fixed-effects = 0.0005

Log-likelihood = 4237.6515

---------------------------------------------------------------------------------

ydiffpsvp | Coef. Std. Err. z P>|z| [95% Conf. Interval]

----------------+----------------------------------------------------------------

Main |

gdpcdiff | .0069725 .0069915 1.00 0.319 -.0067306 .0206756

gdpcdifflat | -.000159 .0001637 -0.97 0.331 -.0004798 .0001618

opennessdiff | -.0018443 .0019791 -0.93 0.351 -.0057232 .0020347

opennessdifflat | .0000964 .0000529 1.82 0.068 -7.25e-06 .0002

nradiff | -.0003775 .0022831 -0.17 0.869 -.0048523 .0040973

nradifflat | .0000168 .0000463 0.36 0.716 -.000074 .0001076

tbidiff | -.00018 .0010119 -0.18 0.859 -.0021633 .0018034

tbidifflat | 3.18e-06 .0000168 0.19 0.850 -.0000298 .0000361

----------------+----------------------------------------------------------------

Spatial |

rho | -.4244355 .1278295 -3.32 0.001 -.6749766 -.1738943

----------------+----------------------------------------------------------------

Variance |

sigma2_e | .000013 5.80e-07 22.37 0.000 .0000118 .0000141

----------------+----------------------------------------------------------------

Direct |

gdpcdiff | .0069104 .0059675 1.16 0.247 -.0047856 .0186064

gdpcdifflat | -.0001519 .0001565 -0.97 0.332 -.0004586 .0001548

opennessdiff | -.0017447 .0021474 -0.81 0.417 -.0059535 .0024641

opennessdifflat | .0000946 .0000564 1.68 0.094 -.000016 .0002051

nradiff | .0001099 .0021287 0.05 0.959 -.0040623 .004282

nradifflat | .0000113 .0000442 0.26 0.798 -.0000754 .000098

tbidiff | -.0001735 .0010766 -0.16 0.872 -.0022836 .0019367

tbidifflat | 3.07e-06 .0000179 0.17 0.864 -.000032 .0000381

----------------+----------------------------------------------------------------

Indirect |

gdpcdiff | -.0021209 .0018391 -1.15 0.249 -.0057256 .0014837

gdpcdifflat | .0000461 .0000493 0.94 0.349 -.0000505 .0001427

opennessdiff | .0005504 .0006883 0.80 0.424 -.0007987 .0018995

opennessdifflat | -.0000292 .0000193 -1.51 0.131 -.000067 8.66e-06

nradiff | -.0000273 .0006474 -0.04 0.966 -.0012962 .0012417

nradifflat | -3.70e-06 .0000135 -0.27 0.785 -.0000302 .0000228

tbidiff | .00006 .0003455 0.17 0.862 -.0006171 .0007371

tbidifflat | -1.05e-06 5.74e-06 -0.18 0.854 -.0000123 .0000102

----------------+----------------------------------------------------------------

Total |

gdpcdiff | .0047895 .004234 1.13 0.258 -.003509 .0130879

gdpcdifflat | -.0001058 .0001093 -0.97 0.333 -.0003199 .0001084

opennessdiff | -.0011943 .0014888 -0.80 0.422 -.0041123 .0017236

opennessdifflat | .0000654 .0000388 1.68 0.092 -.0000107 .0001415

nradiff | .0000826 .0014933 0.06 0.956 -.0028442 .0030093

nradifflat | 7.61e-06 .000031 0.25 0.806 -.0000531 .0000683

tbidiff | -.0001134 .0007391 -0.15 0.878 -.001562 .0013352

tbidifflat | 2.02e-06 .0000123 0.16 0.870 -.0000221 .0000261

---------------------------------------------------------------------------------

Production SR, Total Crops, 1992 – 2010

SAR with spatial fixed-effects Number of obs = 1008

Group variable: id Number of groups = 56

Time variable: year Panel length = 18

R-sq: within = 0.0040

between = 0.1192

overall = 0.0103

Mean of fixed-effects = 0.0041

Log-likelihood = 2396.6343

---------------------------------------------------------------------------------

ydiffsrp | Coef. Std. Err. z P>|z| [95% Conf. Interval]

----------------+----------------------------------------------------------------

Main |

gdpcdiff | -.0688558 .0435393 -1.58 0.114 -.1541914 .0164797

gdpcdifflat | .0011655 .0010194 1.14 0.253 -.0008325 .0031635

opennessdiff | .0034905 .0123255 0.28 0.777 -.0206671 .0276481

opennessdifflat | .0001177 .0003295 0.36 0.721 -.0005282 .0007635

nradiff | -.0029517 .0142184 -0.21 0.836 -.0308193 .0249159

nradifflat | .0000244 .0002884 0.08 0.932 -.0005409 .0005898

tbidiff | .0021783 .0063013 0.35 0.730 -.0101721 .0145287

tbidifflat | -.0000363 .0001047 -0.35 0.729 -.0002416 .0001689

----------------+----------------------------------------------------------------

Spatial |

rho | .1872662 .0896825 2.09 0.037 .0114917 .3630407

----------------+----------------------------------------------------------------

Variance |

sigma2_e | .0005031 .0000224 22.44 0.000 .0004592 .0005471

----------------+----------------------------------------------------------------

Direct |

gdpcdiff | -.0696814 .0370242 -1.88 0.060 -.1422474 .0028846

gdpcdifflat | .0012184 .0009697 1.26 0.209 -.0006821 .0031189

opennessdiff | .0042039 .0133018 0.32 0.752 -.0218672 .0302751

opennessdifflat | .0001021 .0003493 0.29 0.770 -.0005824 .0007866

nradiff | .0000813 .0131941 0.01 0.995 -.0257787 .0259414

nradifflat | -.0000106 .0002739 -0.04 0.969 -.0005474 .0005262

tbidiff | .0022323 .0066636 0.33 0.738 -.0108281 .0152927

tbidifflat | -.0000373 .0001107 -0.34 0.737 -.0002543 .0001798

----------------+----------------------------------------------------------------

Indirect |

gdpcdiff | -.0165649 .0162192 -1.02 0.307 -.0483539 .015224

gdpcdifflat | .0002817 .0003196 0.88 0.378 -.0003447 .000908

opennessdiff | .0011596 .0035408 0.33 0.743 -.0057802 .0080993

opennessdifflat | .0000199 .0000883 0.22 0.822 -.0001532 .0001929

nradiff | .0000833 .0033884 0.02 0.980 -.0065577 .0067244

nradifflat | -4.35e-06 .0000698 -0.06 0.950 -.0001412 .0001325

tbidiff | .0006208 .001684 0.37 0.712 -.0026799 .0039215

tbidifflat | -.0000103 .000028 -0.37 0.712 -.0000652 .0000445

----------------+----------------------------------------------------------------

Total |

gdpcdiff | -.0862463 .0491137 -1.76 0.079 -.1825074 .0100147

gdpcdifflat | .0015001 .0012113 1.24 0.216 -.0008741 .0038742

opennessdiff | .0053635 .0165578 0.32 0.746 -.0270891 .0378162

opennessdifflat | .000122 .0004288 0.28 0.776 -.0007185 .0009624

nradiff | .0001647 .0163657 0.01 0.992 -.0319115 .0322408

nradifflat | -.0000149 .0003396 -0.04 0.965 -.0006805 .0006507

tbidiff | .0028531 .0081344 0.35 0.726 -.0130901 .0187963

tbidifflat | -.0000476 .0001352 -0.35 0.725 -.0003126 .0002174

---------------------------------------------------------------------------------

Production E, Total Crops, 1992 – 2010

SAR with spatial fixed-effects Number of obs = 1008

Group variable: id Number of groups = 56

Time variable: year Panel length = 18

R-sq: within = 0.0166

between = 0.0150

overall = 0.0092

Mean of fixed-effects = -0.0002

Log-likelihood = 2897.6956

---------------------------------------------------------------------------------

ydiffshp | Coef. Std. Err. z P>|z| [95% Conf. Interval]

----------------+----------------------------------------------------------------

Main |

gdpcdiff | -.074937 .0265984 -2.82 0.005 -.1270688 -.0228051

gdpcdifflat | .0016237 .000624 2.60 0.009 .0004007 .0028467

opennessdiff | -.0096563 .0075023 -1.29 0.198 -.0243605 .0050479

opennessdifflat | .0004766 .0002006 2.38 0.018 .0000834 .0008698

nradiff | .0025779 .0086592 0.30 0.766 -.0143938 .0195497

nradifflat | -.0000421 .0001756 -0.24 0.810 -.0003863 .000302

tbidiff | .0006854 .0038355 0.18 0.858 -.006832 .0082028

tbidifflat | -.0000112 .0000638 -0.18 0.861 -.0001361 .0001138

----------------+----------------------------------------------------------------

Spatial |

rho | -.0385192 .1009559 -0.38 0.703 -.2363891 .1593508

----------------+----------------------------------------------------------------

Variance |

sigma2_e | .0001865 8.31e-06 22.45 0.000 .0001702 .0002027

----------------+----------------------------------------------------------------

Direct |

gdpcdiff | -.0753924 .0225766 -3.34 0.001 -.1196418 -.031143

gdpcdifflat | .0016554 .0005926 2.79 0.005 .0004939 .0028168

opennessdiff | -.0092328 .0080845 -1.14 0.253 -.0250781 .0066125

opennessdifflat | .0004673 .0002123 2.20 0.028 .0000512 .0008835

nradiff | .0044279 .008024 0.55 0.581 -.0112989 .0201547

nradifflat | -.0000634 .0001665 -0.38 0.703 -.0003897 .0002629

tbidiff | .0007149 .0040516 0.18 0.860 -.007226 .0086559

tbidifflat | -.0000117 .0000673 -0.17 0.862 -.0001437 .0001203

----------------+----------------------------------------------------------------

Indirect |

gdpcdiff | .0030215 .0079833 0.38 0.705 -.0126255 .0186685

gdpcdifflat | -.0000723 .0001759 -0.41 0.681 -.0004171 .0002724

opennessdiff | .0004557 .0011971 0.38 0.703 -.0018906 .0028021

opennessdifflat | -.0000216 .000048 -0.45 0.652 -.0001156 .0000724

nradiff | -.0001808 .0007371 -0.25 0.806 -.0016254 .0012638

nradifflat | 1.51e-06 .0000141 0.11 0.914 -.0000261 .0000291

tbidiff | .000022 .0004207 0.05 0.958 -.0008025 .0008465

tbidifflat | -3.71e-07 6.99e-06 -0.05 0.958 -.0000141 .0000133

----------------+----------------------------------------------------------------

Total |

gdpcdiff | -.0723709 .023394 -3.09 0.002 -.1182223 -.0265195

gdpcdifflat | .0015831 .0005729 2.76 0.006 .0004601 .002706

opennessdiff | -.0087771 .007743 -1.13 0.257 -.0239531 .0063989

opennessdifflat | .0004457 .0002012 2.22 0.027 .0000514 .00084

nradiff | .0042471 .0077534 0.55 0.584 -.0109493 .0194436

nradifflat | -.0000619 .0001611 -0.38 0.701 -.0003777 .0002539

tbidiff | .000737 .0038704 0.19 0.849 -.0068489 .0083228

tbidifflat | -.0000121 .0000643 -0.19 0.851 -.0001381 .000114

---------------------------------------------------------------------------------

Consumption PSV, Total Crops, 1992 – 2010

SAR with spatial fixed-effects Number of obs = 1008

Group variable: id Number of groups = 56

Time variable: year Panel length = 18

R-sq: within = 0.0146

between = 0.1500

overall = 0.0106

Mean of fixed-effects = 0.0009

Log-likelihood = 3262.2017

---------------------------------------------------------------------------------

ydiffpsvc | Coef. Std. Err. z P>|z| [95% Conf. Interval]

----------------+----------------------------------------------------------------

Main |

gdpcdiff | -.0040528 .018458 -0.22 0.826 -.0402298 .0321242

gdpcdifflat | -.0003924 .0004326 -0.91 0.364 -.0012402 .0004555

opennessdiff | -.0009954 .0052291 -0.19 0.849 -.0112442 .0092535

opennessdifflat | 3.08e-06 .0001396 0.02 0.982 -.0002706 .0002767

nradiff | -.0055008 .0060285 -0.91 0.362 -.0173165 .0063148

nradifflat | .0001687 .0001223 1.38 0.168 -.0000711 .0004085

tbidiff | .0025164 .0026715 0.94 0.346 -.0027197 .0077524

tbidifflat | -.0000421 .0000444 -0.95 0.343 -.0001291 .0000449

----------------+----------------------------------------------------------------

Spatial |

rho | .0467846 .1054023 0.44 0.657 -.1598002 .2533694

----------------+----------------------------------------------------------------

Variance |

sigma2_e | .0000905 4.03e-06 22.45 0.000 .0000826 .0000984

----------------+----------------------------------------------------------------

Direct |

gdpcdiff | -.0043474 .0156638 -0.28 0.781 -.0350479 .0263531

gdpcdifflat | -.0003709 .0004113 -0.90 0.367 -.001177 .0004351

opennessdiff | -.0006952 .0056363 -0.12 0.902 -.0117422 .0103518

opennessdifflat | -3.56e-06 .0001477 -0.02 0.981 -.0002931 .000286

nradiff | -.0042174 .005586 -0.75 0.450 -.0151658 .0067311

nradifflat | .000154 .000116 1.33 0.184 -.0000733 .0003814

tbidiff | .0025388 .0028221 0.90 0.368 -.0029924 .00807

tbidifflat | -.0000425 .0000469 -0.91 0.365 -.0001344 .0000495

----------------+----------------------------------------------------------------

Indirect |

gdpcdiff | -.0003662 .0026054 -0.14 0.888 -.0054727 .0047402

gdpcdifflat | -.0000156 .0000558 -0.28 0.779 -.000125 .0000937

opennessdiff | .0000836 .000645 0.13 0.897 -.0011805 .0013477

opennessdifflat | -2.36e-06 .0000162 -0.15 0.884 -.0000342 .0000294

nradiff | -.0002001 .0007613 -0.26 0.793 -.0016922 .0012921

nradifflat | 7.62e-06 .0000216 0.35 0.724 -.0000347 .00005

tbidiff | .0001714 .0004557 0.38 0.707 -.0007218 .0010645

tbidifflat | -2.86e-06 7.60e-06 -0.38 0.707 -.0000177 .000012

----------------+----------------------------------------------------------------

Total |

gdpcdiff | -.0047137 .0170391 -0.28 0.782 -.0381097 .0286823

gdpcdifflat | -.0003866 .0004323 -0.89 0.371 -.001234 .0004608

opennessdiff | -.0006116 .0059587 -0.10 0.918 -.0122904 .0110673

opennessdifflat | -5.93e-06 .0001553 -0.04 0.970 -.0003104 .0002985

nradiff | -.0044174 .0059319 -0.74 0.456 -.0160438 .0072089

nradifflat | .0001616 .0001236 1.31 0.191 -.0000807 .000404

tbidiff | .0027101 .0029792 0.91 0.363 -.003129 .0085492

tbidifflat | -.0000453 .0000495 -0.92 0.360 -.0001424 .0000517

---------------------------------------------------------------------------------

Consumption SR, Total Crops, 1992 – 2010

SAR with spatial fixed-effects Number of obs = 1008

Group variable: id Number of groups = 56

Time variable: year Panel length = 18

R-sq: within = 0.0137

between = 0.0031

overall = 0.0097

Mean of fixed-effects = 0.0074

Log-likelihood = 1614.2950

---------------------------------------------------------------------------------

ydiffsrc | Coef. Std. Err. z P>|z| [95% Conf. Interval]

----------------+----------------------------------------------------------------

Main |

gdpcdiff | .1174148 .0947055 1.24 0.215 -.0682046 .3030342

gdpcdifflat | -.0048778 .0022234 -2.19 0.028 -.0092356 -.00052

opennessdiff | .0156487 .0267958 0.58 0.559 -.0368701 .0681676

opennessdifflat | -.0007689 .0007164 -1.07 0.283 -.002173 .0006352

nradiff | -.0383863 .0309323 -1.24 0.215 -.0990124 .0222399

nradifflat | .0008396 .0006274 1.34 0.181 -.00039 .0020693

tbidiff | .0229094 .0137057 1.67 0.095 -.0039533 .0497722

tbidifflat | -.0003808 .0002278 -1.67 0.095 -.0008273 .0000657

----------------+----------------------------------------------------------------

Spatial |

rho | .0974389 .0978034 1.00 0.319 -.0942522 .28913

----------------+----------------------------------------------------------------

Variance |

sigma2_e | .0023786 .000106 22.45 0.000 .0021709 .0025863

----------------+----------------------------------------------------------------

Direct |

gdpcdiff | .1159954 .0803692 1.44 0.149 -.0415254 .2735162

gdpcdifflat | -.0047707 .0021123 -2.26 0.024 -.0089107 -.0006306

opennessdiff | .0171891 .0288805 0.60 0.552 -.0394156 .0737938

opennessdifflat | -.0008038 .0007584 -1.06 0.289 -.0022902 .0006826

nradiff | -.0318224 .0286511 -1.11 0.267 -.0879776 .0243328

nradifflat | .0007642 .0005948 1.28 0.199 -.0004015 .0019299

tbidiff | .0230354 .0144831 1.59 0.112 -.0053509 .0514218

tbidifflat | -.0003829 .0002407 -1.59 0.112 -.0008547 .0000888

----------------+----------------------------------------------------------------

Indirect |

gdpcdiff | .0113977 .017262 0.66 0.509 -.0224352 .0452306

gdpcdifflat | -.0004947 .000566 -0.87 0.382 -.001604 .0006146

opennessdiff | .0022707 .0047766 0.48 0.635 -.0070913 .0116326

opennessdifflat | -.0000997 .0001493 -0.67 0.504 -.0003922 .0001929

nradiff | -.0034079 .0056629 -0.60 0.547 -.0145069 .0076912

nradifflat | .0000796 .0001223 0.65 0.515 -.0001601 .0003194

tbidiff | .0027706 .003828 0.72 0.469 -.0047322 .0102733

tbidifflat | -.000046 .0000636 -0.72 0.469 -.0001707 .0000787

----------------+----------------------------------------------------------------

Total |

gdpcdiff | .1273931 .0903881 1.41 0.159 -.0497643 .3045505

gdpcdifflat | -.0052654 .0023465 -2.24 0.025 -.0098643 -.0006664

opennessdiff | .0194598 .0324559 0.60 0.549 -.0441527 .0830722

opennessdifflat | -.0009035 .0008576 -1.05 0.292 -.0025844 .0007774

nradiff | -.0352303 .0321445 -1.10 0.273 -.0982323 .0277718

nradifflat | .0008439 .0006651 1.27 0.205 -.0004597 .0021474

tbidiff | .025806 .0165835 1.56 0.120 -.006697 .058309

tbidifflat | -.000429 .0002756 -1.56 0.120 -.0009691 .0001112

---------------------------------------------------------------------------------

Consumption E, Total Crops, 1992 – 2010

SAR with spatial fixed-effects Number of obs = 1008

Group variable: id Number of groups = 56

Time variable: year Panel length = 18

R-sq: within = 0.0105

between = 0.0634

overall = 0.0113

Mean of fixed-effects = 0.0000

Log-likelihood = 1998.4971

---------------------------------------------------------------------------------

ydiffshc | Coef. Std. Err. z P>|z| [95% Conf. Interval]

----------------+----------------------------------------------------------------

Main |

gdpcdiff | -.0785215 .064744 -1.21 0.225 -.2054174 .0483743

gdpcdifflat | .0020983 .0015173 1.38 0.167 -.0008757 .0050722

opennessdiff | .0027418 .0183012 0.15 0.881 -.0331279 .0386115

opennessdifflat | .0001045 .000489 0.21 0.831 -.0008539 .001063

nradiff | .0367675 .0211234 1.74 0.082 -.0046336 .0781686

nradifflat | -.0007633 .0004284 -1.78 0.075 -.001603 .0000763

tbidiff | -.0211547 .0093574 -2.26 0.024 -.0394949 -.0028145

tbidifflat | .0003516 .0001555 2.26 0.024 .0000467 .0006564

----------------+----------------------------------------------------------------

Spatial |

rho | .1179564 .098034 1.20 0.229 -.0741867 .3100996

----------------+----------------------------------------------------------------

Variance |

sigma2_e | .0011096 .0000494 22.44 0.000 .0010127 .0012065

----------------+----------------------------------------------------------------

Direct |

gdpcdiff | -.079623 .0549821 -1.45 0.148 -.1873859 .0281399

gdpcdifflat | .0021755 .0014415 1.51 0.131 -.0006498 .0050008

opennessdiff | .0037908 .0197304 0.19 0.848 -.03488 .0424617

opennessdifflat | .0000813 .0005178 0.16 0.875 -.0009335 .0010961

nradiff | .0413104 .0195785 2.11 0.035 .0029371 .0796836

nradifflat | -.000816 .0004064 -2.01 0.045 -.0016125 -.0000195

tbidiff | -.0211019 .0098853 -2.13 0.033 -.0404767 -.0017271

tbidifflat | .0003507 .0001643 2.13 0.033 .0000287 .0006727

----------------+----------------------------------------------------------------

Indirect |

gdpcdiff | -.0109796 .016846 -0.65 0.515 -.0439971 .0220379

gdpcdifflat | .0002827 .0003885 0.73 0.467 -.0004788 .0010441

opennessdiff | .0008464 .0035024 0.24 0.809 -.0060182 .007711

opennessdifflat | 4.12e-06 .0000866 0.05 0.962 -.0001657 .0001739

nradiff | .0057151 .0059138 0.97 0.334 -.0058757 .0173059

nradifflat | -.0001137 .0001183 -0.96 0.337 -.0003455 .0001181

tbidiff | -.0027209 .002662 -1.02 0.307 -.0079383 .0024966

tbidifflat | .0000452 .0000442 1.02 0.307 -.0000415 .0001319

----------------+----------------------------------------------------------------

Total |

gdpcdiff | -.0906026 .0656193 -1.38 0.167 -.219214 .0380089

gdpcdifflat | .0024582 .0016476 1.49 0.136 -.0007711 .0056875

opennessdiff | .0046372 .0226286 0.20 0.838 -.0397139 .0489884

opennessdifflat | .0000854 .0005861 0.15 0.884 -.0010633 .0012341

nradiff | .0470254 .0231593 2.03 0.042 .001634 .0924169

nradifflat | -.0009297 .0004802 -1.94 0.053 -.0018709 .0000115

tbidiff | -.0238228 .0110419 -2.16 0.031 -.0454645 -.0021811

tbidifflat | .0003959 .0001835 2.16 0.031 .0000362 .0007556

---------------------------------------------------------------------------------

**SAR estimates of model (3) with contemporaneous and once lagged changes in independent variables (summarized in S3 Table)**

Production PSV, Total Crops, 1992 – 2010

SAR with spatial fixed-effects Number of obs = 952

Group variable: id Number of groups = 56

Time variable: year Panel length = 17

R-sq: within = 0.0236

between = 0.0007

overall = 0.0222

Mean of fixed-effects = 0.0004

Log-likelihood = 3992.1113

----------------------------------------------------------------------------------

ydiffpsvp | Coef. Std. Err. z P>|z| [95% Conf. Interval]

-----------------+----------------------------------------------------------------

Main |

gdpcfdiff | .005487 .0078006 0.70 0.482 -.0098019 .0207759

gdpcfdifflat | -.0001105 .0001935 -0.57 0.568 -.0004897 .0002686

gdpcsdiff | .0015444 .0072931 0.21 0.832 -.0127498 .0158386

gdpcsdifflat | -.0001191 .0001786 -0.67 0.505 -.000469 .0002309

opennessfdiff | -.0011824 .0021757 -0.54 0.587 -.0054468 .0030819

opennessfdifflat | .0000387 .0000699 0.55 0.580 -.0000983 .0001758

opennesssdiff | .0014511 .0020695 0.70 0.483 -.002605 .0055073

opennesssdifflat | -.000028 .0000553 -0.51 0.612 -.0001364 .0000803

nrafdiff | -.0003956 .0025453 -0.16 0.876 -.0053843 .0045931

nrafdifflat | .0000204 .0000516 0.40 0.692 -.0000806 .0001215

nrasdiff | .0006944 .00247 0.28 0.779 -.0041466 .0055354

nrasdifflat | 5.84e-06 .0000495 0.12 0.906 -.0000911 .0001028

tbifdiff | -.0002639 .0011142 -0.24 0.813 -.0024476 .0019198

tbifdifflat | 4.54e-06 .0000185 0.25 0.806 -.0000318 .0000408

tbisdiff | .0004362 .0011132 0.39 0.695 -.0017457 .0026181

tbisdifflat | -7.20e-06 .0000185 -0.39 0.697 -.0000435 .0000291

-----------------+----------------------------------------------------------------

Spatial |

rho | -.4334744 .1322896 -3.28 0.001 -.6927572 -.1741916

-----------------+----------------------------------------------------------------

Variance |

sigma2_e | .0000133 6.10e-07 21.74 0.000 .0000121 .0000144

-----------------+----------------------------------------------------------------

Direct |

gdpcfdiff | .0054008 .0066587 0.81 0.417 -.0076501 .0184517

gdpcfdifflat | -.0001012 .0001872 -0.54 0.589 -.0004681 .0002657

gdpcsdiff | .0019307 .0079071 0.24 0.807 -.0135669 .0174284

gdpcsdifflat | -.0001238 .0001916 -0.65 0.518 -.0004993 .0002517

opennessfdiff | -.0007255 .002125 -0.34 0.733 -.0048905 .0034395

opennessfdifflat | .0000341 .000072 0.47 0.636 -.0001071 .0001753

opennesssdiff | .0014253 .0022308 0.64 0.523 -.002947 .0057976

opennesssdifflat | -.0000295 .0000559 -0.53 0.598 -.0001391 .0000802

nrafdiff | -.0005219 .0024014 -0.22 0.828 -.0052286 .0041848

nrafdifflat | .0000232 .0000473 0.49 0.624 -.0000694 .0001159

nrasdiff | .0008518 .0023233 0.37 0.714 -.0037018 .0054053

nrasdifflat | 1.29e-06 .0000475 0.03 0.978 -.0000918 .0000944

tbifdiff | -.0003134 .0012034 -0.26 0.795 -.002672 .0020451

tbifdifflat | 5.35e-06 .00002 0.27 0.789 -.0000339 .0000446

tbisdiff | .0006303 .0010744 0.59 0.557 -.0014755 .0027361

tbisdifflat | -.0000104 .0000179 -0.58 0.560 -.0000454 .0000246

-----------------+----------------------------------------------------------------

Indirect |

gdpcfdiff | -.0016012 .0020542 -0.78 0.436 -.0056274 .002425

gdpcfdifflat | .0000309 .0000564 0.55 0.584 -.0000796 .0001414

gdpcsdiff | -.0006484 .0024983 -0.26 0.795 -.0055451 .0042482

gdpcsdifflat | .000039 .0000609 0.64 0.522 -.0000805 .0001584

opennessfdiff | .0002248 .0006934 0.32 0.746 -.0011344 .0015839

opennessfdifflat | -.0000105 .0000233 -0.45 0.652 -.0000561 .0000351

opennesssdiff | -.0004328 .0006953 -0.62 0.534 -.0017955 .0009299

opennesssdifflat | 8.75e-06 .0000177 0.49 0.622 -.000026 .0000435

nrafdiff | .000144 .000707 0.20 0.839 -.0012418 .0015297

nrafdifflat | -6.89e-06 .0000141 -0.49 0.626 -.0000346 .0000208

nrasdiff | -.0002369 .0007114 -0.33 0.739 -.0016312 .0011574

nrasdifflat | -1.00e-06 .0000142 -0.07 0.944 -.0000289 .0000269

tbifdiff | .0000809 .0003648 0.22 0.825 -.0006341 .0007959

tbifdifflat | -1.39e-06 6.06e-06 -0.23 0.819 -.0000133 .0000105

tbisdiff | -.0001853 .0003382 -0.55 0.584 -.0008482 .0004776

tbisdifflat | 3.06e-06 5.62e-06 0.54 0.586 -7.96e-06 .0000141

-----------------+----------------------------------------------------------------

Total |

gdpcfdiff | .0037996 .0047428 0.80 0.423 -.0054962 .0130954

gdpcfdifflat | -.0000703 .000134 -0.52 0.600 -.0003329 .0001924

gdpcsdiff | .0012823 .0055118 0.23 0.816 -.0095206 .0120852

gdpcsdifflat | -.0000848 .0001338 -0.63 0.526 -.0003471 .0001775

opennessfdiff | -.0005007 .0014619 -0.34 0.732 -.0033659 .0023645

opennessfdifflat | .0000236 .0000498 0.47 0.636 -.0000741 .0001213

opennesssdiff | .0009925 .0015686 0.63 0.527 -.0020819 .0040669

opennesssdifflat | -.0000207 .0000389 -0.53 0.594 -.000097 .0000555

nrafdiff | -.0003779 .0017147 -0.22 0.826 -.0037386 .0029828

nrafdifflat | .0000163 .0000336 0.49 0.627 -.0000495 .0000822

nrasdiff | .0006149 .0016577 0.37 0.711 -.0026341 .0038638

nrasdifflat | 2.89e-07 .000034 0.01 0.993 -.0000664 .000067

tbifdiff | -.0002325 .0008514 -0.27 0.785 -.0019013 .0014362

tbifdifflat | 3.97e-06 .0000142 0.28 0.779 -.0000238 .0000317

tbisdiff | .000445 .0007485 0.59 0.552 -.0010221 .0019121

tbisdifflat | -7.36e-06 .0000124 -0.59 0.554 -.0000318 .000017

----------------------------------------------------------------------------------

Production SR, Total Crops, 1992 – 2010

SAR with spatial fixed-effects Number of obs = 952

Group variable: id Number of groups = 56

Time variable: year Panel length = 17

R-sq: within = 0.0140

between = 0.1045

overall = 0.0207

Mean of fixed-effects = 0.0042

Log-likelihood = 2266.8675

----------------------------------------------------------------------------------

ydiffsrp | Coef. Std. Err. z P>|z| [95% Conf. Interval]

-----------------+----------------------------------------------------------------

Main |

gdpcfdiff | -.0639574 .0478943 -1.34 0.182 -.1578285 .0299136

gdpcfdifflat | .0006193 .0011876 0.52 0.602 -.0017083 .002947

gdpcsdiff | .0384515 .0447757 0.86 0.390 -.0493073 .1262102

gdpcsdifflat | -.0013656 .0010957 -1.25 0.213 -.0035131 .000782

opennessfdiff | 5.32e-06 .0133578 0.00 1.000 -.0261755 .0261862

opennessfdifflat | .0004421 .0004293 1.03 0.303 -.0003994 .0012836

opennesssdiff | .0183934 .0127051 1.45 0.148 -.0065081 .0432949

opennesssdifflat | -.0005062 .0003394 -1.49 0.136 -.0011714 .000159

nrafdiff | -.0040163 .015626 -0.26 0.797 -.0346427 .0266101

nrafdifflat | .0001211 .0003163 0.38 0.702 -.0004989 .0007411

nrasdiff | -.0019962 .0151626 -0.13 0.895 -.0317145 .027722

nrasdifflat | -3.24e-06 .0003038 -0.01 0.991 -.0005986 .0005921

tbifdiff | .0004473 .0068412 0.07 0.948 -.0129611 .0138557

tbifdifflat | -7.95e-06 .0001137 -0.07 0.944 -.0002308 .0002149

tbisdiff | .0012443 .0068359 0.18 0.856 -.0121538 .0146424

tbisdifflat | -.0000199 .0001136 -0.18 0.861 -.0002426 .0002028

-----------------+----------------------------------------------------------------

Spatial |

rho | .2040413 .0910094 2.24 0.025 .0256661 .3824164

-----------------+----------------------------------------------------------------

Variance |

sigma2_e | .0004994 .0000229 21.80 0.000 .0004545 .0005443

-----------------+----------------------------------------------------------------

Direct |

gdpcfdiff | -.0648585 .0407126 -1.59 0.111 -.1446538 .0149368

gdpcfdifflat | .0006828 .0011444 0.60 0.551 -.0015603 .0029258

gdpcsdiff | .0407971 .0482902 0.84 0.398 -.05385 .1354442

gdpcsdifflat | -.0013921 .0011698 -1.19 0.234 -.0036849 .0009007

opennessfdiff | .0028484 .0129674 0.22 0.826 -.0225672 .028264

opennessfdifflat | .0004133 .0004397 0.94 0.347 -.0004486 .0012752

opennesssdiff | .0182135 .0136307 1.34 0.181 -.0085022 .0449292

opennesssdifflat | -.0005152 .0003417 -1.51 0.132 -.001185 .0001545

nrafdiff | -.004788 .0146963 -0.33 0.745 -.0335923 .0240162

nrafdifflat | .0001376 .000289 0.48 0.634 -.0004287 .000704

nrasdiff | -.0010591 .0141979 -0.07 0.941 -.0288864 .0267681

nrasdifflat | -.0000315 .0002905 -0.11 0.914 -.0006009 .0005379

tbifdiff | .0001523 .0073693 0.02 0.984 -.0142912 .0145959

tbifdifflat | -3.13e-06 .0001225 -0.03 0.980 -.0002432 .000237

tbisdiff | .0024165 .0065686 0.37 0.713 -.0104577 .0152908

tbisdifflat | -.0000394 .0001092 -0.36 0.718 -.0002535 .0001746

-----------------+----------------------------------------------------------------

Indirect |

gdpcfdiff | -.0166406 .0159608 -1.04 0.297 -.0479232 .0146421

gdpcfdifflat | .0001743 .0003959 0.44 0.660 -.0006016 .0009502

gdpcsdiff | .0100329 .0156026 0.64 0.520 -.0205475 .0406134

gdpcsdifflat | -.000358 .0004145 -0.86 0.388 -.0011704 .0004544

opennessfdiff | .0007021 .0036107 0.19 0.846 -.0063747 .007779

opennessfdifflat | .000112 .0001439 0.78 0.436 -.00017 .0003941

opennesssdiff | .0047109 .0048955 0.96 0.336 -.0048841 .0143058

opennesssdifflat | -.0001387 .0001289 -1.08 0.282 -.0003912 .0001139

nrafdiff | -.001471 .0050302 -0.29 0.770 -.0113299 .0083879

nrafdifflat | .0000411 .0000956 0.43 0.667 -.0001462 .0002284

nrasdiff | .0000325 .0043928 0.01 0.994 -.0085773 .0086423

nrasdifflat | -.0000147 .0000989 -0.15 0.881 -.0002086 .0001791

tbifdiff | -.0002278 .0022206 -0.10 0.918 -.00458 .0041245

tbifdifflat | 3.63e-06 .0000369 0.10 0.921 -.0000686 .0000759

tbisdiff | .000745 .0018789 0.40 0.692 -.0029375 .0044275

tbisdifflat | -.0000122 .0000312 -0.39 0.696 -.0000733 .000049

-----------------+----------------------------------------------------------------

Total |

gdpcfdiff | -.0814991 .0529179 -1.54 0.124 -.1852163 .0222182

gdpcfdifflat | .0008571 .0014945 0.57 0.566 -.002072 .0037863

gdpcsdiff | .0508301 .0609429 0.83 0.404 -.0686159 .170276

gdpcsdifflat | -.0017501 .0014975 -1.17 0.243 -.0046852 .001185

opennessfdiff | .0035505 .016084 0.22 0.825 -.0279735 .0350745

opennessfdifflat | .0005253 .0005577 0.94 0.346 -.0005678 .0016184

opennesssdiff | .0229243 .0174758 1.31 0.190 -.0113275 .0571762

opennesssdifflat | -.0006539 .0004415 -1.48 0.139 -.0015193 .0002115

nrafdiff | -.006259 .0193466 -0.32 0.746 -.0441776 .0316596

nrafdifflat | .0001787 .0003772 0.47 0.636 -.0005605 .0009179

nrasdiff | -.0010266 .0181241 -0.06 0.955 -.0365491 .0344959

nrasdifflat | -.0000462 .0003791 -0.12 0.903 -.0007893 .0006968

tbifdiff | -.0000754 .0094239 -0.01 0.994 -.018546 .0183951

tbifdifflat | 5.04e-07 .0001566 0.00 0.997 -.0003065 .0003075

tbisdiff | .0031615 .0082828 0.38 0.703 -.0130724 .0193955

tbisdifflat | -.0000516 .0001377 -0.37 0.708 -.0003215 .0002183

----------------------------------------------------------------------------------

Production E, Total Crops, 1992 – 2010

SAR with spatial fixed-effects Number of obs = 952

Group variable: id Number of groups = 56

Time variable: year Panel length = 17

R-sq: within = 0.0334

between = 0.0432

overall = 0.0222

Mean of fixed-effects = -0.0005

Log-likelihood = 2749.6165

----------------------------------------------------------------------------------

ydiffshp | Coef. Std. Err. z P>|z| [95% Conf. Interval]

-----------------+----------------------------------------------------------------

Main |

gdpcfdiff | -.0881764 .028974 -3.04 0.002 -.1449643 -.0313885

gdpcfdifflat | .0030336 .0007231 4.20 0.000 .0016163 .0044509

gdpcsdiff | -.0074806 .0269791 -0.28 0.782 -.0603587 .0453975

gdpcsdifflat | .0011526 .0006602 1.75 0.081 -.0001414 .0024466

opennessfdiff | -.0041295 .008045 -0.51 0.608 -.0198974 .0116385

opennessfdifflat | .0000118 .0002588 0.05 0.964 -.0004954 .000519

opennesssdiff | -.0049176 .0076552 -0.64 0.521 -.0199215 .0100863

opennesssdifflat | .0000967 .0002044 0.47 0.636 -.0003039 .0004974

nrafdiff | -.000351 .0094171 -0.04 0.970 -.0188083 .0181062

nrafdifflat | -.0000156 .0001906 -0.08 0.935 -.0003891 .0003579

nrasdiff | .0018931 .0091365 0.21 0.836 -.0160141 .0198003

nrasdifflat | -.0002578 .000183 -1.41 0.159 -.0006164 .0001009

tbifdiff | .0009662 .0041194 0.23 0.815 -.0071077 .00904

tbifdifflat | -.0000157 .0000685 -0.23 0.818 -.0001499 .0001185

tbisdiff | .0005887 .0041178 0.14 0.886 -.0074819 .0086594

tbisdifflat | -9.80e-06 .0000684 -0.14 0.886 -.000144 .0001244

-----------------+----------------------------------------------------------------

Spatial |

rho | -.1893703 .1104838 -1.71 0.087 -.4059145 .027174

-----------------+----------------------------------------------------------------

Variance |

sigma2_e | .0001812 8.31e-06 21.80 0.000 .0001649 .0001975

-----------------+----------------------------------------------------------------

Direct |

gdpcfdiff | -.0888128 .0246389 -3.60 0.000 -.137104 -.0405215

gdpcfdifflat | .0030774 .0006968 4.42 0.000 .0017117 .0044432

gdpcsdiff | -.006109 .0290986 -0.21 0.834 -.0631413 .0509232

gdpcsdifflat | .0011406 .0007044 1.62 0.105 -.00024 .0025212

opennessfdiff | -.0024281 .0078113 -0.31 0.756 -.0177379 .0128817

opennessfdifflat | -5.77e-06 .0002648 -0.02 0.983 -.0005249 .0005133

opennesssdiff | -.0050553 .008206 -0.62 0.538 -.0211386 .0110281

opennesssdifflat | .0000923 .0002058 0.45 0.654 -.000311 .0004957

nrafdiff | -.0007977 .0088409 -0.09 0.928 -.0181256 .0165301

nrafdifflat | -5.96e-06 .0001738 -0.03 0.973 -.0003467 .0003348

nrasdiff | .0024543 .0085489 0.29 0.774 -.0143012 .0192098

nrasdifflat | -.0002754 .000175 -1.57 0.116 -.0006184 .0000676

tbifdiff | .0007937 .0044333 0.18 0.858 -.0078953 .0094827

tbifdifflat | -.0000129 .0000737 -0.18 0.861 -.0001574 .0001315

tbisdiff | .0012956 .0039536 0.33 0.743 -.0064533 .0090444

tbisdifflat | -.0000215 .0000657 -0.33 0.743 -.0001504 .0001073

-----------------+----------------------------------------------------------------

Indirect |

gdpcfdiff | .0146985 .0091266 1.61 0.107 -.0031894 .0325863

gdpcfdifflat | -.0005085 .0003018 -1.68 0.092 -.0011 .0000831

gdpcsdiff | .0006011 .005264 0.11 0.909 -.0097161 .0109183

gdpcsdifflat | -.0001823 .0001502 -1.21 0.225 -.0004767 .0001122

opennessfdiff | .0004027 .0015784 0.26 0.799 -.0026909 .0034963

opennessfdifflat | 2.65e-07 .0000511 0.01 0.996 -.0000999 .0001005

opennesssdiff | .0007754 .0014884 0.52 0.602 -.0021419 .0036927

opennesssdifflat | -.0000161 .0000393 -0.41 0.682 -.0000931 .0000609

nrafdiff | .0000369 .0014204 0.03 0.979 -.0027471 .0028208

nrafdifflat | 2.29e-06 .0000285 0.08 0.936 -.0000535 .0000581

nrasdiff | -.0002924 .0015489 -0.19 0.850 -.0033282 .0027434

nrasdifflat | .0000428 .0000376 1.14 0.255 -.0000309 .0001165

tbifdiff | -.0002115 .0007815 -0.27 0.787 -.0017432 .0013203

tbifdifflat | 3.47e-06 .000013 0.27 0.789 -.000022 .0000289

tbisdiff | -.0001845 .0007292 -0.25 0.800 -.0016138 .0012447

tbisdifflat | 3.07e-06 .0000121 0.25 0.800 -.0000207 .0000268

-----------------+----------------------------------------------------------------

Total |

gdpcfdiff | -.0741143 .0209854 -3.53 0.000 -.1152449 -.0329837

gdpcfdifflat | .002569 .0006009 4.28 0.000 .0013913 .0037467

gdpcsdiff | -.0055079 .0244929 -0.22 0.822 -.0535131 .0424972

gdpcsdifflat | .0009584 .0006046 1.59 0.113 -.0002266 .0021434

opennessfdiff | -.0020254 .0064506 -0.31 0.754 -.0146684 .0106175

opennessfdifflat | -5.50e-06 .0002198 -0.03 0.980 -.0004362 .0004252

opennesssdiff | -.0042799 .0069922 -0.61 0.540 -.0179843 .0094246

opennesssdifflat | .0000763 .0001724 0.44 0.658 -.0002617 .0004142

nrafdiff | -.0007609 .0076369 -0.10 0.921 -.0157289 .0142071

nrafdifflat | -3.67e-06 .0001486 -0.02 0.980 -.0002949 .0002876

nrasdiff | .0021619 .0072603 0.30 0.766 -.0120681 .0163919

nrasdifflat | -.0002326 .0001546 -1.50 0.132 -.0005356 .0000704

tbifdiff | .0005822 .0037269 0.16 0.876 -.0067223 .0078868

tbifdifflat | -9.45e-06 .000062 -0.15 0.879 -.0001309 .000112

tbisdiff | .001111 .0032883 0.34 0.735 -.0053339 .007556

tbisdifflat | -.0000185 .0000547 -0.34 0.735 -.0001256 .0000887

----------------------------------------------------------------------------------

Consumption PSV, Total Crops, 1992 – 2010

SAR with spatial fixed-effects Number of obs = 952

Group variable: id Number of groups = 56

Time variable: year Panel length = 17

R-sq: within = 0.0405

between = 0.0773

overall = 0.0360

Mean of fixed-effects = 0.0005

Log-likelihood = 3105.0500

----------------------------------------------------------------------------------

ydiffpsvc | Coef. Std. Err. z P>|z| [95% Conf. Interval]

-----------------+----------------------------------------------------------------

Main |

gdpcfdiff | -.0004449 .0198708 -0.02 0.982 -.0393909 .0385011

gdpcfdifflat | -.0004904 .0004927 -1.00 0.320 -.001456 .0004753

gdpcsdiff | -.0201406 .0185959 -1.08 0.279 -.0565879 .0163067

gdpcsdifflat | .0003247 .0004548 0.71 0.475 -.0005666 .0012161

opennessfdiff | -.0003163 .0055416 -0.06 0.954 -.0111777 .0105451

opennessfdifflat | -5.42e-06 .0001781 -0.03 0.976 -.0003546 .0003437

opennesssdiff | -.0042347 .0052711 -0.80 0.422 -.0145658 .0060965

opennesssdifflat | -.0001184 .0001408 -0.84 0.400 -.0003944 .0001576

nrafdiff | .0013699 .0064832 0.21 0.833 -.0113369 .0140766

nrafdifflat | .000035 .0001313 0.27 0.790 -.0002224 .0002924

nrasdiff | -.0165479 .0062939 -2.63 0.009 -.0288837 -.004212

nrasdifflat | .0003109 .0001262 2.46 0.014 .0000636 .0005581

tbifdiff | .0028553 .0028371 1.01 0.314 -.0027053 .008416

tbifdifflat | -.0000475 .0000472 -1.01 0.314 -.00014 .0000449

tbisdiff | .0030296 .0028355 1.07 0.285 -.002528 .0085871

tbisdifflat | -.0000507 .0000471 -1.08 0.282 -.0001431 .0000416

-----------------+----------------------------------------------------------------

Spatial |

rho | .1086686 .1058406 1.03 0.305 -.0987752 .3161125

-----------------+----------------------------------------------------------------

Variance |

sigma2_e | .000086 3.94e-06 21.81 0.000 .0000782 .0000937

-----------------+----------------------------------------------------------------

Direct |

gdpcfdiff | -.0007572 .0168693 -0.04 0.964 -.0338204 .032306

gdpcfdifflat | -.0004651 .0004743 -0.98 0.327 -.0013947 .0004644

gdpcsdiff | -.0192198 .0200345 -0.96 0.337 -.0584868 .0200472

gdpcsdifflat | .0003153 .0004849 0.65 0.516 -.0006351 .0012657

opennessfdiff | .0008624 .0053738 0.16 0.873 -.0096701 .0113948

opennessfdifflat | -.0000176 .0001822 -0.10 0.923 -.0003747 .0003396

opennesssdiff | -.0043289 .0056464 -0.77 0.443 -.0153956 .0067378

opennesssdifflat | -.0001217 .0001416 -0.86 0.390 -.0003992 .0001558

nrafdiff | .0010547 .0060872 0.17 0.862 -.0108759 .0129853

nrafdifflat | .0000419 .0001198 0.35 0.726 -.0001929 .0002767

nrasdiff | -.0161825 .0058813 -2.75 0.006 -.0277096 -.0046555

nrasdifflat | .0002997 .0001203 2.49 0.013 .0000639 .0005356

tbifdiff | .0027364 .0030492 0.90 0.369 -.00324 .0087128

tbifdifflat | -.0000456 .0000507 -0.90 0.368 -.0001449 .0000538

tbisdiff | .0035166 .0027203 1.29 0.196 -.0018151 .0088483

tbisdifflat | -.0000588 .0000452 -1.30 0.193 -.0001475 .0000298

-----------------+----------------------------------------------------------------

Indirect |

gdpcfdiff | .0000881 .0034153 0.03 0.979 -.0066058 .006782

gdpcfdifflat | -.0000607 .0001207 -0.50 0.615 -.0002974 .0001759

gdpcsdiff | -.0027198 .0044258 -0.61 0.539 -.0113942 .0059547

gdpcsdifflat | .0000439 .0001012 0.43 0.664 -.0001544 .0002422

opennessfdiff | .0001141 .0009857 0.12 0.908 -.0018179 .0020461

opennessfdifflat | -4.68e-07 .0000346 -0.01 0.989 -.0000683 .0000674

opennesssdiff | -.0005913 .0012155 -0.49 0.627 -.0029736 .001791

opennesssdifflat | -.0000168 .0000293 -0.57 0.567 -.0000741 .0000406

nrafdiff | .0000434 .0012252 0.04 0.972 -.0023579 .0024448

nrafdifflat | 7.91e-06 .0000243 0.33 0.745 -.0000397 .0000555

nrasdiff | -.0020141 .002312 -0.87 0.384 -.0065455 .0025174

nrasdifflat | .000037 .0000463 0.80 0.425 -.0000538 .0001277

tbifdiff | .0002815 .0005742 0.49 0.624 -.000844 .0014069

tbifdifflat | -4.69e-06 9.55e-06 -0.49 0.623 -.0000234 .000014

tbisdiff | .0004928 .0006894 0.71 0.475 -.0008585 .0018441

tbisdifflat | -8.24e-06 .0000115 -0.72 0.474 -.0000308 .0000143

-----------------+----------------------------------------------------------------

Total |

gdpcfdiff | -.0006691 .0194448 -0.03 0.973 -.0387801 .037442

gdpcfdifflat | -.0005258 .0005586 -0.94 0.347 -.0016206 .000569

gdpcsdiff | -.0219396 .0229736 -0.95 0.340 -.066967 .0230879

gdpcsdifflat | .0003592 .000554 0.65 0.517 -.0007266 .0014451

opennessfdiff | .0009765 .0059763 0.16 0.870 -.0107367 .0126898

opennessfdifflat | -.000018 .0002042 -0.09 0.930 -.0004182 .0003821

opennesssdiff | -.0049202 .0064649 -0.76 0.447 -.0175912 .0077509

opennesssdifflat | -.0001384 .00016 -0.87 0.387 -.0004521 .0001752

nrafdiff | .0010981 .0071052 0.15 0.877 -.0128277 .015024

nrafdifflat | .0000498 .0001396 0.36 0.721 -.0002239 .0003235

nrasdiff | -.0181966 .0067757 -2.69 0.007 -.0314767 -.0049165

nrasdifflat | .0003367 .0001406 2.39 0.017 .0000611 .0006122

tbifdiff | .0030179 .0034464 0.88 0.381 -.0037369 .0097728

tbifdifflat | -.0000503 .0000573 -0.88 0.380 -.0001625 .000062

tbisdiff | .0040094 .0031452 1.27 0.202 -.002155 .0101738

tbisdifflat | -.0000671 .0000523 -1.28 0.200 -.0001696 .0000354

----------------------------------------------------------------------------------

Consumption SR, Total Crops, 1992 – 2010

SAR with spatial fixed-effects Number of obs = 952

Group variable: id Number of groups = 56

Time variable: year Panel length = 17

R-sq: within = 0.0423

between = 0.0049

overall = 0.0000

Mean of fixed-effects = 0.3524

Log-likelihood = 1397.3488

----------------------------------------------------------------------------------

ydiffsrc | Coef. Std. Err. z P>|z| [95% Conf. Interval]

-----------------+----------------------------------------------------------------

Main |

gdpcfdiff | .1548409 .1189723 1.30 0.193 -.0783405 .3880223

gdpcfdifflat | -.0001053 .0029559 -0.04 0.972 -.0058987 .0056881

gdpcsdiff | -.0341554 .1113303 -0.31 0.759 -.2523587 .184048

gdpcsdifflat | -.0029273 .0027223 -1.08 0.282 -.0082629 .0024083

opennessfdiff | -.0316938 .0331771 -0.96 0.339 -.0967198 .0333322

opennessfdifflat | .0009632 .0010661 0.90 0.366 -.0011263 .0030528

opennesssdiff | .0612118 .0315669 1.94 0.052 -.0006581 .1230818

opennesssdifflat | -.0017543 .0008446 -2.08 0.038 -.0034096 -.0000989

nrafdiff | -.0384115 .0388238 -0.99 0.322 -.1145048 .0376819

nrafdifflat | .0011388 .0007865 1.45 0.148 -.0004026 .0026803

nrasdiff | .0209664 .0376723 0.56 0.578 -.05287 .0948027

nrasdifflat | -.0007446 .0007545 -0.99 0.324 -.0022233 .0007342

tbifdiff | -.0011669 .0169949 -0.07 0.945 -.0344763 .0321425

tbifdifflat | .0000185 .0002825 0.07 0.948 -.0005351 .0005722

tbisdiff | .00534 .0169882 0.31 0.753 -.0279562 .0386362

tbisdifflat | -.0000883 .0002824 -0.31 0.754 -.0006418 .0004651

-----------------+----------------------------------------------------------------

Spatial |

rho | .4180688 .0785936 5.32 0.000 .2640281 .5721095

-----------------+----------------------------------------------------------------

Variance |

sigma2_e | .0030822 .0001417 21.75 0.000 .0028044 .0033599

-----------------+----------------------------------------------------------------

Direct |

gdpcfdiff | .1545725 .102064 1.51 0.130 -.0454693 .3546143

gdpcfdifflat | .000048 .0028737 0.02 0.987 -.0055843 .0056803

gdpcsdiff | -.0289031 .1209997 -0.24 0.811 -.2660581 .2082519

gdpcsdifflat | -.0030148 .0029289 -1.03 0.303 -.0087553 .0027256

opennessfdiff | -.0249009 .0324354 -0.77 0.443 -.0884732 .0386713

opennessfdifflat | .0008984 .0010991 0.82 0.414 -.0012557 .0030526

opennesssdiff | .0612755 .0341247 1.80 0.073 -.0056077 .1281587

opennesssdifflat | -.0017923 .0008563 -2.09 0.036 -.0034706 -.000114

nrafdiff | -.0407301 .0368462 -1.11 0.269 -.1129473 .0314872

nrafdifflat | .0011916 .0007252 1.64 0.100 -.0002298 .0026129

nrasdiff | .0235345 .0355469 0.66 0.508 -.0461361 .0932052

nrasdifflat | -.0008232 .0007285 -1.13 0.258 -.0022509 .0006045

tbifdiff | -.0019216 .0184539 -0.10 0.917 -.0380905 .0342474

tbifdifflat | .0000309 .0003067 0.10 0.920 -.0005703 .0006321

tbisdiff | .0083152 .0164397 0.51 0.613 -.0239061 .0405366

tbisdifflat | -.0001378 .0002734 -0.50 0.614 -.0006735 .000398

-----------------+----------------------------------------------------------------

Indirect |

gdpcfdiff | .1132636 .0937801 1.21 0.227 -.070542 .2970693

gdpcfdifflat | -.0000402 .0023252 -0.02 0.986 -.0045975 .0045172

gdpcsdiff | -.0224678 .0905725 -0.25 0.804 -.1999866 .155051

gdpcsdifflat | -.0021391 .0023279 -0.92 0.358 -.0067017 .0024235

opennessfdiff | -.0179663 .0243044 -0.74 0.460 -.0656021 .0296694

opennessfdifflat | .0006459 .0008311 0.78 0.437 -.000983 .0022748

opennesssdiff | .0435407 .0293159 1.49 0.137 -.0139174 .1009989

opennesssdifflat | -.0012869 .0007666 -1.68 0.093 -.0027895 .0002156

nrafdiff | -.0297765 .0316167 -0.94 0.346 -.0917442 .0321912

nrafdifflat | .000861 .0006386 1.35 0.178 -.0003905 .0021126

nrasdiff | .0179073 .029074 0.62 0.538 -.0390766 .0748912

nrasdifflat | -.0006111 .0006524 -0.94 0.349 -.0018898 .0006677

tbifdiff | -.0024228 .013949 -0.17 0.862 -.0297624 .0249167

tbifdifflat | .0000395 .0002318 0.17 0.865 -.0004147 .0004938

tbisdiff | .0063645 .0121055 0.53 0.599 -.0173618 .0300907

tbisdifflat | -.0001055 .0002012 -0.52 0.600 -.0004999 .0002889

-----------------+----------------------------------------------------------------

Total |

gdpcfdiff | .2678361 .1890779 1.42 0.157 -.1027497 .638422

gdpcfdifflat | 7.83e-06 .0051361 0.00 0.999 -.0100588 .0100744

gdpcsdiff | -.0513709 .2092015 -0.25 0.806 -.4613983 .3586566

gdpcsdifflat | -.0051539 .0051296 -1.00 0.315 -.0152078 .0049

opennessfdiff | -.0428673 .0554368 -0.77 0.439 -.1515214 .0657868

opennessfdifflat | .0015444 .0018869 0.82 0.413 -.0021538 .0052425

opennesssdiff | .1048162 .0606791 1.73 0.084 -.0141126 .223745

opennesssdifflat | -.0030792 .00154 -2.00 0.046 -.0060976 -.0000608

nrafdiff | -.0705066 .0670689 -1.05 0.293 -.2019592 .0609461

nrafdifflat | .0020526 .0013202 1.55 0.120 -.0005349 .0046401

nrasdiff | .0414418 .0633101 0.65 0.513 -.0826436 .1655273

nrasdifflat | -.0014343 .0013431 -1.07 0.286 -.0040667 .0011982

tbifdiff | -.0043444 .0320993 -0.14 0.892 -.0672578 .058569

tbifdifflat | .0000704 .0005335 0.13 0.895 -.0009752 .001116

tbisdiff | .0146797 .0282067 0.52 0.603 -.0406043 .0699637

tbisdifflat | -.0002433 .000469 -0.52 0.604 -.0011624 .0006759

----------------------------------------------------------------------------------

Consumption E, Total Crops, 1992 – 2010

SAR with spatial fixed-effects Number of obs = 952

Group variable: id Number of groups = 56

Time variable: year Panel length = 17

R-sq: within = 0.0249

between = 0.0151

overall = 0.0226

Mean of fixed-effects = -0.0010

Log-likelihood = 1891.1642

----------------------------------------------------------------------------------

ydiffshc | Coef. Std. Err. z P>|z| [95% Conf. Interval]

-----------------+----------------------------------------------------------------

Main |

gdpcfdiff | -.048944 .0711084 -0.69 0.491 -.1883139 .0904259

gdpcfdifflat | .0018205 .001763 1.03 0.302 -.0016348 .0052759

gdpcsdiff | -.0791716 .0664723 -1.19 0.234 -.2094549 .0511117

gdpcsdifflat | .0011923 .001628 0.73 0.464 -.0019985 .004383

opennessfdiff | .0100081 .0198373 0.50 0.614 -.0288722 .0488884

opennessfdifflat | -.0003988 .0006371 -0.63 0.531 -.0016476 .0008499

opennesssdiff | -.0141609 .0188672 -0.75 0.453 -.05114 .0228183

opennesssdifflat | .0010012 .0005043 1.99 0.047 .0000127 .0019897

nrafdiff | .0456568 .0232128 1.97 0.049 .0001605 .091153

nrafdifflat | -.0009332 .0004697 -1.99 0.047 -.0018538 -.0000125

nrasdiff | .0106915 .0225352 0.47 0.635 -.0334767 .0548597

nrasdifflat | -.0002077 .0004512 -0.46 0.645 -.0010921 .0006766

tbifdiff | -.0176823 .010159 -1.74 0.082 -.0375935 .002229

tbifdifflat | .0002934 .0001689 1.74 0.082 -.0000375 .0006244

tbisdiff | -.0091926 .0101627 -0.90 0.366 -.029111 .0107259

tbisdifflat | .0001536 .0001689 0.91 0.363 -.0001775 .0004847

-----------------+----------------------------------------------------------------

Spatial |

rho | .1423596 .1001104 1.42 0.155 -.0538532 .3385724

-----------------+----------------------------------------------------------------

Variance |

sigma2_e | .0011007 .0000505 21.81 0.000 .0010018 .0011996

-----------------+----------------------------------------------------------------

Direct |

gdpcfdiff | -.050123 .0603874 -0.83 0.407 -.1684802 .0682342

gdpcfdifflat | .001915 .0016972 1.13 0.259 -.0014114 .0052414

gdpcsdiff | -.0759309 .0716396 -1.06 0.289 -.2163418 .0644801

gdpcsdifflat | .0011596 .0017365 0.67 0.504 -.0022439 .0045631

opennessfdiff | .0142426 .0192391 0.74 0.459 -.0234653 .0519506

opennessfdifflat | -.0004437 .0006519 -0.68 0.496 -.0017214 .0008339

opennesssdiff | -.0145166 .0202212 -0.72 0.473 -.0541494 .0251162

opennesssdifflat | .0009913 .0005071 1.95 0.051 -2.55e-06 .0019852

nrafdiff | .0445993 .0217967 2.05 0.041 .0018785 .0873201

nrafdifflat | -.0009106 .0004285 -2.13 0.034 -.0017504 -.0000707

nrasdiff | .0120934 .0210953 0.57 0.566 -.0292526 .0534394

nrasdifflat | -.0002497 .0004312 -0.58 0.563 -.0010949 .0005955

tbifdiff | -.018147 .0109244 -1.66 0.097 -.0395585 .0032645

tbifdifflat | .000301 .0001816 1.66 0.097 -.0000549 .0006569

tbisdiff | -.0074627 .0097395 -0.77 0.444 -.0265518 .0116263

tbisdifflat | .0001249 .0001619 0.77 0.441 -.0001925 .0004423

-----------------+----------------------------------------------------------------

Indirect |

gdpcfdiff | -.0079873 .0156049 -0.51 0.609 -.0385723 .0225977

gdpcfdifflat | .0003265 .0005114 0.64 0.523 -.0006758 .0013287

gdpcsdiff | -.0140926 .0189825 -0.74 0.458 -.0512977 .0231124

gdpcsdifflat | .0002199 .0004354 0.51 0.613 -.0006334 .0010732

opennessfdiff | .0025105 .0045495 0.55 0.581 -.0064064 .0114273

opennessfdifflat | -.000076 .0001558 -0.49 0.626 -.0003814 .0002295

opennesssdiff | -.00261 .0050105 -0.52 0.602 -.0124303 .0072104

opennesssdifflat | .00017 .0001774 0.96 0.338 -.0001777 .0005178

nrafdiff | .0076015 .0079585 0.96 0.340 -.0079969 .0232

nrafdifflat | -.0001547 .000158 -0.98 0.327 -.0004644 .000155

nrasdiff | .0024092 .0057686 0.42 0.676 -.0088971 .0137155

nrasdifflat | -.0000519 .0001261 -0.41 0.680 -.0002991 .0001952

tbifdiff | -.0034919 .0040728 -0.86 0.391 -.0114744 .0044905

tbifdifflat | .0000579 .0000676 0.86 0.392 -.0000746 .0001905

tbisdiff | -.0011422 .002125 -0.54 0.591 -.0053072 .0030227

tbisdifflat | .0000191 .0000354 0.54 0.589 -.0000502 .0000885

-----------------+----------------------------------------------------------------

Total |

gdpcfdiff | -.0581103 .0722559 -0.80 0.421 -.1997292 .0835087

gdpcfdifflat | .0022415 .0020761 1.08 0.280 -.0018276 .0063106

gdpcsdiff | -.0900235 .08611 -1.05 0.296 -.258796 .0787489

gdpcsdifflat | .0013795 .0020825 0.66 0.508 -.0027021 .0054611

opennessfdiff | .0167531 .022391 0.75 0.454 -.0271325 .0606387

opennessfdifflat | -.0005197 .0007623 -0.68 0.495 -.0020139 .0009744

opennesssdiff | -.0171265 .0240711 -0.71 0.477 -.064305 .0300519

opennesssdifflat | .0011614 .0006048 1.92 0.055 -.000024 .0023467

nrafdiff | .0522008 .0267939 1.95 0.051 -.0003143 .104716

nrafdifflat | -.0010653 .0005226 -2.04 0.041 -.0020895 -.0000411

nrasdiff | .0145026 .0255315 0.57 0.570 -.0355382 .0645435

nrasdifflat | -.0003017 .0005316 -0.57 0.570 -.0013436 .0007403

tbifdiff | -.0216389 .0138572 -1.56 0.118 -.0487986 .0055207

tbifdifflat | .000359 .0002303 1.56 0.119 -.0000924 .0008104

tbisdiff | -.008605 .0113022 -0.76 0.446 -.0307569 .013547

tbisdifflat | .000144 .0001879 0.77 0.443 -.0002243 .0005123

----------------------------------------------------------------------------------

**SAR estimates of model (3) with contemporaneous and once and twice lagged changes in independent variables (summarized in S4 Table)**

Production PSV, Total Crops, 1992 – 2010

SAR with spatial fixed-effects Number of obs = 896

Group variable: id Number of groups = 56

Time variable: year Panel length = 16

R-sq: within = 0.0290

between = 0.0467

overall = 0.0244

Mean of fixed-effects = 0.0004

Log-likelihood = 3759.1706

----------------------------------------------------------------------------------

ydiffpsvp | Coef. Std. Err. z P>|z| [95% Conf. Interval]

-----------------+----------------------------------------------------------------

Main |

gdpcfdiff | .0043255 .008234 0.53 0.599 -.0118128 .0204638

gdpcfdifflat | -.0000291 .0002091 -0.14 0.889 -.0004389 .0003808

gdpcsdiff | -.0017165 .0081791 -0.21 0.834 -.0177473 .0143144

gdpcsdifflat | 7.83e-06 .0002138 0.04 0.971 -.0004113 .0004269

gdpctdiff | .0029404 .0076659 0.38 0.701 -.0120845 .0179653

gdpctdifflat | -.0000789 .0002057 -0.38 0.701 -.000482 .0003242

opennessfdiff | -.0024708 .0023724 -1.04 0.298 -.0071206 .002179

opennessfdifflat | .0000394 .0000796 0.49 0.621 -.0001166 .0001953

opennesssdiff | .0016351 .0022404 0.73 0.466 -.0027561 .0060263

opennesssdifflat | -.0000585 .000072 -0.81 0.416 -.0001995 .0000826

opennesstdiff | -.0006052 .0022011 -0.27 0.783 -.0049192 .0037088

opennesstdifflat | .0000524 .0000591 0.89 0.376 -.0000635 .0001683

nrafdiff | .0006866 .0028007 0.25 0.806 -.0048027 .0061759

nrafdifflat | .0000103 .000057 0.18 0.857 -.0001015 .000122

nrasdiff | .0011702 .0027902 0.42 0.675 -.0042985 .006639

nrasdifflat | -7.63e-06 .0000553 -0.14 0.890 -.000116 .0001007

nratdiff | .0019427 .0026432 0.73 0.462 -.0032378 .0071231

nratdifflat | -.0000435 .0000533 -0.82 0.414 -.000148 .000061

tbifdiff | .0001793 .0011909 0.15 0.880 -.0021549 .0025135

tbifdifflat | -2.84e-06 .0000198 -0.14 0.886 -.0000416 .000036

tbisdiff | .0002063 .0012531 0.16 0.869 -.0022498 .0026624

tbisdifflat | -3.36e-06 .0000208 -0.16 0.872 -.0000442 .0000375

tbitdiff | .0000692 .0011727 0.06 0.953 -.0022291 .0023676

tbitdifflat | -1.12e-06 .0000195 -0.06 0.954 -.0000393 .0000371

-----------------+----------------------------------------------------------------

Spatial |

rho | -.4298189 .1353904 -3.17 0.002 -.6951792 -.1644587

-----------------+----------------------------------------------------------------

Variance |

sigma2_e | .0000132 6.26e-07 21.09 0.000 .000012 .0000144

-----------------+----------------------------------------------------------------

Direct |

gdpcfdiff | .0042226 .0070235 0.60 0.548 -.0095432 .0179883

gdpcfdifflat | -.000018 .0002035 -0.09 0.929 -.0004169 .0003808

gdpcsdiff | -.0013348 .0088436 -0.15 0.880 -.0186679 .0159982

gdpcsdifflat | 3.81e-06 .0002286 0.02 0.987 -.0004442 .0004518

gdpctdiff | .004505 .0075646 0.60 0.551 -.0103214 .0193314

gdpctdifflat | -.0000921 .0002085 -0.44 0.659 -.0005008 .0003166

opennessfdiff | -.0024649 .0025511 -0.97 0.334 -.0074649 .002535

opennessfdifflat | .0000335 .0000793 0.42 0.673 -.000122 .000189

opennesssdiff | .0014525 .0020528 0.71 0.479 -.002571 .0054759

opennesssdifflat | -.0000547 .000063 -0.87 0.386 -.0001782 .0000688

opennesstdiff | -.0005436 .0021603 -0.25 0.801 -.0047777 .0036906

opennesstdifflat | .0000499 .0000572 0.87 0.383 -.0000621 .000162

nrafdiff | .0005554 .0029922 0.19 0.853 -.0053092 .0064201

nrafdifflat | 6.46e-06 .0000604 0.11 0.915 -.0001119 .0001248

nrasdiff | .0016609 .0025667 0.65 0.518 -.0033697 .0066916

nrasdifflat | -.0000193 .0000524 -0.37 0.712 -.000122 .0000833

nratdiff | .0018769 .0029373 0.64 0.523 -.00388 .0076339

nratdifflat | -.0000398 .0000574 -0.69 0.488 -.0001522 .0000726

tbifdiff | .0002551 .0011539 0.22 0.825 -.0020065 .0025167

tbifdifflat | -4.09e-06 .0000192 -0.21 0.831 -.0000417 .0000335

tbisdiff | .000185 .0011423 0.16 0.871 -.0020538 .0024238

tbisdifflat | -3.00e-06 .000019 -0.16 0.874 -.0000402 .0000342

tbitdiff | .0001239 .0013155 0.09 0.925 -.0024545 .0027022

tbitdifflat | -2.03e-06 .0000219 -0.09 0.926 -.0000449 .0000408

-----------------+----------------------------------------------------------------

Indirect |

gdpcfdiff | -.0011899 .0020444 -0.58 0.561 -.0051969 .002817

gdpcfdifflat | 4.97e-06 .0000604 0.08 0.934 -.0001135 .0001234

gdpcsdiff | .0004924 .0026806 0.18 0.854 -.0047614 .0057463

gdpcsdifflat | -2.90e-06 .0000696 -0.04 0.967 -.0001393 .0001335

gdpctdiff | -.0014054 .0022816 -0.62 0.538 -.0058773 .0030665

gdpctdifflat | .0000284 .0000638 0.45 0.656 -.0000965 .0001534

opennessfdiff | .0007239 .0008278 0.87 0.382 -.0008985 .0023464

opennessfdifflat | -9.28e-06 .0000239 -0.39 0.697 -.0000561 .0000375

opennesssdiff | -.0004036 .0006383 -0.63 0.527 -.0016547 .0008475

opennesssdifflat | .000016 .0000197 0.81 0.419 -.0000227 .0000546

opennesstdiff | .0001461 .0006692 0.22 0.827 -.0011655 .0014577

opennesstdifflat | -.0000149 .0000178 -0.84 0.401 -.0000498 .0000199

nrafdiff | -.0001609 .0009716 -0.17 0.868 -.0020651 .0017433

nrafdifflat | -2.16e-06 .0000196 -0.11 0.912 -.0000405 .0000362

nrasdiff | -.0005007 .0007945 -0.63 0.529 -.0020579 .0010564

nrasdifflat | 6.06e-06 .0000156 0.39 0.697 -.0000245 .0000366

nratdiff | -.0005424 .0008885 -0.61 0.542 -.0022838 .001199

nratdifflat | .0000116 .0000174 0.66 0.506 -.0000226 .0000457

tbifdiff | -.0000691 .0003404 -0.20 0.839 -.0007363 .0005982

tbifdifflat | 1.10e-06 5.66e-06 0.20 0.845 -9.99e-06 .0000122

tbisdiff | -.0000554 .0003535 -0.16 0.875 -.0007482 .0006373

tbisdifflat | 8.99e-07 5.87e-06 0.15 0.878 -.0000106 .0000124

tbitdiff | -.0000448 .0004064 -0.11 0.912 -.0008413 .0007516

tbitdifflat | 7.36e-07 6.76e-06 0.11 0.913 -.0000125 .000014

-----------------+----------------------------------------------------------------

Total |

gdpcfdiff | .0030326 .005114 0.59 0.553 -.0069906 .0130559

gdpcfdifflat | -.000013 .0001454 -0.09 0.928 -.000298 .0002719

gdpcsdiff | -.0008424 .0062846 -0.13 0.893 -.0131601 .0114752

gdpcsdifflat | 9.07e-07 .0001618 0.01 0.996 -.0003163 .0003181

gdpctdiff | .0030996 .0053947 0.57 0.566 -.0074739 .0136731

gdpctdifflat | -.0000637 .0001474 -0.43 0.666 -.0003525 .0002252

opennessfdiff | -.001741 .001791 -0.97 0.331 -.0052513 .0017692

opennessfdifflat | .0000242 .0000566 0.43 0.669 -.0000867 .0001351

opennesssdiff | .0010488 .0014466 0.73 0.468 -.0017864 .003884

opennesssdifflat | -.0000387 .0000444 -0.87 0.384 -.0001258 .0000484

opennesstdiff | -.0003975 .0015187 -0.26 0.794 -.0033741 .0025791

opennesstdifflat | .000035 .0000405 0.86 0.388 -.0000444 .0001144

nrafdiff | .0003945 .0020632 0.19 0.848 -.0036494 .0044384

nrafdifflat | 4.30e-06 .0000416 0.10 0.918 -.0000772 .0000858

nrasdiff | .0011602 .0018253 0.64 0.525 -.0024172 .0047377

nrasdifflat | -.0000133 .0000378 -0.35 0.726 -.0000873 .0000608

nratdiff | .0013345 .0020963 0.64 0.524 -.0027742 .0054433

nratdifflat | -.0000282 .000041 -0.69 0.491 -.0001085 .0000521

tbifdiff | .000186 .0008281 0.22 0.822 -.001437 .001809

tbifdifflat | -2.99e-06 .0000138 -0.22 0.828 -.00003 .000024

tbisdiff | .0001296 .0008045 0.16 0.872 -.0014471 .0017063

tbisdifflat | -2.10e-06 .0000134 -0.16 0.875 -.0000283 .0000241

tbitdiff | .000079 .0009197 0.09 0.932 -.0017236 .0018817

tbitdifflat | -1.29e-06 .0000153 -0.08 0.933 -.0000313 .0000287

----------------------------------------------------------------------------------

Production SR, Total Crops, 1992 – 2010

SAR with spatial fixed-effects Number of obs = 896

Group variable: id Number of groups = 56

Time variable: year Panel length = 16

R-sq: within = 0.0187

between = 0.1456

overall = 0.0296

Mean of fixed-effects = 0.0039

Log-likelihood = 2137.5488

----------------------------------------------------------------------------------

ydiffsrp | Coef. Std. Err. z P>|z| [95% Conf. Interval]

-----------------+----------------------------------------------------------------

Main |

gdpcfdiff | -.0775112 .0504382 -1.54 0.124 -.1763682 .0213458

gdpcfdifflat | .0019009 .0012805 1.48 0.138 -.0006088 .0044107

gdpcsdiff | .0243595 .0500844 0.49 0.627 -.073804 .1225231

gdpcsdifflat | -.0004482 .0013092 -0.34 0.732 -.0030142 .0021179

gdpctdiff | .039271 .046969 0.84 0.403 -.0527866 .1313285

gdpctdifflat | -.0011902 .0012596 -0.94 0.345 -.0036589 .0012786

opennessfdiff | .0055996 .014529 0.39 0.700 -.0228768 .0340759

opennessfdifflat | -.0001734 .0004874 -0.36 0.722 -.0011287 .000782

opennesssdiff | .0205306 .0137217 1.50 0.135 -.0063634 .0474247

opennesssdifflat | -.0007754 .0004406 -1.76 0.078 -.0016389 .0000881

opennesstdiff | .0013945 .0134795 0.10 0.918 -.0250249 .0278139

opennesstdifflat | -.0000568 .0003625 -0.16 0.876 -.0007673 .0006538

nrafdiff | -.0052333 .0171476 -0.31 0.760 -.0388419 .0283753

nrafdifflat | .0001483 .0003487 0.43 0.671 -.0005351 .0008318

nrasdiff | -.0008313 .0170861 -0.05 0.961 -.0343195 .0326569

nrasdifflat | -.0000142 .0003386 -0.04 0.966 -.0006778 .0006494

nratdiff | .0064059 .0161808 0.40 0.692 -.0253079 .0381197

nratdifflat | -.0002092 .0003262 -0.64 0.521 -.0008485 .0004301

tbifdiff | .0013942 .0072936 0.19 0.848 -.0129009 .0156893

tbifdifflat | -.0000239 .0001212 -0.20 0.844 -.0002615 .0002137

tbisdiff | .0011098 .0076745 0.14 0.885 -.0139319 .0161514

tbisdifflat | -.0000174 .0001276 -0.14 0.892 -.0002674 .0002327

tbitdiff | .0028749 .0071841 0.40 0.689 -.0112057 .0169555

tbitdifflat | -.0000472 .0001194 -0.40 0.693 -.0002813 .0001868

-----------------+----------------------------------------------------------------

Spatial |

rho | .2201105 .0923968 2.38 0.017 .0390161 .401205

-----------------+----------------------------------------------------------------

Variance |

sigma2_e | .0004948 .0000234 21.15 0.000 .0004489 .0005407

-----------------+----------------------------------------------------------------

Direct |

gdpcfdiff | -.0785152 .0428895 -1.83 0.067 -.162577 .0055466

gdpcfdifflat | .0019747 .0012416 1.59 0.112 -.0004587 .0044081

gdpcsdiff | .0268791 .053964 0.50 0.618 -.0788883 .1326466

gdpcsdifflat | -.0004755 .0013944 -0.34 0.733 -.0032085 .0022576

gdpctdiff | .0487845 .046185 1.06 0.291 -.0417365 .1393055

gdpctdifflat | -.0012699 .001272 -1.00 0.318 -.0037629 .0012231

opennessfdiff | .0057433 .0155431 0.37 0.712 -.0247207 .0362073

opennessfdifflat | -.0002106 .000484 -0.44 0.663 -.0011592 .000738

opennesssdiff | .0194254 .0125249 1.55 0.121 -.0051229 .0439738

opennesssdifflat | -.0007522 .0003844 -1.96 0.050 -.0015057 1.27e-06

opennesstdiff | .0017981 .0131747 0.14 0.891 -.0240239 .02762

opennesstdifflat | -.0000745 .0003493 -0.21 0.831 -.0007591 .00061

nrafdiff | -.0060769 .0182026 -0.33 0.738 -.0417534 .0295995

nrafdifflat | .0001251 .0003667 0.34 0.733 -.0005936 .0008438

nrasdiff | .0021216 .0156424 0.14 0.892 -.0285369 .0327801

nrasdifflat | -.000085 .0003198 -0.27 0.791 -.0007118 .0005419

nratdiff | .0059635 .0179061 0.33 0.739 -.0291318 .0410589

nratdifflat | -.0001855 .0003494 -0.53 0.595 -.0008703 .0004993

tbifdiff | .0018583 .007049 0.26 0.792 -.0119576 .0156742

tbifdifflat | -.0000316 .0001172 -0.27 0.788 -.0002613 .0001981

tbisdiff | .0009723 .0069676 0.14 0.889 -.0126839 .0146284

tbisdifflat | -.0000151 .0001158 -0.13 0.897 -.000242 .0002119

tbitdiff | .0032071 .0080256 0.40 0.689 -.0125227 .0189369

tbitdifflat | -.0000527 .0001334 -0.40 0.693 -.0003142 .0002088

-----------------+----------------------------------------------------------------

Indirect |

gdpcfdiff | -.0224485 .0186829 -1.20 0.230 -.0590663 .0141693

gdpcfdifflat | .0005756 .0005281 1.09 0.276 -.0004595 .0016106

gdpcsdiff | .0095423 .020345 0.47 0.639 -.0303332 .0494178

gdpcsdifflat | -.0001783 .0005009 -0.36 0.722 -.0011601 .0008036

gdpctdiff | .0140558 .0172932 0.81 0.416 -.0198382 .0479498

gdpctdifflat | -.0003647 .0004617 -0.79 0.430 -.0012697 .0005403

opennessfdiff | .001423 .005018 0.28 0.777 -.008412 .0112581

opennessfdifflat | -.0000504 .0001655 -0.30 0.761 -.0003748 .0002741

opennesssdiff | .0061724 .0054164 1.14 0.254 -.0044435 .0167884

opennesssdifflat | -.0002306 .0001821 -1.27 0.205 -.0005875 .0001263

opennesstdiff | .0004317 .0043737 0.10 0.921 -.0081405 .0090039

opennesstdifflat | -.0000259 .0001226 -0.21 0.833 -.0002662 .0002143

nrafdiff | -.0018864 .0056215 -0.34 0.737 -.0129043 .0091315

nrafdifflat | .0000382 .0001142 0.33 0.738 -.0001855 .000262

nrasdiff | .0006524 .0054753 0.12 0.905 -.010079 .0113837

nrasdifflat | -.0000198 .0001176 -0.17 0.866 -.0002503 .0002106

nratdiff | .0021817 .0062787 0.35 0.728 -.0101244 .0144878

nratdifflat | -.0000623 .0001252 -0.50 0.619 -.0003078 .0001831

tbifdiff | .0006074 .0025309 0.24 0.810 -.004353 .0055678

tbifdifflat | -.0000103 .0000421 -0.24 0.807 -.0000928 .0000722

tbisdiff | .0003411 .0022761 0.15 0.881 -.00412 .0048022

tbisdifflat | -5.34e-06 .0000378 -0.14 0.888 -.0000794 .0000687

tbitdiff | .0008503 .0025369 0.34 0.737 -.0041219 .0058226

tbitdifflat | -.000014 .0000422 -0.33 0.740 -.0000966 .0000687

-----------------+----------------------------------------------------------------

Total |

gdpcfdiff | -.1009637 .0572655 -1.76 0.078 -.213202 .0112746

gdpcfdifflat | .0025502 .0016652 1.53 0.126 -.0007135 .0058139

gdpcsdiff | .0364215 .0722965 0.50 0.614 -.105277 .1781199

gdpcsdifflat | -.0006537 .0018497 -0.35 0.724 -.004279 .0029715

gdpctdiff | .0628403 .061149 1.03 0.304 -.0570095 .1826901

gdpctdifflat | -.0016346 .0016712 -0.98 0.328 -.0049102 .0016409

opennessfdiff | .0071663 .0199007 0.36 0.719 -.0318383 .046171

opennessfdifflat | -.000261 .0006337 -0.41 0.680 -.0015031 .0009811

opennesssdiff | .0255979 .0168849 1.52 0.130 -.007496 .0586917

opennesssdifflat | -.0009828 .0005233 -1.88 0.060 -.0020084 .0000428

opennesstdiff | .0022298 .0171216 0.13 0.896 -.0313279 .0357875

opennesstdifflat | -.0001004 .0004618 -0.22 0.828 -.0010055 .0008046

nrafdiff | -.0079634 .0231169 -0.34 0.730 -.0532716 .0373449

nrafdifflat | .0001634 .0004663 0.35 0.726 -.0007507 .0010774

nrasdiff | .0027739 .020573 0.13 0.893 -.0375483 .0430962

nrasdifflat | -.0001048 .0004262 -0.25 0.806 -.0009402 .0007306

nratdiff | .0081452 .0236762 0.34 0.731 -.0382594 .0545498

nratdifflat | -.0002478 .0004628 -0.54 0.592 -.0011549 .0006593

tbifdiff | .0024657 .0093849 0.26 0.793 -.0159285 .0208598

tbifdifflat | -.0000419 .000156 -0.27 0.788 -.0003477 .000264

tbisdiff | .0013134 .0090104 0.15 0.884 -.0163468 .0189735

tbisdifflat | -.0000204 .0001497 -0.14 0.892 -.0003139 .0002731

tbitdiff | .0040575 .0103427 0.39 0.695 -.0162139 .0243288

tbitdifflat | -.0000667 .0001719 -0.39 0.698 -.0004037 .0002703

----------------------------------------------------------------------------------

Production E, Total Crops, 1992 – 2010

SAR with spatial fixed-effects Number of obs = 896

Group variable: id Number of groups = 56

Time variable: year Panel length = 16

R-sq: within = 0.0472

between = 0.0209

overall = 0.0453

Mean of fixed-effects = 0.0005

Log-likelihood = 2601.4086

----------------------------------------------------------------------------------

ydiffshp | Coef. Std. Err. z P>|z| [95% Conf. Interval]

-----------------+----------------------------------------------------------------

Main |

gdpcfdiff | -.0917059 .0302097 -3.04 0.002 -.1509159 -.0324959

gdpcfdifflat | .0029521 .000773 3.82 0.000 .001437 .0044673

gdpcsdiff | -.012166 .0298321 -0.41 0.683 -.0706358 .0463038

gdpcsdifflat | .0012145 .0007811 1.55 0.120 -.0003165 .0027455

gdpctdiff | -.021967 .0280502 -0.78 0.434 -.0769443 .0330103

gdpctdifflat | .0014564 .0007508 1.94 0.052 -.0000153 .002928

opennessfdiff | -.0031319 .0086521 -0.36 0.717 -.0200897 .0138259

opennessfdifflat | -.000015 .0002903 -0.05 0.959 -.0005839 .000554

opennesssdiff | -.0010541 .0081772 -0.13 0.897 -.0170812 .0149729

opennesssdifflat | -.0000812 .0002625 -0.31 0.757 -.0005957 .0004333

opennesstdiff | -.0022747 .0080326 -0.28 0.777 -.0180184 .0134689

opennesstdifflat | .0002969 .0002158 1.38 0.169 -.0001261 .0007198

nrafdiff | -.0010108 .0102232 -0.10 0.921 -.0210479 .0190263

nrafdifflat | -.0000258 .0002077 -0.12 0.901 -.0004329 .0003812

nrasdiff | .0010975 .010186 0.11 0.914 -.0188666 .0210617

nrasdifflat | -.0002998 .0002017 -1.49 0.137 -.0006952 .0000955

nratdiff | -.0001306 .0096415 -0.01 0.989 -.0190276 .0187663

nratdifflat | .0001135 .0001943 0.58 0.559 -.0002673 .0004943

tbifdiff | .0014648 .0043431 0.34 0.736 -.0070474 .0099771

tbifdifflat | -.0000239 .0000722 -0.33 0.740 -.0001654 .0001176

tbisdiff | .0007431 .0045724 0.16 0.871 -.0082185 .0097048

tbisdifflat | -.0000124 .000076 -0.16 0.870 -.0001614 .0001365

tbitdiff | -.0003167 .0042773 -0.07 0.941 -.0087 .0080666

tbitdifflat | 5.07e-06 .0000711 0.07 0.943 -.0001343 .0001444

-----------------+----------------------------------------------------------------

Spatial |

rho | -.2818465 .1162814 -2.42 0.015 -.5097538 -.0539392

-----------------+----------------------------------------------------------------

Variance |

sigma2_e | .0001755 8.30e-06 21.14 0.000 .0001593 .0001918

-----------------+----------------------------------------------------------------

Direct |

gdpcfdiff | -.0925185 .0257462 -3.59 0.000 -.1429801 -.042057

gdpcfdifflat | .0030042 .0007512 4.00 0.000 .0015318 .0044766

gdpcsdiff | -.0107637 .032155 -0.33 0.738 -.0737863 .0522589

gdpcsdifflat | .0012043 .0008327 1.45 0.148 -.0004278 .0028364

gdpctdiff | -.0163881 .0276053 -0.59 0.553 -.0704934 .0377171

gdpctdifflat | .0014156 .0007587 1.87 0.062 -.0000714 .0029025

opennessfdiff | -.0030666 .0092677 -0.33 0.741 -.0212309 .0150977

opennessfdifflat | -.0000365 .0002882 -0.13 0.899 -.0006013 .0005284

opennesssdiff | -.001748 .0074712 -0.23 0.815 -.0163914 .0128954

opennesssdifflat | -.0000665 .0002294 -0.29 0.772 -.0005161 .0003831

opennesstdiff | -.0020307 .0078616 -0.26 0.796 -.0174392 .0133778

opennesstdifflat | .0002873 .0002081 1.38 0.167 -.0001205 .0006951

nrafdiff | -.0014864 .0108886 -0.14 0.891 -.0228277 .019855

nrafdifflat | -.0000401 .0002188 -0.18 0.854 -.000469 .0003887

nrasdiff | .0028499 .0093436 0.31 0.760 -.0154632 .021163

nrasdifflat | -.0003433 .0001906 -1.80 0.072 -.0007169 .0000302

nratdiff | -.0004238 .0106853 -0.04 0.968 -.0213665 .020519

nratdifflat | .0001285 .0002083 0.62 0.537 -.0002798 .0005367

tbifdiff | .001744 .0041929 0.42 0.677 -.006474 .009962

tbifdifflat | -.0000286 .0000697 -0.41 0.682 -.0001652 .0001081

tbisdiff | .0006654 .0041507 0.16 0.873 -.0074698 .0088007

tbisdifflat | -.0000111 .000069 -0.16 0.872 -.0001464 .0001241

tbitdiff | -.0001199 .0047819 -0.03 0.980 -.0094922 .0092525

tbitdifflat | 1.80e-06 .0000795 0.02 0.982 -.000154 .0001576

-----------------+----------------------------------------------------------------

Indirect |

gdpcfdiff | .0207461 .0100953 2.06 0.040 .0009597 .0405326

gdpcfdifflat | -.0006739 .0003219 -2.09 0.036 -.0013048 -.000043

gdpcsdiff | .00274 .0074847 0.37 0.714 -.0119298 .0174098

gdpcsdifflat | -.000279 .000226 -1.23 0.217 -.000722 .0001639

gdpctdiff | .0033172 .0062307 0.53 0.594 -.0088948 .0155293

gdpctdifflat | -.0003063 .0002014 -1.52 0.128 -.000701 .0000884

opennessfdiff | .0006012 .0021985 0.27 0.784 -.0037077 .0049101

opennessfdifflat | .000011 .0000633 0.17 0.862 -.0001131 .0001352

opennesssdiff | .0004711 .0018558 0.25 0.800 -.0031662 .0041083

opennesssdifflat | .0000139 .0000554 0.25 0.802 -.0000948 .0001226

opennesstdiff | .000357 .0018897 0.19 0.850 -.0033468 .0040608

opennesstdifflat | -.0000624 .0000534 -1.17 0.242 -.000167 .0000421

nrafdiff | .0002317 .0027393 0.08 0.933 -.0051372 .0056006

nrafdifflat | .0000102 .0000558 0.18 0.855 -.0000992 .0001196

nrasdiff | -.0005807 .0021596 -0.27 0.788 -.0048135 .0036521

nrasdifflat | .0000767 .000051 1.50 0.133 -.0000233 .0001767

nratdiff | .0002474 .0024529 0.10 0.920 -.0045601 .0050549

nratdifflat | -.0000315 .0000501 -0.63 0.530 -.0001296 .0000667

tbifdiff | -.0003386 .0009198 -0.37 0.713 -.0021414 .0014642

tbifdifflat | 5.53e-06 .0000153 0.36 0.717 -.0000244 .0000355

tbisdiff | -.0001287 .0010092 -0.13 0.898 -.0021068 .0018493

tbisdifflat | 2.16e-06 .0000168 0.13 0.898 -.0000307 .000035

tbitdiff | 7.41e-06 .0011506 0.01 0.995 -.0022477 .0022625

tbitdifflat | -8.17e-08 .0000191 -0.00 0.997 -.0000376 .0000374

-----------------+----------------------------------------------------------------

Total |

gdpcfdiff | -.0717724 .0201773 -3.56 0.000 -.1113191 -.0322257

gdpcfdifflat | .0023304 .000577 4.04 0.000 .0011995 .0034612

gdpcsdiff | -.0080236 .0253454 -0.32 0.752 -.0576998 .0416525

gdpcsdifflat | .0009252 .0006427 1.44 0.150 -.0003345 .002185

gdpctdiff | -.0130709 .0220265 -0.59 0.553 -.0562421 .0301003

gdpctdifflat | .0011093 .0006117 1.81 0.070 -.0000895 .0023081

opennessfdiff | -.0024654 .0072767 -0.34 0.735 -.0167275 .0117968

opennessfdifflat | -.0000254 .0002302 -0.11 0.912 -.0004766 .0004257

opennesssdiff | -.001277 .0057234 -0.22 0.823 -.0124946 .0099407

opennesssdifflat | -.0000526 .0001774 -0.30 0.767 -.0004003 .0002951

opennesstdiff | -.0016737 .0061536 -0.27 0.786 -.0137345 .0103872

opennesstdifflat | .0002248 .0001662 1.35 0.176 -.000101 .0005506

nrafdiff | -.0012547 .0083257 -0.15 0.880 -.0175726 .0150633

nrafdifflat | -.00003 .0001668 -0.18 0.857 -.0003569 .000297

nrasdiff | .0022692 .0073686 0.31 0.758 -.0121731 .0167115

nrasdifflat | -.0002667 .0001517 -1.76 0.079 -.000564 .0000307

nratdiff | -.0001764 .0083862 -0.02 0.983 -.016613 .0162603

nratdifflat | .000097 .0001619 0.60 0.549 -.0002203 .0004144

tbifdiff | .0014054 .003372 0.42 0.677 -.0052036 .0080144

tbifdifflat | -.000023 .0000561 -0.41 0.681 -.0001329 .0000868

tbisdiff | .0005367 .0032119 0.17 0.867 -.0057585 .0068319

tbisdifflat | -8.99e-06 .0000534 -0.17 0.866 -.0001136 .0000956

tbitdiff | -.0001124 .003694 -0.03 0.976 -.0073526 .0071277

tbitdifflat | 1.72e-06 .0000614 0.03 0.978 -.0001186 .0001221

----------------------------------------------------------------------------------

Consumption PSV, Total Crops, 1992 – 2010

SAR with spatial fixed-effects Number of obs = 896

Group variable: id Number of groups = 56

Time variable: year Panel length = 16

R-sq: within = 0.0569

between = 0.0254

overall = 0.0531

Mean of fixed-effects = 0.0008

Log-likelihood = 2951.9167

----------------------------------------------------------------------------------

ydiffpsvc | Coef. Std. Err. z P>|z| [95% Conf. Interval]

-----------------+----------------------------------------------------------------

Main |

gdpcfdiff | -.0121929 .0203425 -0.60 0.549 -.0520635 .0276777

gdpcfdifflat | -.0000131 .0005163 -0.03 0.980 -.0010251 .0009989

gdpcsdiff | -.0209543 .020219 -1.04 0.300 -.0605828 .0186743

gdpcsdifflat | -.0003358 .000528 -0.64 0.525 -.0013706 .000699

gdpctdiff | .0004812 .018934 0.03 0.980 -.0366288 .0375912

gdpctdifflat | .0008701 .0005082 1.71 0.087 -.000126 .0018661

opennessfdiff | .0003906 .0058622 0.07 0.947 -.011099 .0118802

opennessfdifflat | -.0000955 .0001966 -0.49 0.627 -.0004809 .0002899

opennesssdiff | -.0104887 .0055337 -1.90 0.058 -.0213346 .0003573

opennesssdifflat | .0003536 .0001777 1.99 0.047 5.31e-06 .0007019

opennesstdiff | .0120029 .0054423 2.21 0.027 .0013363 .0226695

opennesstdifflat | -.0002247 .0001462 -1.54 0.124 -.0005113 .0000619

nrafdiff | .0072821 .0069165 1.05 0.292 -.006274 .0208382

nrafdifflat | -.0001162 .0001408 -0.83 0.409 -.0003921 .0001597

nrasdiff | -.0167994 .0069067 -2.43 0.015 -.0303362 -.0032626

nrasdifflat | .0003979 .000137 2.91 0.004 .0001294 .0006664

nratdiff | -.0005342 .0065261 -0.08 0.935 -.0133252 .0122568

nratdifflat | .000088 .0001316 0.67 0.504 -.0001699 .0003459

tbifdiff | .003963 .002941 1.35 0.178 -.0018013 .0097273

tbifdifflat | -.0000659 .0000489 -1.35 0.177 -.0001618 .0000299

tbisdiff | .0030601 .0030951 0.99 0.323 -.0030062 .0091265

tbisdifflat | -.0000512 .0000514 -1.00 0.319 -.0001521 .0000496

tbitdiff | .0002056 .0028972 0.07 0.943 -.0054729 .005884

tbitdifflat | -3.40e-06 .0000482 -0.07 0.944 -.0000978 .000091

-----------------+----------------------------------------------------------------

Spatial |

rho | .0779905 .1109137 0.70 0.482 -.1393963 .2953774

-----------------+----------------------------------------------------------------

Variance |

sigma2_e | .0000805 3.80e-06 21.16 0.000 .000073 .000088

-----------------+----------------------------------------------------------------

Direct |

gdpcfdiff | -.0125204 .0172662 -0.73 0.468 -.0463616 .0213208

gdpcfdifflat | .0000144 .0004997 0.03 0.977 -.000965 .0009938

gdpcsdiff | -.0199884 .0217321 -0.92 0.358 -.0625826 .0226057

gdpcsdifflat | -.0003465 .0005611 -0.62 0.537 -.0014462 .0007533

gdpctdiff | .0042656 .0185788 0.23 0.818 -.0321482 .0406795

gdpctdifflat | .0008399 .0005121 1.64 0.101 -.0001638 .0018436

opennessfdiff | .0004463 .0062607 0.07 0.943 -.0118245 .0127171

opennessfdifflat | -.0001106 .0001948 -0.57 0.570 -.0004924 .0002713

opennesssdiff | -.0109693 .0050386 -2.18 0.029 -.0208448 -.0010938

opennesssdifflat | .0003642 .0001545 2.36 0.018 .0000614 .0006669

opennesstdiff | .0121712 .0053004 2.30 0.022 .0017825 .0225598

opennesstdifflat | -.0002318 .0001405 -1.65 0.099 -.0005071 .0000434

nrafdiff | .0069549 .0073311 0.95 0.343 -.0074137 .0213235

nrafdifflat | -.0001259 .0001476 -0.85 0.394 -.0004151 .0001634

nrasdiff | -.0156131 .0063218 -2.47 0.014 -.0280036 -.0032226

nrasdifflat | .0003694 .0001293 2.86 0.004 .000116 .0006229

nratdiff | -.0007204 .0072085 -0.10 0.920 -.0148489 .013408

nratdifflat | .0000979 .0001405 0.70 0.486 -.0001775 .0003734

tbifdiff | .0041504 .0028342 1.46 0.143 -.0014045 .0097052

tbifdifflat | -.000069 .0000471 -1.47 0.143 -.0001614 .0000233

tbisdiff | .0030081 .0028058 1.07 0.284 -.0024913 .0085074

tbisdifflat | -.0000504 .0000466 -1.08 0.280 -.0001418 .000041

tbitdiff | .0003346 .0032266 0.10 0.917 -.0059895 .0066587

tbitdifflat | -5.55e-06 .0000536 -0.10 0.918 -.0001107 .0000996

-----------------+----------------------------------------------------------------

Indirect |

gdpcfdiff | -.0007777 .0034101 -0.23 0.820 -.0074613 .005906

gdpcfdifflat | -2.23e-06 .0000868 -0.03 0.980 -.0001724 .0001679

gdpcsdiff | -.0013208 .0043874 -0.30 0.763 -.00992 .0072784

gdpcsdifflat | -.000043 .0001134 -0.38 0.705 -.0002652 .0001793

gdpctdiff | .0001413 .0030166 0.05 0.963 -.0057711 .0060537

gdpctdifflat | .0000813 .0001406 0.58 0.563 -.0001943 .000357

opennessfdiff | 4.86e-07 .0010651 0.00 1.000 -.002087 .002088

opennessfdifflat | -6.40e-06 .0000356 -0.18 0.857 -.0000762 .0000634

opennesssdiff | -.000866 .0016474 -0.53 0.599 -.0040947 .0023628

opennesssdifflat | .0000314 .000055 0.57 0.567 -.0000763 .0001392

opennesstdiff | .0010896 .0018305 0.60 0.552 -.0024982 .0046773

opennesstdifflat | -.0000223 .0000399 -0.56 0.577 -.0001005 .0000559

nrafdiff | .0006545 .0016048 0.41 0.683 -.0024908 .0037999

nrafdifflat | -.0000113 .0000298 -0.38 0.704 -.0000697 .0000471

nrasdiff | -.0015092 .002567 -0.59 0.557 -.0065405 .0035221

nrasdifflat | .0000372 .0000615 0.60 0.545 -.0000833 .0001577

nratdiff | .0000147 .0011923 0.01 0.990 -.0023222 .0023515

nratdifflat | 6.91e-06 .0000256 0.27 0.788 -.0000433 .0000571

tbifdiff | .0004154 .0007729 0.54 0.591 -.0010994 .0019302

tbifdifflat | -6.91e-06 .0000129 -0.54 0.591 -.0000321 .0000183

tbisdiff | .0002826 .0006878 0.41 0.681 -.0010655 .0016307

tbisdifflat | -4.73e-06 .0000115 -0.41 0.680 -.0000272 .0000178

tbitdiff | 7.74e-06 .0004506 0.02 0.986 -.0008754 .0008909

tbitdifflat | -1.27e-07 7.49e-06 -0.02 0.986 -.0000148 .0000146

-----------------+----------------------------------------------------------------

Total |

gdpcfdiff | -.013298 .0193517 -0.69 0.492 -.0512267 .0246306

gdpcfdifflat | .0000122 .0005584 0.02 0.983 -.0010822 .0011066

gdpcsdiff | -.0213092 .0237716 -0.90 0.370 -.0679006 .0252822

gdpcsdifflat | -.0003894 .0006287 -0.62 0.536 -.0016217 .0008429

gdpctdiff | .0044069 .0205825 0.21 0.830 -.035934 .0447478

gdpctdifflat | .0009212 .0005803 1.59 0.112 -.0002161 .0020585

opennessfdiff | .0004468 .0068069 0.07 0.948 -.0128945 .0137881

opennessfdifflat | -.000117 .0002154 -0.54 0.587 -.0005391 .0003052

opennesssdiff | -.0118353 .005327 -2.22 0.026 -.022276 -.0013946

opennesssdifflat | .0003956 .0001699 2.33 0.020 .0000626 .0007286

opennesstdiff | .0132607 .0059003 2.25 0.025 .0016964 .0248251

opennesstdifflat | -.0002541 .0001589 -1.60 0.110 -.0005655 .0000573

nrafdiff | .0076094 .0079382 0.96 0.338 -.0079492 .0231681

nrafdifflat | -.0001372 .0001592 -0.86 0.389 -.0004493 .0001749

nrasdiff | -.0171223 .0074793 -2.29 0.022 -.0317815 -.0024631

nrasdifflat | .0004066 .0001629 2.50 0.013 .0000873 .0007259

nratdiff | -.0007058 .007971 -0.09 0.929 -.0163286 .0149171

nratdifflat | .0001049 .0001548 0.68 0.498 -.0001985 .0004082

tbifdiff | .0045658 .0032604 1.40 0.161 -.0018244 .010956

tbifdifflat | -.000076 .0000542 -1.40 0.161 -.0001822 .0000303

tbisdiff | .0032907 .0031249 1.05 0.292 -.0028341 .0094154

tbisdifflat | -.0000551 .0000519 -1.06 0.289 -.0001569 .0000467

tbitdiff | .0003423 .0034956 0.10 0.922 -.006509 .0071937

tbitdifflat | -5.68e-06 .0000581 -0.10 0.922 -.0001196 .0001082

----------------------------------------------------------------------------------

Consumption SR, Total Crops, 1992 – 2010

SAR with spatial fixed-effects Number of obs = 896

Group variable: id Number of groups = 56

Time variable: year Panel length = 16

R-sq: within = 0.0538

between = 0.1015

overall = 0.0103

Mean of fixed-effects = 0.3676

Log-likelihood = 1349.7163

----------------------------------------------------------------------------------

ydiffsrc | Coef. Std. Err. z P>|z| [95% Conf. Interval]

-----------------+----------------------------------------------------------------

Main |

gdpcfdiff | .0135049 .1211291 0.11 0.911 -.2239038 .2509137

gdpcfdifflat | .0025104 .0030776 0.82 0.415 -.0035216 .0085424

gdpcsdiff | -.0076558 .1204897 -0.06 0.949 -.2438113 .2284996

gdpcsdifflat | -.0051201 .0031625 -1.62 0.105 -.0113185 .0010783

gdpctdiff | -.291363 .1134262 -2.57 0.010 -.5136743 -.0690518

gdpctdifflat | .0070237 .003066 2.29 0.022 .0010144 .013033

opennessfdiff | .0024513 .0349085 0.07 0.944 -.0659681 .0708707

opennessfdifflat | .0000618 .0011707 0.05 0.958 -.0022327 .0023563

opennesssdiff | .0185645 .0329667 0.56 0.573 -.0460491 .0831781

opennesssdifflat | -.0000315 .0010602 -0.03 0.976 -.0021095 .0020465

opennesstdiff | -.0160021 .032377 -0.49 0.621 -.0794599 .0474558

opennesstdifflat | .0004219 .0008701 0.48 0.628 -.0012835 .0021274

nrafdiff | -.0052918 .0411976 -0.13 0.898 -.0860376 .075454

nrafdifflat | .000586 .0008383 0.70 0.485 -.0010571 .0022291

nrasdiff | -.0141142 .0410532 -0.34 0.731 -.094577 .0663487

nrasdifflat | .0001017 .0008135 0.13 0.900 -.0014927 .0016962

nratdiff | -.0222753 .0388853 -0.57 0.567 -.098489 .0539384

nratdifflat | .0000974 .0007836 0.12 0.901 -.0014385 .0016332

tbifdiff | .0022684 .0175185 0.13 0.897 -.0320671 .036604

tbifdifflat | -.0000386 .0002912 -0.13 0.894 -.0006094 .0005321

tbisdiff | .0018979 .0184355 0.10 0.918 -.0342351 .0380308

tbisdifflat | -.0000312 .0003064 -0.10 0.919 -.0006318 .0005694

tbitdiff | -.0031713 .0172516 -0.18 0.854 -.0369839 .0306412

tbitdifflat | .0000529 .0002868 0.18 0.854 -.0005092 .0006149

-----------------+----------------------------------------------------------------

Spatial |

rho | .3962307 .083429 4.75 0.000 .2327129 .5597485

-----------------+----------------------------------------------------------------

Variance |

sigma2_e | .0028561 .0001353 21.11 0.000 .0025909 .0031213

-----------------+----------------------------------------------------------------

Direct |

gdpcfdiff | .0117646 .103778 0.11 0.910 -.1916365 .2151657

gdpcfdifflat | .0026981 .003005 0.90 0.369 -.0031916 .0085878

gdpcsdiff | -.0017551 .1307023 -0.01 0.989 -.2579269 .2544168

gdpcsdifflat | -.0052338 .0033907 -1.54 0.123 -.0118794 .0014118

gdpctdiff | -.2713638 .1122368 -2.42 0.016 -.4913439 -.0513836

gdpctdifflat | .0069044 .0031112 2.22 0.026 .0008065 .0130022

opennessfdiff | .0027899 .0375493 0.07 0.941 -.0708055 .0763852

opennessfdifflat | -.0000269 .0011717 -0.02 0.982 -.0023234 .0022696

opennesssdiff | .0159184 .0302824 0.53 0.599 -.0434341 .0752708

opennesssdifflat | .0000306 .0009304 0.03 0.974 -.0017929 .0018541

opennesstdiff | -.0152065 .0318426 -0.48 0.633 -.0776168 .0472038

opennesstdifflat | .0003841 .0008437 0.46 0.649 -.0012694 .0020376

nrafdiff | -.0073522 .0439775 -0.17 0.867 -.0935466 .0788422

nrafdifflat | .000534 .0008861 0.60 0.547 -.0012028 .0022708

nrasdiff | -.0070931 .0378081 -0.19 0.851 -.0811957 .0670094

nrasdifflat | -.0000681 .0007735 -0.09 0.930 -.001584 .0014479

nratdiff | -.0235752 .0432759 -0.54 0.586 -.1083945 .0612441

nratdifflat | .0001568 .0008446 0.19 0.853 -.0014986 .0018121

tbifdiff | .0034126 .0170342 0.20 0.841 -.0299738 .036799

tbifdifflat | -.0000576 .0002832 -0.20 0.839 -.0006126 .0004975

tbisdiff | .0015775 .0168373 0.09 0.925 -.0314229 .0345779

tbisdifflat | -.0000259 .0002799 -0.09 0.926 -.0005744 .0005226

tbitdiff | -.0024123 .0193861 -0.12 0.901 -.0404084 .0355838

tbitdifflat | .0000403 .0003223 0.12 0.901 -.0005914 .0006719

-----------------+----------------------------------------------------------------

Indirect |

gdpcfdiff | .0107844 .0786196 0.14 0.891 -.1433073 .1648761

gdpcfdifflat | .0017274 .0022205 0.78 0.437 -.0026247 .0060795

gdpcsdiff | .0013499 .0970689 0.01 0.989 -.1889017 .1916014

gdpcsdifflat | -.0035622 .0028134 -1.27 0.205 -.0090764 .0019519

gdpctdiff | -.1821359 .1031465 -1.77 0.077 -.3842992 .0200274

gdpctdifflat | .0045867 .0026018 1.76 0.078 -.0005128 .0096862

opennessfdiff | .001074 .0255627 0.04 0.966 -.0490279 .0511759

opennessfdifflat | 6.58e-06 .0008488 0.01 0.994 -.0016571 .0016702

opennesssdiff | .0119071 .0212776 0.56 0.576 -.0297963 .0536105

opennesssdifflat | .0000114 .0006355 0.02 0.986 -.0012342 .001257

opennesstdiff | -.0108132 .0231316 -0.47 0.640 -.0561503 .034524

opennesstdifflat | .000259 .0006268 0.41 0.679 -.0009695 .0014874

nrafdiff | -.0053949 .028671 -0.19 0.851 -.0615891 .0507992

nrafdifflat | .0003606 .0005929 0.61 0.543 -.0008015 .0015227

nrasdiff | -.0049189 .0272123 -0.18 0.857 -.058254 .0484163

nrasdifflat | -.0000233 .0005799 -0.04 0.968 -.0011598 .0011133

nratdiff | -.0144675 .03051 -0.47 0.635 -.074266 .0453311

nratdifflat | .0000799 .0005987 0.13 0.894 -.0010936 .0012534

tbifdiff | .0028411 .0130386 0.22 0.828 -.0227141 .0283963

tbifdifflat | -.0000478 .0002168 -0.22 0.826 -.0004727 .0003771

tbisdiff | .0011561 .0118838 0.10 0.922 -.0221357 .024448

tbisdifflat | -.000019 .0001975 -0.10 0.923 -.0004061 .0003681

tbitdiff | -.001735 .0130544 -0.13 0.894 -.0273212 .0238512

tbitdifflat | .0000289 .000217 0.13 0.894 -.0003964 .0004543

-----------------+----------------------------------------------------------------

Total |

gdpcfdiff | .022549 .1805221 0.12 0.901 -.3312678 .3763657

gdpcfdifflat | .0044255 .0051214 0.86 0.388 -.0056122 .0144632

gdpcsdiff | -.0004052 .2248431 -0.00 0.999 -.4410897 .4402792

gdpcsdifflat | -.008796 .005991 -1.47 0.142 -.0205382 .0029462

gdpctdiff | -.4534997 .2023022 -2.24 0.025 -.8500046 -.0569947

gdpctdifflat | .0114911 .0053802 2.14 0.033 .0009461 .022036

opennessfdiff | .0038639 .0620451 0.06 0.950 -.1177423 .12547

opennessfdifflat | -.0000203 .0019969 -0.01 0.992 -.0039341 .0038934

opennesssdiff | .0278255 .0507522 0.55 0.584 -.0716471 .1272981

opennesssdifflat | .000042 .0015482 0.03 0.978 -.0029924 .0030764

opennesstdiff | -.0260197 .0540203 -0.48 0.630 -.1318976 .0798582

opennesstdifflat | .0006431 .0014475 0.44 0.657 -.0021939 .00348

nrafdiff | -.0127471 .0716384 -0.18 0.859 -.1531559 .1276616

nrafdifflat | .0008946 .0014515 0.62 0.538 -.0019502 .0037395

nrasdiff | -.012012 .064058 -0.19 0.851 -.1375634 .1135394

nrasdifflat | -.0000913 .0013356 -0.07 0.945 -.0027092 .0025265

nratdiff | -.0380427 .0726356 -0.52 0.600 -.1804059 .1043205

nratdifflat | .0002366 .0014261 0.17 0.868 -.0025585 .0030318

tbifdiff | .0062537 .0297066 0.21 0.833 -.0519702 .0644776

tbifdifflat | -.0001054 .0004939 -0.21 0.831 -.0010734 .0008627

tbisdiff | .0027336 .0282917 0.10 0.923 -.052717 .0581843

tbisdifflat | -.0000449 .0004702 -0.10 0.924 -.0009665 .0008768

tbitdiff | -.0041473 .032138 -0.13 0.897 -.0671366 .058842

tbitdifflat | .0000692 .0005343 0.13 0.897 -.0009779 .0011164

----------------------------------------------------------------------------------

Consumption E, Total Crops, 1992 – 2010

SAR with spatial fixed-effects Number of obs = 896

Group variable: id Number of groups = 56

Time variable: year Panel length = 16

R-sq: within = 0.0520

between = 0.0202

overall = 0.0494

Mean of fixed-effects = 0.0012

Log-likelihood = 1832.8995

----------------------------------------------------------------------------------

ydiffshc | Coef. Std. Err. z P>|z| [95% Conf. Interval]

-----------------+----------------------------------------------------------------

Main |

gdpcfdiff | -.0838611 .0709889 -1.18 0.237 -.2229968 .0552745

gdpcfdifflat | .0019463 .0018007 1.08 0.280 -.001583 .0054757

gdpcsdiff | -.143494 .0704361 -2.04 0.042 -.2815462 -.0054419

gdpcsdifflat | .0052325 .0018418 2.84 0.004 .0016226 .0088424

gdpctdiff | .2076788 .0662004 3.14 0.002 .0779284 .3374292

gdpctdifflat | -.0068796 .0017725 -3.88 0.000 -.0103537 -.0034056

opennessfdiff | .0045661 .0204288 0.22 0.823 -.0354736 .0446059

opennessfdifflat | -.0002524 .0006853 -0.37 0.713 -.0015955 .0010907

opennesssdiff | .0379535 .0192957 1.97 0.049 .0001347 .0757724

opennesssdifflat | -.0017249 .0006195 -2.78 0.005 -.0029391 -.0005106

opennesstdiff | .0091913 .0189515 0.48 0.628 -.0279529 .0463355

opennesstdifflat | -.0001952 .0005093 -0.38 0.702 -.0011934 .0008031

nrafdiff | .0231422 .0241161 0.96 0.337 -.0241244 .0704089

nrafdifflat | -.0003787 .0004904 -0.77 0.440 -.0013398 .0005824

nrasdiff | .0342214 .0240352 1.42 0.155 -.0128868 .0813295

nrasdifflat | -.0009204 .0004765 -1.93 0.053 -.0018543 .0000134

nratdiff | .0543344 .0227697 2.39 0.017 .0097067 .0989622

nratdifflat | -.0012943 .0004588 -2.82 0.005 -.0021936 -.000395

tbifdiff | -.019377 .0102564 -1.89 0.059 -.0394791 .0007251

tbifdifflat | .0003215 .0001705 1.89 0.059 -.0000126 .0006557

tbisdiff | -.0086742 .0107939 -0.80 0.422 -.0298299 .0124814

tbisdifflat | .0001452 .0001794 0.81 0.418 -.0002065 .0004968

tbitdiff | .0053748 .0100984 0.53 0.595 -.0144178 .0251674

tbitdifflat | -.000089 .0001679 -0.53 0.596 -.000418 .00024

-----------------+----------------------------------------------------------------

Spatial |

rho | .0611547 .1096286 0.56 0.577 -.1537133 .2760228

-----------------+----------------------------------------------------------------

Variance |

sigma2_e | .0009787 .0000462 21.16 0.000 .000888 .0010693

-----------------+----------------------------------------------------------------

Direct |

gdpcfdiff | -.0850293 .0602346 -1.41 0.158 -.2030869 .0330283

gdpcfdifflat | .0020437 .0017421 1.17 0.241 -.0013707 .0054581

gdpcsdiff | -.1401876 .0756883 -1.85 0.064 -.288534 .0081588

gdpcsdifflat | .0052006 .0019565 2.66 0.008 .0013659 .0090353

gdpctdiff | .2210569 .0649053 3.41 0.001 .0938448 .3482689

gdpctdifflat | -.006993 .0017851 -3.92 0.000 -.0104918 -.0034942

opennessfdiff | .0047606 .0218192 0.22 0.827 -.0380043 .0475254

opennessfdifflat | -.0003049 .0006792 -0.45 0.653 -.0016362 .0010263

opennesssdiff | .0363353 .017572 2.07 0.039 .0018948 .0707758

opennesssdifflat | -.0016907 .0005394 -3.13 0.002 -.0027479 -.0006336

opennesstdiff | .0097586 .0184865 0.53 0.598 -.0264742 .0459914

opennesstdifflat | -.0002196 .0004894 -0.45 0.654 -.0011788 .0007397

nrafdiff | .0219919 .0255607 0.86 0.390 -.0281062 .0720899

nrafdifflat | -.0004121 .0005147 -0.80 0.423 -.0014208 .0005967

nrasdiff | .0383999 .0219713 1.75 0.081 -.0046631 .0814628

nrasdifflat | -.0010209 .0004491 -2.27 0.023 -.0019011 -.0001406

nratdiff | .053686 .0251341 2.14 0.033 .0044241 .1029478

nratdifflat | -.0012608 .0004904 -2.57 0.010 -.0022219 -.0002997

tbifdiff | -.0187376 .0098852 -1.90 0.058 -.0381124 .0006371

tbifdifflat | .000311 .0001643 1.89 0.058 -.0000111 .0006331

tbisdiff | -.0088905 .0097788 -0.91 0.363 -.0280566 .0102756

tbisdifflat | .0001488 .0001625 0.92 0.360 -.0001698 .0004673

tbitdiff | .0058374 .0112545 0.52 0.604 -.0162211 .0278958

tbitdifflat | -.0000967 .0001871 -0.52 0.605 -.0004634 .00027

-----------------+----------------------------------------------------------------

Indirect |

gdpcfdiff | -.0046836 .0131829 -0.36 0.722 -.0305217 .0211545

gdpcfdifflat | .000125 .0003491 0.36 0.720 -.0005592 .0008092

gdpcsdiff | -.0086715 .0208434 -0.42 0.677 -.0495238 .0321808

gdpcsdifflat | .000347 .0007181 0.48 0.629 -.0010605 .0017545

gdpctdiff | .0148555 .0296851 0.50 0.617 -.0433263 .0730372

gdpctdifflat | -.0004896 .0009531 -0.51 0.607 -.0023577 .0013784

opennessfdiff | .0001157 .0035226 0.03 0.974 -.0067885 .0070199

opennessfdifflat | -8.65e-06 .0001081 -0.08 0.936 -.0002206 .0002033

opennesssdiff | .0031399 .005664 0.55 0.579 -.0079613 .0142411

opennesssdifflat | -.0001304 .0002458 -0.53 0.596 -.0006122 .0003514

opennesstdiff | .0004749 .0028752 0.17 0.869 -.0051603 .0061101

opennesstdifflat | -.0000172 .0000791 -0.22 0.827 -.0001722 .0001377

nrafdiff | .001564 .0047766 0.33 0.743 -.007798 .010926

nrafdifflat | -.000029 .0000905 -0.32 0.748 -.0002063 .0001482

nrasdiff | .0027332 .0061968 0.44 0.659 -.0094123 .0148786

nrasdifflat | -.0000682 .0001544 -0.44 0.659 -.0003708 .0002344

nratdiff | .0044709 .0086675 0.52 0.606 -.0125171 .0214589

nratdifflat | -.000103 .0001992 -0.52 0.605 -.0004934 .0002874

tbifdiff | -.001288 .0029374 -0.44 0.661 -.0070453 .0044692

tbifdifflat | .0000214 .0000488 0.44 0.661 -.0000742 .000117

tbisdiff | -.0005894 .0015752 -0.37 0.708 -.0036767 .002498

tbisdifflat | 9.87e-06 .0000263 0.38 0.707 -.0000416 .0000613

tbitdiff | .000336 .0017125 0.20 0.844 -.0030204 .0036924

tbitdifflat | -5.56e-06 .0000284 -0.20 0.845 -.0000613 .0000502

-----------------+----------------------------------------------------------------

Total |

gdpcfdiff | -.0897128 .0655585 -1.37 0.171 -.2182051 .0387794

gdpcfdifflat | .0021687 .0018984 1.14 0.253 -.0015521 .0058895

gdpcsdiff | -.1488591 .0816529 -1.82 0.068 -.3088958 .0111777

gdpcsdifflat | .0055476 .0021415 2.59 0.010 .0013505 .0097448

gdpctdiff | .2359123 .0721834 3.27 0.001 .0944355 .3773891

gdpctdifflat | -.0074826 .0020509 -3.65 0.000 -.0115023 -.0034629

opennessfdiff | .0048763 .0233028 0.21 0.834 -.0407963 .0505489

opennessfdifflat | -.0003136 .0007377 -0.43 0.671 -.0017594 .0011322

opennesssdiff | .0394753 .0201524 1.96 0.050 -.0000228 .0789733

opennesssdifflat | -.0018211 .000637 -2.86 0.004 -.0030695 -.0005727

opennesstdiff | .0102335 .0196635 0.52 0.603 -.0283062 .0487732

opennesstdifflat | -.0002368 .0005319 -0.45 0.656 -.0012794 .0008057

nrafdiff | .0235559 .0270209 0.87 0.383 -.0294042 .0765159

nrafdifflat | -.0004411 .000543 -0.81 0.417 -.0015054 .0006232

nrasdiff | .0411331 .0243486 1.69 0.091 -.0065893 .0888554

nrasdifflat | -.0010891 .0005025 -2.17 0.030 -.0020739 -.0001043

nratdiff | .0581568 .0294548 1.97 0.048 .0004264 .1158873

nratdifflat | -.0013638 .0005885 -2.32 0.020 -.0025173 -.0002102

tbifdiff | -.0200257 .011012 -1.82 0.069 -.0416087 .0015574

tbifdifflat | .0003323 .0001831 1.82 0.069 -.0000265 .0006911

tbisdiff | -.0094799 .0104582 -0.91 0.365 -.0299776 .0110179

tbisdifflat | .0001587 .0001738 0.91 0.361 -.000182 .0004994

tbitdiff | .0061734 .0120181 0.51 0.607 -.0173817 .0297284

tbitdifflat | -.0001023 .0001998 -0.51 0.609 -.0004938 .0002893

----------------------------------------------------------------------------------
