# Supplementary material for: Commercial Plant Production and Consumption Still Follow the Latitudinal Gradient in Species Diversity despite Economic Globalization
Source: PLoS One. 2016 Oct 5;11(10):e0163002. doi: 10.1371/journal.pone.0163002 (PMC5051709; doi:10.1371/journal.pone.0163002)
Supplement: S2 Table — We present six estimates of model (2) with dependent variables (defined across all plants) in the first column and contemporaneous change and once and twice lagged (L.1 and L.2, respectively) independent variables in the remaining columns where g indicates annual change in logged country-level gross domestic product per capita, o indicates annual change in logged country-level trade openness, and |L| is the absolute value of country capital latitudes (N = 141 and 16 time steps). The first (second) number in each cell gives the estimated coefficient (standard error) times 1000. All coefficient and standard estimates are multiplied by 1000 for readability. Sign and significance data are from the direct SAR estimates of model (2). See Materials and Methods for instructions on interpreting estimated coefficients. Significance levels: ‘***’ 1%, ‘**’ 5%, and ‘*’ 10%. See S1 and S2 Files for model (2) estimate details and S27 File for data used to estimate model (2). (DOCX) [file pone.0163002.s034.docx]

**S2 Table. Estimates of model (2) with contemporaneous and once and twice lagged changes in independent variables.**

|  | **All Plants** | | | | | | | | | | | | | | | | | | |
| --- | --- | --- | --- | --- | --- | --- | --- | --- | --- | --- | --- | --- | --- | --- | --- | --- | --- | --- | --- |
|  | ***g*** | | ***g* x \|*L*\|** | | ***g.L1*** | ***g.L1* x \|*L*\|** | ***g.L2*** | | ***g.L2* x \|*L*\|** | | ***o*** | | ***o* x \|*L*\|** | ***o.L1*** | ***o.L1* x \|*L*\|** | | ***o.L2*** | ***o.L2* x \|*L*\|** | |
|  | **Production** | | | | | | | | | | | | | | | | | | |
| **PSV** | | -1.34 | | 0.09 | -0.79 | -0.01 | | 1.30 | -0.11 | -0.99 | | 0.04 | | -1.27 | | 0.05 | -1.55 | 0.04 | |
|  |  | (3.59) | | (0.13) | (3.71) | (0.12) | | (3.24) | (0.11) | (1.39) | | (0.05) | | (1.22) | | (0.04) | (1.15) | (0.04) | |
| **SR** | | -22.65 | | 0.11 | -5.93 | 0.26 | | -12.77 | 0.15 | 3.62 | | -0.05 | | -4.33 | | 0.31 | 2.00 | -0.17 | |
|  |  | (19.67) | | (0.74) | (20.31) | (0.68) | | (17.77) | (0.61) | (7.64) | | (0.27) | | (6.69) | | (0.21) | (6.28) | (0.21) | |
| **E** | | -34.43** | | 1.79*** | -16.40 | 0.09 | | 16.06 | -0.62 | -0.85 | | -0.09 | | 8.78 | | -0.42** | 4.29 | -0.02 | |
|  |  | (16.88) | | (0.63) | (17.42) | (0.58) | | (15.23) | (0.53) | (6.56) | | (0.23) | | (5.73) | | (0.18) | (5.39) | (0.18) | |
|  | | **Consumption** | | | | | | | | | | | | | | | | | |
| **PSV** | | 9.49 | | -0.34 | -0.79 | 0.13 | 4.28 | | -0.34 | | -2.14 | | 0.14 | 3.74 | -0.15 | | 2.36 | | -0.07 |
|  |  | (10.22) | | (0.38) | (10.56) | (0.35) | (9.23) | | (0.32) | | (3.97) | | (0.14) | (3.48) | (0.11) | | (3.27) | | (0.11) |
| **SR** | | 121.37 | | -1.17 | 140.83* | 1.54 | 89.94 | | 2.56 | | -6.21 | | 1.14 | -18.17 | 1.34* | | 27.96 | | -0.80 |
|  |  | (75.48) | | (2.82) | (77.98) | (2.60) | (68.31) | | (2.35) | | (29.26) | | (1.03) | (25.62) | (0.81) | | (24.09) | | (0.80) |
| **E** | | 6.00 | | 0.07 | -11.23 | -1.21 | 26.26 | | 1.51 | | 20.81 | | -0.87 | 12.26 | -0.36 | | 14.10 | | 0.13 |
|  |  | (39.22) | | (1.47) | (40.51) | (1.35) | (35.44) | | (1.22) | | (15.24) | | (0.53) | (13.33) | (0.42) | | (12.53) | | (0.42) |
