# Supplementary material for: Commercial Plant Production and Consumption Still Follow the Latitudinal Gradient in Species Diversity despite Economic Globalization
Source: PLoS One. 2016 Oct 5;11(10):e0163002. doi: 10.1371/journal.pone.0163002 (PMC5051709; doi:10.1371/journal.pone.0163002)
Supplement: S3 Table — We present six estimates of model (3) with dependent variables (defined across all plants) in the first column and contemporaneous change and once lagged (L.1) independent variables in the remaining columns where g indicates annual change in logged country-level gross domestic product per capita, o indicates annual change in logged country-level trade openness, nra indicates annual change in country-level nominal rate of assistance, tbi indicates annual change in trade bias index, and |L| is the absolute value of country capital latitudes (N = 56 and 17 time steps). The first (second) number in each cell gives the estimated coefficient (standard error) times 1000. All coefficient and standard estimates are multiplied by 1000 for readability. Sign and significance data are from the direct SAR estimates of model (3). See Materials and Methods for instructions on interpreting estimated coefficients. Significance levels: ‘***’ 1%, ‘**’ 5%, and ‘*’ 10%. See S1 and S2 Files for model (3) estimate details and S29 File for data used to estimate model (3). (DOCX) [file pone.0163002.s035.docx]

**S3 Table. Estimates of model (3) with contemporaneous and once lagged changes in independent variables.**

|  | **All Plants** | | | | | | | |
| --- | --- | --- | --- | --- | --- | --- | --- | --- |
|  | ***g*** | ***g* x \|*L*\|** | ***g.L1*** | ***g.L1* x \|*L*\|** | ***o*** | ***o* x \|*L*\|** | ***o.L1*** | ***o.L1* x \|*L*\|** |
|  | **Production** | | | | | | | |
| **PSV** | 5.40  (6.66) | -0.10 (0.19) | 1.93 (7.91) | -0.12 (0.19) | -0.73 (2.13) | 0.03 (0.07) | 1.43 (2.23) | -0.03 (0.06) |
| **SR** | -64.86 (40.71) | 0.68 (1.14) | 40.8 (48.29) | -1.39 (1.17) | 2.85 (12.97) | 0.41 (0.44) | 18.21 (13.63) | -0.52 (0.34) |
| **E** | -88.81*** (24.64) | 3.08*** (0.7) | -6.11 (29.1) | 1.14 (0.70) | -2.43 (7.81) | -0.01 (0.26) | -5.06 (8.21) | 0.09  (0.21) |
|  | **Consumption** | | | | | | | |
| **PSV** | -0.76 (16.87) | -0.47 (0.47) | -19.22 (20.03) | 0.32 (0.48) | 0.86 (5.37) | -0.02 (0.18) | -4.33 (5.65) | -0.12 (0.14) |
| **SR** | 154.57 (102.06) | 0.05 (2.87) | -28.90 (121.0) | -3.01 (2.93) | -24.9 (32.44) | 0.90 (1.10) | 61.28* (34.12) | -1.79** (0.86) |
| **E** | -50.12 (60.39) | 1.92 (1.70) | -75.93 (71.64) | 1.16 (1.74) | 14.24 (19.24) | -0.44 (0.65) | -14.52 (20.22) | 0.99* (0.51) |

|  | **All Plants** | | | | | | | |
| --- | --- | --- | --- | --- | --- | --- | --- | --- |
|  | ***nra*** | ***nra* x \|*L*\|** | ***nra.L1*** | ***nra.L1* x \|*L*\|** | ***tbi*** | ***tbi* x \|*L*\|** | ***tbi.L1*** | ***tbi.L1* x \|*L*\|** |
|  | **Production** | | | | | | | |
| **PSV** | -0.52  (2.40) | 0.02  (0.05) | 0.85  (2.32) | 0.00  (0.05) | -0.31 (1.20) | 0.01  (0.02) | 0.63 (1.07) | -0.01  (0.02) |
| **SR** | -4.79 (14.7) | 0.14  (0.29) | -1.06 (14.2) | -0.03  (0.29) | 0.15 (7.37) | 0.00  (0.12) | 2.42 (6.57) | -0.04  (0.11) |
| **E** | -0.80 (8.84) | -0.01  (0.17) | 2.45  (8.55) | -0.28  (0.18) | 0.79 (4.43) | -0.01  (0.07) | 1.30 (3.95) | -0.02  (0.07) |
|  | **Consumption** | | | | | | | |
| **PSV** | 1.05  (6.09) | 0.04  (0.12) | -16.18*** (5.88) | 0.30**  (0.12) | 2.74 (3.05) | -0.05  (0.05) | 3.52 (2.72) | -0.06 (0.05) |
| **SR** | -40.73 (36.85) | 1.19  (0.73) | 23.53 (35.55) | -0.82  (0.73) | -1.92 (18.45) | 0.030  (0.31) | 8.32 (16.44) | -0.14 (0.27) |
| **E** | 44.6** (21.8) | -0.91** (0.43) | 12.09 (21.1) | -0.25  (0.43) | -18.15* (10.92) | 0.30*  (0.18) | -7.46 (9.74) | 0.12  (0.16) |
